# Multicomponent and multicatalytic asymmetric synthesis of furo[2,3-*b*]pyrrole derivatives: further insights into the mode of action of chiral phosphoric acid catalysts†‡

**DOI:** 10.1039/d0sc03342a

**Published:** 2020-08-12

**Authors:** Lara Cala, Pedro Villar, Ángel R. de Lera, Francisco J. Fañanás, Rosana Álvarez, Félix Rodríguez

**Affiliations:** Instituto Universitario de Química Organometálica “Enrique Moles”, Centro de Innovación en Química Avanzada (ORFEO-CINQA), Universidad de Oviedo Julián Clavería, 8 33006-Oviedo Spain frodriguez@uniovi.es; Departamento de Química Orgánica (CINBIO), Universidade de Vigo As Lagoas-Marcosende E-36310 Vigo Spain rar@uvigo.es

## Abstract

Multicomponent and multicatalytic reactions are those processes that try to imitate the way the enzymatic machinery transforms simple building blocks into complex products. The development of asymmetric versions of these reactions is a step forward in our dream of mirroring the exquisite selectivity of biological processes. In this context, the present work describes a new reaction for the asymmetric synthesis of furo[2,3-*b*]pyrrole derivatives from simple 3-butynamines, glyoxylic acid and anilines in the presence of a dual catalytic system, formed from a gold complex and a chiral phosphoric acid. Computations, aimed to understand the exceptional performance of 9-anthracenyl-substituted BINOL-derived phosphoric acid catalyst, suggest a fundamental role of non-covalent interactions being established between the catalyst and the reagents for the outcome of the multicomponent process. The linear geometry of the anthracenyl substituent along with the presence of an electron-withdrawing group in the aniline and an aromatic substituent in the 3-butynamine derivative seem to be key structural factors to explain the experimental results and, particularly, the high stereoselectivity.

## Abstract 2

Furo[2,3-*b*]pyrroles are selectively synthesized by a multicomponent reaction catalysed by a gold/chiral phosphoric acid system. The non-covalent interactions established between the acid and the reagents explain the exquisite stereoselectivity.

## Graphical abstract

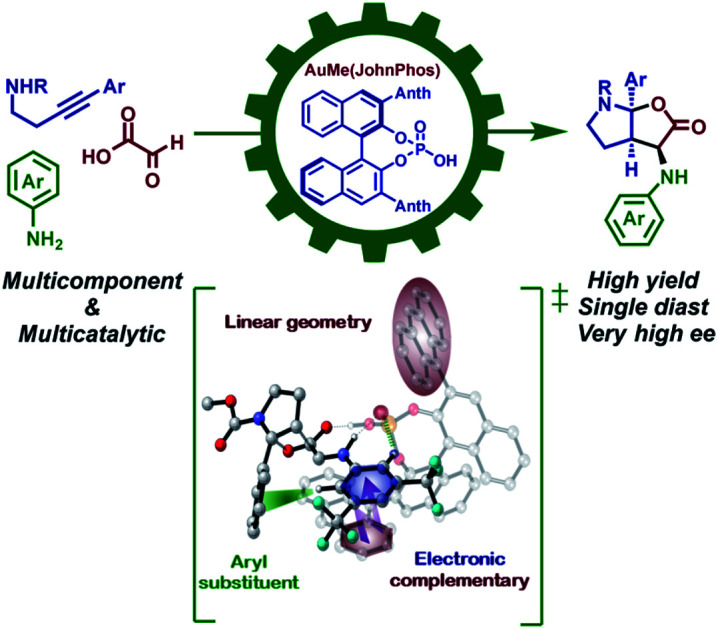

## Introduction

**Scheme 1:**
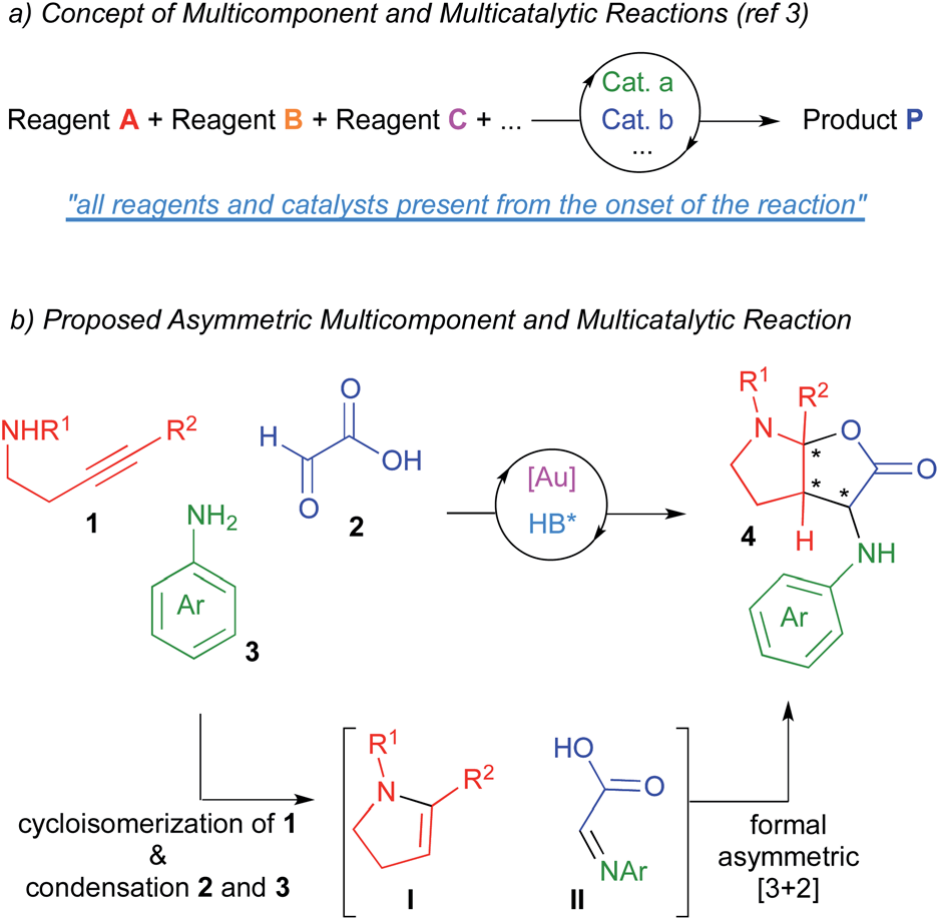
Multicomponent and multicatalytic reactions. Concept and our proposal.

Catalysis has become, without question, a major subject of modern chemistry. In traditional catalytic strategies, a single catalyst interacts with a reagent lowering the energetic barrier of the subsequent reaction with another reagent. Although this strategy has delivered vast numbers of new reactions over many decades, new directions in the field of catalysis are necessary in order to address the demands of sustainability and global well-being of our society. In this context, multicatalytic reactions (one-pot catalysis), defined as those processes where several reactions occur in a single flask as a result of the action of multiple catalyst, have become a powerful synthetic tool.^1^ On the other hand, multicomponent reactions, which are those processes where several starting materials react together to yield a product that retains the majority of atoms of the reactants, have also become of significant interest in the field of organic synthesis.^2^ As a result of merging both tools, multicomponent and multicatalytic reactions have recently emerged as one of those new concepts in the area of catalysis.^3^ In these processes, three or more starting materials react by means of two or more catalysts (all present from the onset of the reaction) to produce structurally and functionally complex products (Scheme 1a). These reactions try to mimic the way the enzymatic machinery (multicatalytic system) transforms in Nature a series of simple molecules (multicomponent system) into intricate structures. The exquisite stereoselectivity of biosynthetic reactions is particularly difficult to imitate and thus, the development of asymmetric multicomponent and multicatalytic reactions remains challenging.^4^

In this context and based on previous works form our laboratories,^5^ we envisioned that the multicomponent coupling of alkynamines **1**, glyoxylic acid **2** and anilines **3** in the presence of a multicatalytic gold/chiral Brønsted acid system could deliver enantioenriched furo[2,3-*b*]pyrrole derivatives **4** comprising three contiguous stereocenters (one of them quaternary; Scheme 1b). We thought that the gold-catalysed cycloisomerization reaction of alkynamines **1** should deliver the enamine intermediates **I**.^6^ Moreover, the condensation reaction between glyoxylic acid and amines **3** should give imines **II**. Further asymmetric reaction between these *in situ* formed intermediates would lead to our desired furo[2,3-*b*]pyrrole derivatives **4**, which are targets of interest owing to the prevalence of this structural motif in numerous biologically pharmacophores and natural products.^7^

In addition to reporting the development of this process, we are also presenting computational studies aimed to structurally justify the origin of the very high diastereo and enantioselectivities observed with just a single catalyst of the series of phosphoric acids surveyed, thus providing new vistas to rationalize the behaviour of chiral phosphoric acids in asymmetric processes.^8^

## Results and discussion

At the outset of this study, we selected *tert*-butyl[4-(4-chlorophenyl)-3-butyn-1-yl]carbamate **1a**, glyoxylic acid **2** and 3-nitroaniline **3a** as model reagents in order to find optimal reaction conditions. As multicatalytic system, we chose a combination of AuMe(JohnPhos) and a Brønsted acid (HB; particularly, phosphoric acid derivatives). Initial experiments were performed at room temperature, in toluene as solvent and in the presence of 4 Å molecular sieves (Table 1).

**Table 1:** Screening of Brønsted acid catalysts

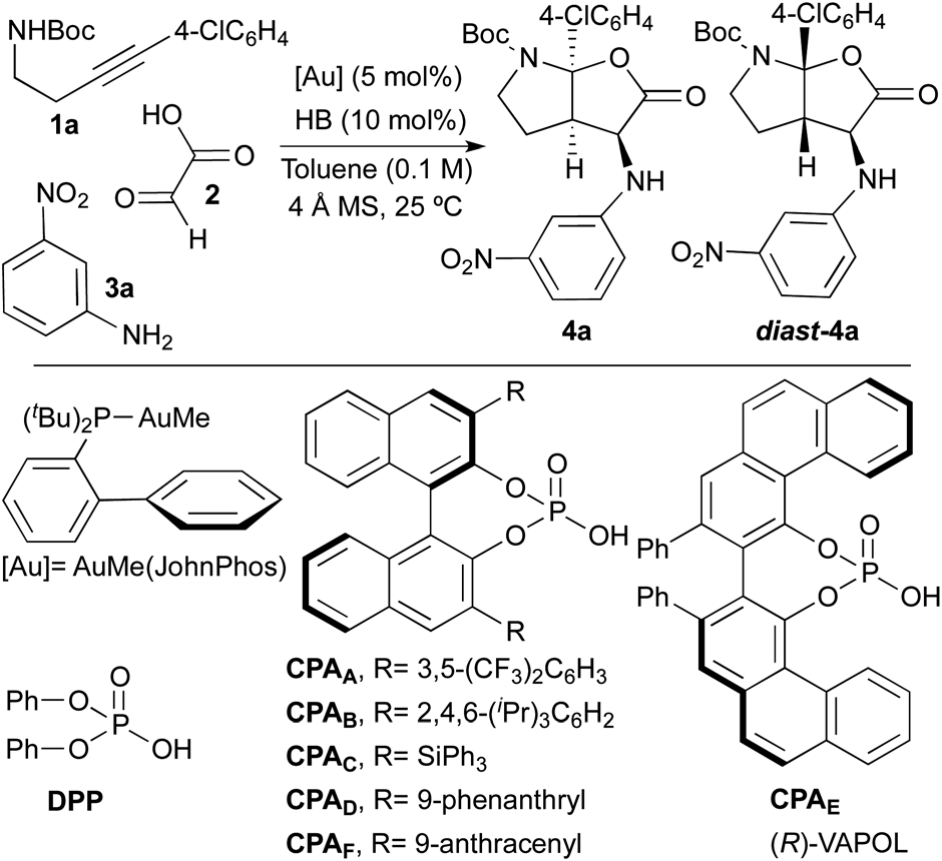					
Entry	HB	Yield^a^ (%)	d.r. (**4a** : **diast-4a**)^b^	e.r. (**4a**)^c^	e.r. (**diast-4a**)^c^
1	**DPP**	91	1 : 1.5	—	—
2	**CPAA**	88	1.3 : 1	86 : 14	36 : 64
3	**CPAB**	91	1.3 : 1	49 : 51	32 : 68
4	**CPAC**	95	1 : 1	74 : 26	50 : 50
5	**CPAD**	92	1.9 : 1	99 : 1	47 : 53
6	**CPAE**	87	1 : 1.4	19 : 81	4 : 96
7	**CPAF**	98	>20 : 1	99 : 1	—

a Based on **1a**.

b Determined by ^1^H NMR analysis of the crude reaction mixture.

c Determined by HPLC using a chiral stationary phase.

To check the viability of our proposal, we firstly tried the reaction in its racemic version by using diphenyl hydrogen phosphate [**DPP**, (PhO)_2_PO_2_H] as the acid catalyst. Although the reaction led to the desired product in high yield (91%), unfortunately, an almost equimolar mixture of the two diastereoisomers **4a** and **diast-4a** was obtained (d.r. = 1 : 1.5; Table 1, entry 1). Considering that our objective was the development of a truly selective reaction (diastereo and enantioselective), this preliminary result was highly discouraging since we suspected that the use of chiral phosphoric acid-derived catalysts (CPAs) to perform the asymmetric version of the reaction, could in turn lead to the same diastereoselectivity problems. In fact, when we tried the reaction with BINOL-derived phosphoric acids substituted at C3- and C3′-positions with 3,5-bis(trifluoromethyl)phenyl (**CPAA**), 2,4,6-triisopropylphenyl (**CPAB**) or triphenylsilyl (**CPAC**) groups, we also observed the formation of roughly equimolar amounts of the two diastereoisomers **4a** and **diast-4a** (Table 1, entries 2–4). Furthermore, the low levels of enantioselectivity found in both **4a** and **diast-4a** were additional discouraging issues at this point.

Although the same problems of diastereoselectivity were observed when using the phenanthryl-substituted BINOL-derivative **CPAD** and the VAPOL-derived phosphoric acid **CPAE** (d.r. = 1.9 : 1 and 1 : 1.4 respectively; Table 1, entries 5 and 6), at least in these cases one of the diastereoisomers was obtained with high enantioselectivity [e.r.(**4a**) = 99 : 1 with **CPAD**; e.r.(**diast-4a**) = 96 : 4 with **CPAD**]. Nevertheless, none of the above mentioned phosphoric acid catalysts led to satisfactory results in terms of selectivity. Surprisingly, when the reaction was performed with the 9-anthracenyl-substituted BINOL-derived phosphoric acid **CPAF**, we only observed the formation of compound **4a** in almost quantitative yield (98%) and, more importantly, with complete stereoselectivity (d.r. > 20 : 1; e.r. = 99 : 1).

The extraordinary performance of catalyst **CPAF** should be remarked. Thus, while all other acid catalysts (achiral or chiral) produced basically equimolecular mixtures of two diastereoisomers, **CPAF** proved unique in performing this transformation in a highly diastereoselective way. The very high enantioselectivity achieved with this catalyst (only one enantiomer was observed) should also be noted. Thus, from the eight possible stereoisomers, we only observed the formation of one of them.

**Scheme 2:**
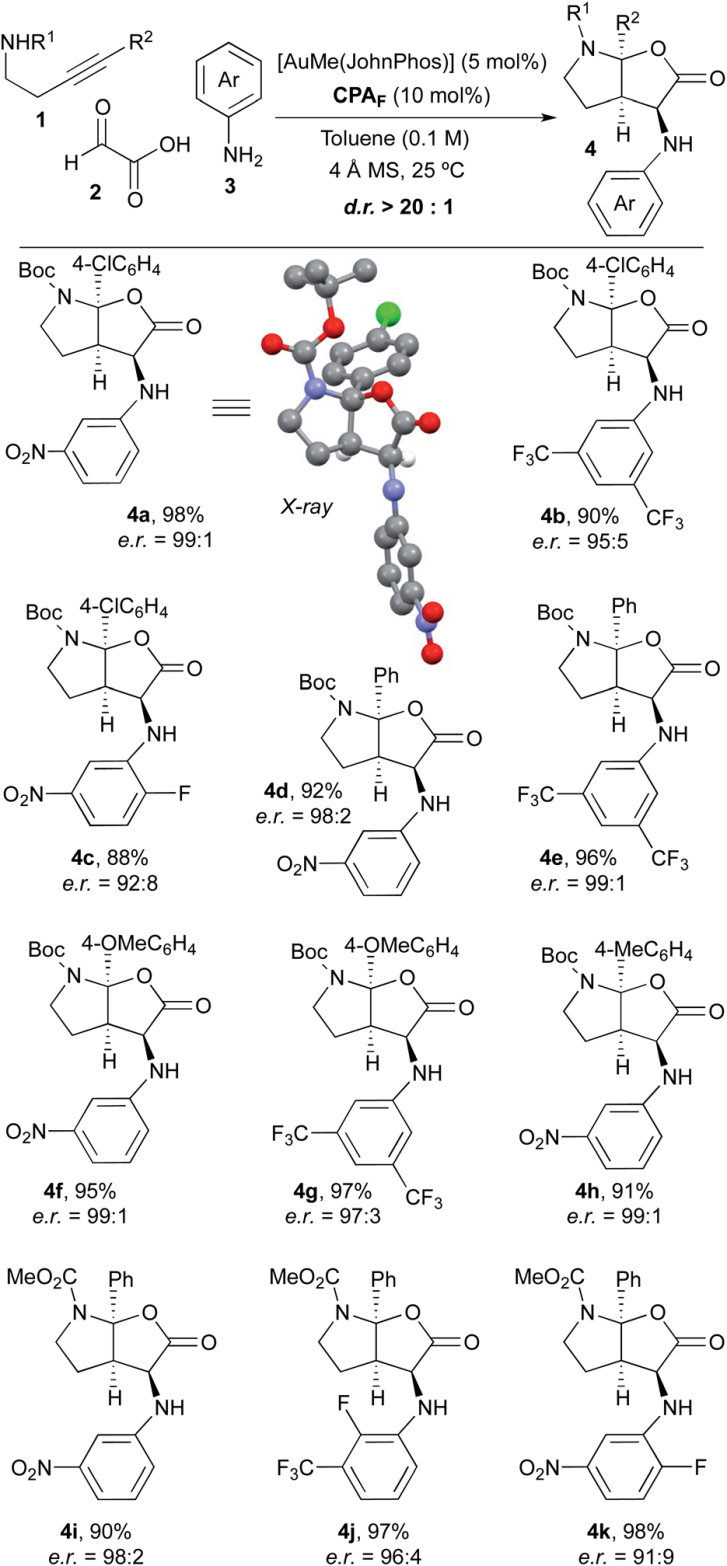
Scope of the reaction.

Once we had verified that the 9-anthracenyl-substituted BINOL-derived phosphoric acid **CPAF** was an optimal acid catalyst to perform the desired asymmetric multicomponent and multicatalytic process, we addressed the scope of the transformation. Thus, a range of experiments were performed by mixing different 4-arylbut-3-yn-1-amines **1**, glyoxylic acid **2** and aniline derivatives **3** in toluene (0.1 M) with 4 Å molecular sieves at room temperature in the presence of a mixture of AuMe(JohnPhos) (5 mol%) and chiral phosphoric acid **CPAF** (10 mol%; Scheme 2). We were delighted to find that, under these conditions, a series of furo[2,3-*b*]pyrrole derivatives **4** with diverse substitution patterns were obtained in excellent yields, as single diastereoisomers and with very high enantioselectivities in most cases. Alkynamines **1** substituted at the triple bond with different aromatic rings likewise performed efficiently and led to the corresponding products **4** with very high selectivity.^9^ Regarding the substitution on the nitrogen atom, *tert*-butoxycarbonyl (**4a–h**) and methoxycarbonyl groups (**4i–k**) were used. Anilines **3** bearing electron-withdrawing substituents were appropriate substrates. However, the reaction did not proceed when simple aniline or aniline derivatives containing electron-donating groups were used. The reaction with alkylamine derivatives instead of anilines was unsuccessful. The structure and absolute configuration of **4a** was unambiguously determined by X-ray crystallographic analysis.^10^ The absolute configuration of the remaining furo[2,3-*b*]pyrrole derivatives **4** was assigned by analogy.

**Scheme 3:**
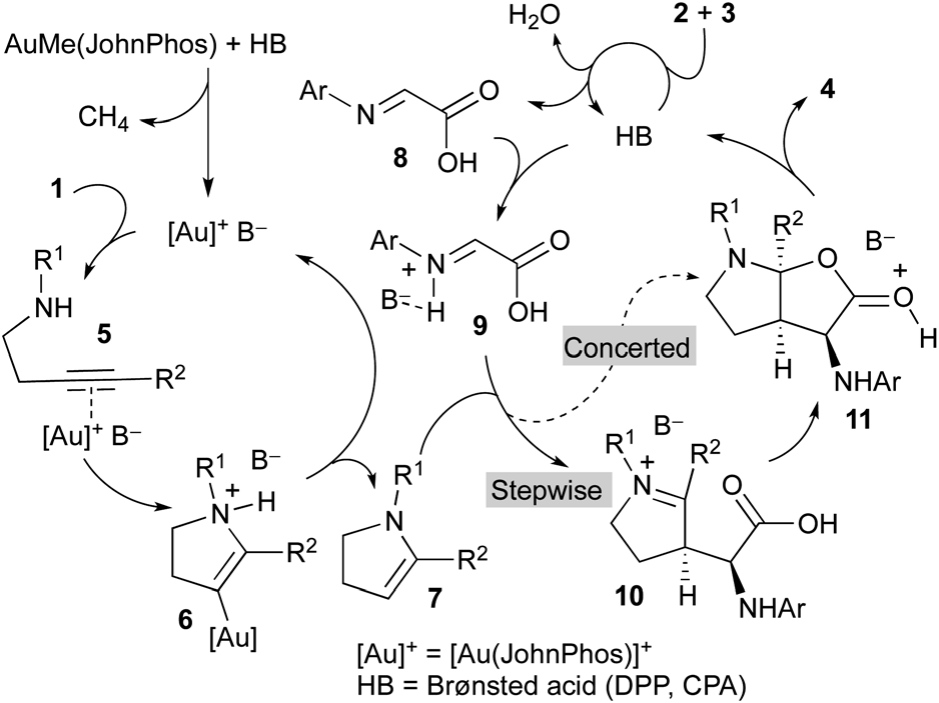
Mechanistic proposal.

A mechanistic proposal to explain this multicomponent and multicatalytic reaction is shown in Scheme 3. We considered the initial cycloisomerization of amine derivative **1** to give the enamine **7** in a process promoted by the cationic gold(i) complex generated from AuMe(JohnPhos) by reaction with the Brønsted acid (**DPP** or **CPA**). Thus, the initial coordination of the gold catalyst to the triple bond of **1** leads to intermediate **5**. Intramolecular addition of the amino group to the distal carbon of the activated alkyne generates intermediate **6**. Finally, a protodemetallation process, likely assisted by the anion (B^−^), affords the enamine derivative **7** regenerating the gold catalyst and closing the first catalytic cycle. Simultaneously, the Brønsted acid catalyst (HB) promotes the condensation of glyoxylic acid **2** and aniline derivative **3** to form imine **8**.

Both intermediates generated in the two previous independent processes, namely the *N*-protected 2,3-dihydro-1*H*-pyrrole **7** and the 2-(arylimino)acetic acid **8**, enter a third catalytic cycle where the three stereogenic centres of the final product **4** are stereoselectively generated. Being both components achiral, the chiral Brønsted acid catalyst (HB) should be involved in this process. Thus, reaction of the imine **8** with the acid affords activated imine derivative **9**. The subsequent reaction of this intermediate with enamine **7** could occur through a concerted mechanism to directly deliver intermediate **11**, which evolves providing the final product **4** and regenerating the acid catalyst. Alternatively, intermediate **11** could be formed through a stepwise process initiated by a Mannich-type addition to produce iminium ion intermediate **10** followed by a cyclization reaction. Regardless of the mechanism, concerted or stepwise, the extraordinary efficiency of the chiral acid catalyst **CPAF** should be remarked because it favours the reaction through a single face of the activated imine **9** and a single face of the enamine **7** to form all three stereogenic centres of the final product **4** with exquisite selectivity.

The high specificity exhibited by catalyst **CPAF** is probably the most remarkable feature of the reaction here described. With this catalyst, we observed very high diastereo and enantioselectivities. On the contrary, basically equimolecular mixtures of diastereo and/or enantiomers were observed with the other catalysts used (Table 1). Although variations in stereoselectivity values are usually observed upon changing the substituents at the 3- and 3′-positions of BINOL-derived phosphoric acids (**CPA**), the contrasting differences we observed are unusual. Apparently, the specificity exhibited by the 9-anthracenyl-substituted BINOL-derived phosphoric acid **CPAF** cannot be just attributed to steric effects because other catalysts containing large groups at 3- and 3′-positions, *i.e.* the triphenylsilyl-substituted **CPAC**, did not perform satisfactorily. It is also surprising that phenanthryl-substituted chiral phosphoric acid **CPAD**, apparently very similar to **CPAF**, behaves so differently. Intrigued, we wondered why the 9-anthracenyl-substituted BINOL-derived phosphoric acid **CPAF** was so specific, and we considered performing a computational study to find the particular features of this catalyst in our reaction. In a more general sense, we also pursued with this investigation to better understand the behaviour of BINOL-derived phosphoric acids in asymmetric processes.

**Scheme 4:**
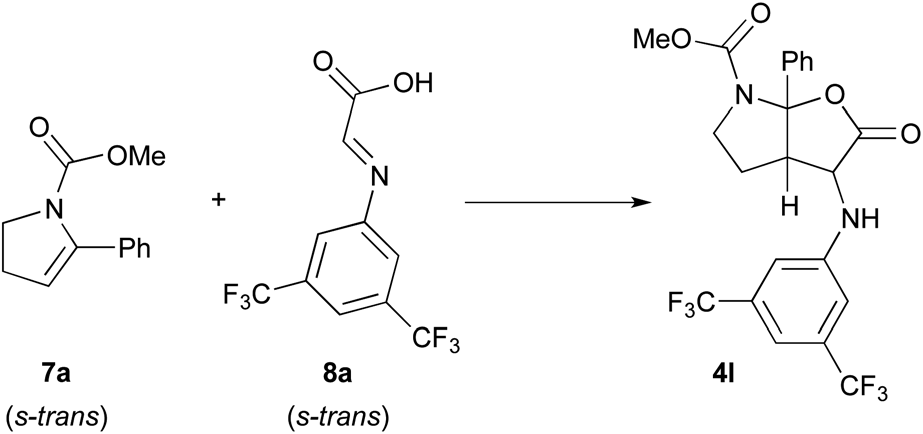
Model reaction selected for the computational studies.

In this context, the reaction of methyl 5-phenyl-2,3-dihydro-1*H*-pyrrole-1-carboxylate **7a** and imine **8a** derived from the condensation of glyoxylic acid and 3,5-bis(trifluoromethyl)phenylaniline was selected as model to perform DFT calculations (Scheme 4).^11^ This model reaction, with both reagents in their more stable *s-trans* conformation, mimics the third catalytic cycle of the mechanism shown in Scheme 3 where all stereocenters of the final product **4** are generated.

**Scheme 5:**
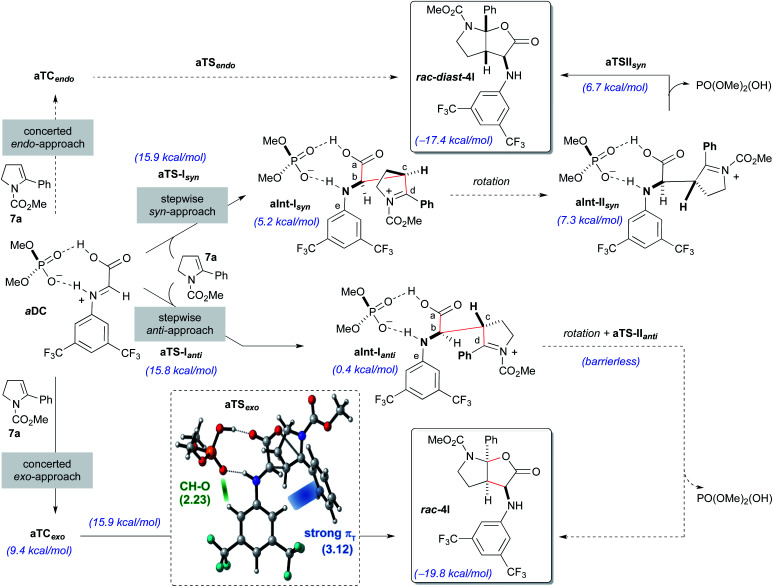
**DMP**-promoted reaction of **8a** and **7a** by concerted and stepwise mechanisms computed at the [wB97XD/DEF2TZVPP(SMD,toluene)//wB97XD/DEF2SVPP(SMD,toluene)] level of theory. Transition state **aTSexo** with distances indicated in Å, computed at the level shown above, is highlighted.

Initially, the reaction with the achiral acid catalyst dimethyl hydrogen phosphate (**DMP**; Scheme 5) was studied. Formation of a binary complex (**aDC**), in which the acid interacts with the imine **8a** in the *s-trans* conformation through two hydrogen bonds in a bifunctional activation fashion, was considered to be the most likely. Two alternative mechanisms, namely concerted and stepwise, for the reaction of the binary complex **aDC** with enamine derivative **7a** to give the final racemic products **rac-4l** or **rac-diast-4l** were evaluated. For the concerted [3 + 2]-cycloaddition mechanism, and prior to bond formation, we considered the formation of two possible three-component complexes (**aTCendo** and **aTCexo**), which would be created when **aDC** and **7a** approach each other. These two three-component complexes differ by the orientation of the phenyl group of the enamine **7a** when facing **aDC** [inwards (*endo*) or outwards (*exo*) the catalytic region]. Formation of **rac-4l** or **rac-diast-4l** from **aTCexo** or **aTCendo** proceeds through transition states **aTSexo** or **aTSendo**, respectively. Despite our efforts, only the concerted *exo*-approach could be characterized, and transition state **aTSexo** was found to be highly asynchronous with a computed activation energy of 15.9 kcal mol^−1^ (for details see the ESI‡). Detailed structural analysis of **aTSexo** allowed to identify a stabilizing C_Ar_H–π interaction (3.12 Å), the so-called T-shaped edge-to-face interaction (Scheme 5; blue strand in **aTSexo**).^12^ Taking into account that this arene–arene interaction is electrostatic, the presence of the trifluoromethyl substituents contributes to decrease the electron density of the C_Ar_–H bonds favouring the mentioned interaction. We also noticed that the orientation of the 3,5-bis(trifluoromethyl)phenyl group appears to be controlled by hydrogen bonding interactions between one of the hydrogen atoms at the *ortho*-position and one of the oxygen atoms of the phosphoric acid (2.23 Å, Scheme 5, green strand in **aTSexo**).

In contrast, the *endo*-approach does not allow similar arene–arene stabilizing interactions and that might explain why we were unable to find the alternative concerted *endo*-approach pathway.

The stepwise mechanism for the [3 + 2]-cycloaddition could take place through two alternative orientations of the enamine **7a** relative to the binary complex **aDC** (*syn*- or *anti*-approaches; Scheme 5). The reaction was considered to start with the stereodetermining nucleophilic addition of enamine **7a** through its C3-position (C_*c*_) to the electrophilic carbon (C_*b*_) of the iminium ion of the **aDC** complex. Different orientations of the enamine **7a** and complex **aDC**, as well as the *s-cis* and *s-trans* conformations of the imine **8a** were taken into consideration. Several transition states, which showed different conformations around the dihedral angle C_*a*_C_*b*_C_*c*_C_*d*_ and the *s-cis* and *s-trans* conformers of the carbamate, were optimized and turned out to be almost isoenergetic (ΔΔ*G* = 2.0–0.1 kcal mol^−1^). The lowest energy transition state for each approach (**aTS-Isyn** and **aTS-Ianti**) were selected and found to have very similar activation energies (15.9 and 15.8 kcal mol^−1^ respectively). Once the first intermediates **aInt-Isyn** and **aInt-Ianti** are formed (note that these intermediates are similar to **10** in Scheme 3), a conformational change by bond rotation of dihedral angles C_*a*_C_*b*_C_*c*_C_*d*_ and C_*a*_C_*b*_NC_*e*_ should occur to allow the nucleophilic attack of the carboxylate onto the iminium ion (Scheme 5). Initially, we assumed that the energy required for the conformational change from **aInt-I** to **aInt-II** could not compete with the energy required for the C–C bond formation.

For the *syn* approach, we were able to characterize the intermediate **aInt-IIsyn** and the transition state **aTS-IIsyn** corresponding to the formation of the C–O bond. However, the energy of this transition state (6.7 kcal mol^−1^) is lower than that of intermediate **aInt-IIsyn** (7.5 kcal mol^−1^), suggesting that, under standard conditions, the second bond formation is a barrierless process. Interestingly, for the alternative *anti*-approach, the corresponding intermediate **aInt-IIanti** and the subsequent C–O bond formation transition state **aTS-IIanti** could not be characterized and we observed the direct formation of the final product from **aInt-Ianti** in a barrierless process. Most likely, the proximity of the electrophilic iminium carbon (C_*d*_) and the carboxylate favours the collapse and fast formation of the C–O bond.

Considering the global study, the rate-determining steps of the processes going through **aTSexo** (15.9 kcal mol^−1^), **aTS-Ianti** (15.8 kcal mol^−1^) and **aTS-Isyn** (15.9 kcal mol^−1^) were isoenergetic and therefore, a low *anti*/*syn* diastereoisomeric ratio was predicted (d.r. = 2.1 : 1) for the formation of the furo[2,3-*b*]pyrrole derivatives **rac-4l** or **rac-diast-4l**. This is in complete agreement with our experimental results (see Table 1, entry 1).

**Fig. 1:**
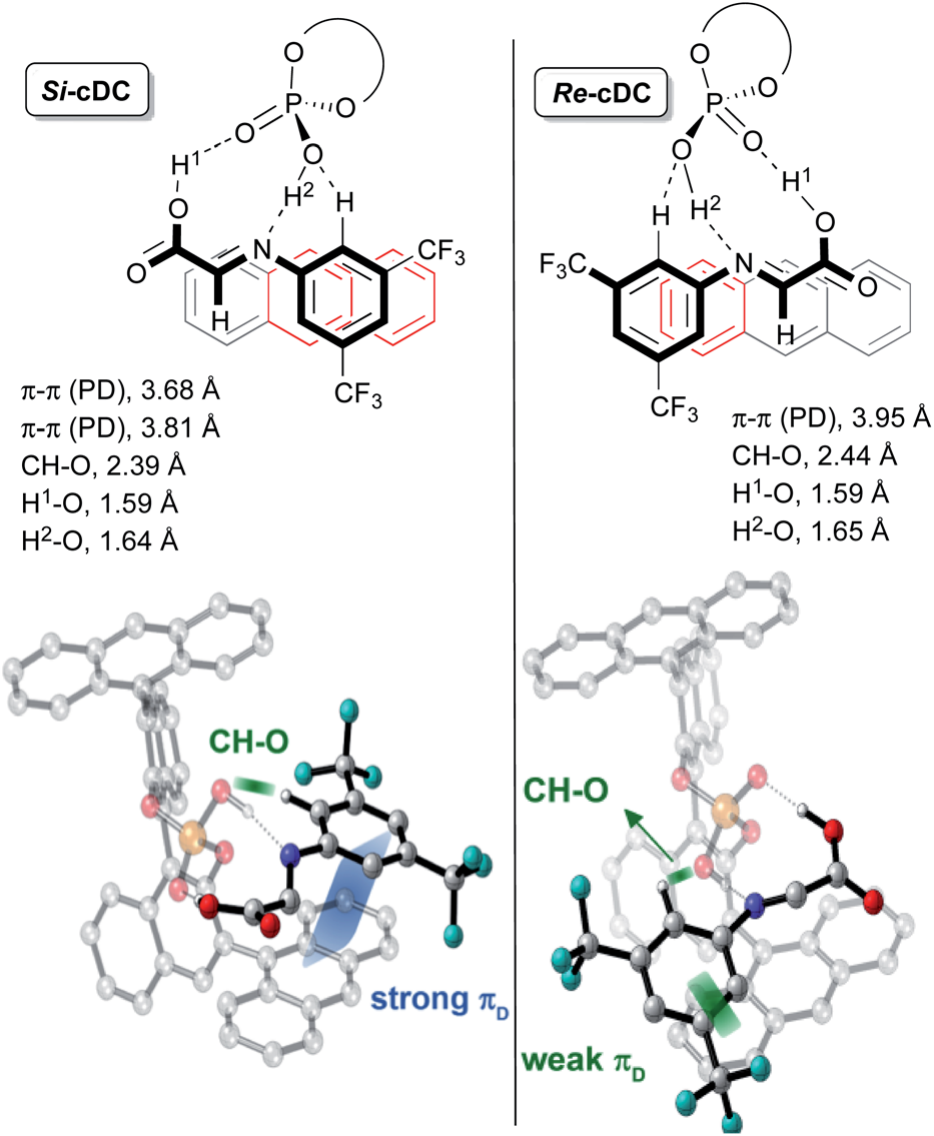
Representation of binary complexes *Si*-**cDC** and *Re*-**cDC** computed at the wB97XD/DEF2TZVPP(SMD,toluene)//wB97XD/DEF2SVPP (SMD,toluene) level of theory for (*R*)-3,3′-bis(9-anthracenyl)-1,1′-binaphthyl-2,2′-diyl hydrogenphosphate as **CPAF**.

This initial study performed with the achiral catalysts dimethyl hydrogen phosphate (**DMP**) served not only to verify the agreement between the computational results and the experimental data but also to show that our reaction could proceed through alternative concerted and/or stepwise mechanisms. With this information in hand, we faced the more challenging study on the origin of the diastereo and enantioselectivity of the formal [3 + 2]-cycloaddition when performed with the chiral 9-anthracenyl-substituted BINOL-derived phosphoric acid catalyst **CPAF**. More precisely, we evaluated the reaction between the enamine derivative **7a** and a chiral binary complex (**cDC**), itself formed by the interaction of the imine **8a** with **CPAF**. The two possible diastereomeric chiral binary complexes *Re*-**cDC** and *Si*-**cDC**, generated by the interaction of chiral acid **CPAF** and imine **8a** (Fig. 1) were then analysed. These two complexes differ by the facial orientation of the imine towards the phosphoric acid. Whereas in *Re*-**cDC**, the *Re*-face of the imine is available for the subsequent reaction with enamine **7a**, in *Si*-**cDC** is the *Si*-face. In both cases, the imine is stabilized by hydrogen bonding interactions through a bifunctional activation mode and by parallel-displacement (PD)–π-stacking interactions. Binary complex *Si*-**cDC** was found to be 2.7 kcal mol^−1^ more stable than *Re*-**cDC**. The greater relative stabilization of *Si*-**cDC** is likely due to the more effective overlap of the anthracenyl π-orbitals of the catalyst and the aryl fragment of the imine (PD-type; blue and green strands in Fig. 1). As shown, the distance between the 3,5-bis(trifluoromethyl)phenyl and the fused benzene rings of the anthracenyl group is shorter for *Si*-**cDC** relative to *Re*-**cDC** (3.68 and 3.81 *vs.* 3.95 Å, respectively). Additionally, the bis(trifluoromethyl)phenyl group of the imine appears to be conformationally restrained due to the hydrogen bonding interactions established between one of the hydrogen atoms at the *ortho*-position and one of the oxygen atoms of the phosphoric acid (with distances of 2.39 Å for *Si*-**cDC** and 2.44 Å for *Re*-**cDC**).

The coplanar orientation of the imine and the anthracenyl group, favoured by the PD–π interactions established between the latter and the 3,5-bis(trifluoromethyl)phenyl group, contributes to shield one of the enantiotopic faces of the imine, thus leaving the opposite one fully accessible for the reaction with enamine derivative **7a**.^13^ These non-covalent interactions were evaluated using the NCI-index based on electron density described by Yang and co-workers,^14^ and we found that they were particularly relevant for the binary complex *Si*-**cDC** (for details, see the ESI‡).

The particular geometry of the anthracenyl group with three rings arranged in a linear sequence appears to be critical to the stabilization of the binary complexes, and particularly of *Si*-**cDC**, by the above-mentioned stabilizing π-interactions. The extraordinary results we obtained with anthracenyl-substituted BINOL-derived phosphoric acid **CPAF** could be associated to this unique structural feature of the anthracenyl group.^15^ For the sake of comparison, we performed a similar analysis of the binary complexes derived from other chiral phosphoric acids with different aryl substituents, in particular the 2,4,6-triisopropylphenyl-substituted and the 9-phenanthryl-substituted BINOL-derived phosphoric acids (**CPAB** and **CPAD**, respectively). For these skeletons, the non-covalent interactions appear to be less important and the computed energy values predicted a poor to modest stereoselectivity for the formation of the final product, in agreement with our experimental results (for details, see the ESI‡).

**Fig. 2:**
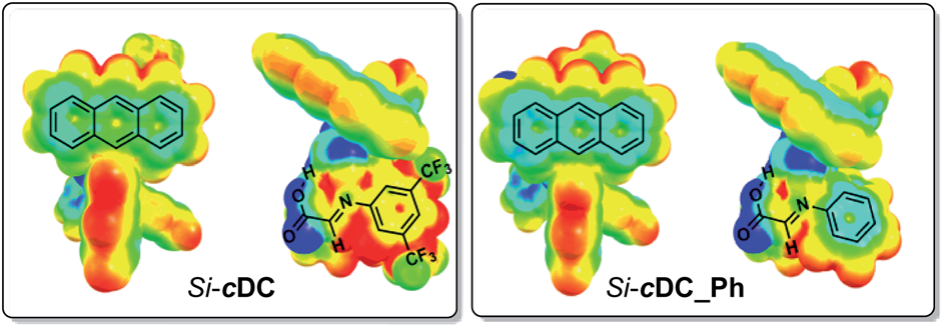
Representation of computed molecular electrostatic potential (MESP) of binary complex *Si*-**cDC** and *Si*-**cDC_Ph**. Colour codes: −0.04 a.u. (blue colour) to 0.04 a.u. (red colour) (computations at the wB97XD/DEF2SVPP(SMD,toluene) level).

We had experimentally found that electron-withdrawing groups at the aryl ring of the imine (*i.e.* at the starting aniline **3**) were required in order to obtain good results. This observation was computationally addressed by evaluation of the previously mentioned π-stacking interactions of the binary complexes as a function of the substituents at the imine aryl ring. In this regard, it has been previously suggested that the π-stacking interaction is enhanced when one of the aromatic rings contains electron-withdrawing substituents because the associated decrease of the electron density of the aromatic ring diminishes the electrostatic repulsion between the aryl groups.^16^ Our computations confirmed that the absence of the trifluoromethyl substituents led to a weakened π-interaction between the anthracenyl and the aryl group of the imine (for details, see ESI‡). Specifically, we evaluated the electron density distribution of *Si*-**cDC** (with a 3,5-bis(trifluoromethyl)phenyl group) in comparison with that of *Si*-**cDC_Ph** (with a phenyl group). This study allowed us to confirm the superior electronic complementarity when the 3,5-bis(trifluoromethyl)phenyl group was involved. This can be easily observed in the molecular electrostatic potential (MESP) representation shown in Fig. 2.^17^

**Fig. 3:**
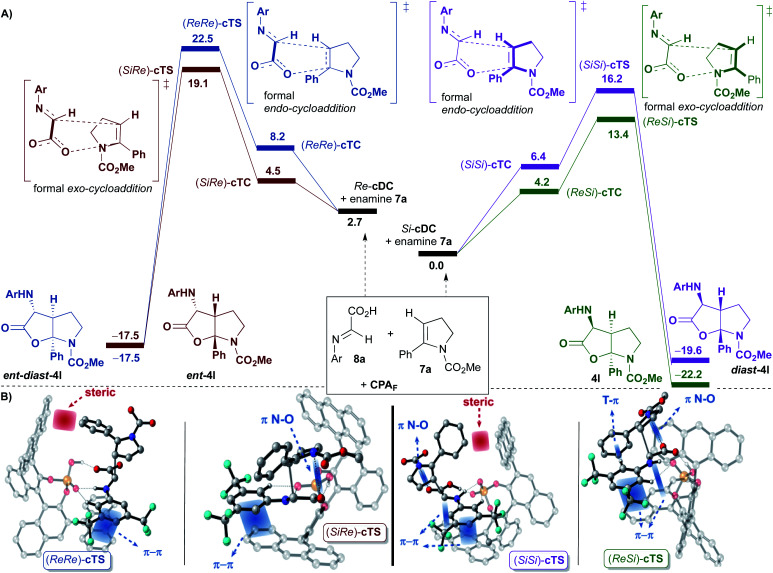
(A) Reaction free energy profiles (in kcal mol^−1^) and representation of transitions states for the concerted pathway of the [3 + 2]-cycloaddition (Ar = 3,5-(CF_3_)_2_–C_6_H_3_) catalysed by **CPAF** computed at the wB97XD/DEF2TZVPP(SMD,toluene)//wB97XD/DEF2SVPP(SMD,toluene) level. The *ReSi* (green), *SiRe* (red), *ReRe* (blue) and *SiSi* (purple) paths are shown. **CPAF** has been removed for clarity. (B) Representation of computed transition states for the four approaches (irrelevant hydrogen atoms have been removed for clarity).

Once the binary complexes formed by coordination of the imine **8a** to the 9-anthracenyl-substituted BINOL-derived phosphoric acid **CPAF** were analysed, we addressed their subsequent [3 + 2]-cycloaddition reactions with enamine derivative **7a** (Fig. 3). Since **7a** can react with binary complexes *Re*-**cDC** and *Si*-**cDC** through its *Re*- or *Si*-face, four possible ternary complexes (*Re*,*Re*-**cTC**, *Si*,*Si*-**cTC**, *Si*,*Re*-**cTC** and *Re*,*Si*-**cTC**) were characterized. Considering our previous studies with the achiral dimethyl hydrogen phosphate (**DMP**; see Scheme 5), we expected to be able to analyse the evolution of these intermediates through the two possible mechanistic options, namely the concerted and the stepwise. However, despite having characterized several transition states for the first bond formation of the stepwise mechanism, both the size and the structural rigidity of the chiral pocket of phosphoric acid **CPAF** hindered the conformational changes required for the formation of the C–O bond in the second step of the process. Thus, the stepwise pathway was discarded for this complex system, and we focused on the concerted mechanism.

For this mechanism, two transition states for each of the four facial approaches (*ReRe*, *ReSi*, *SiRe* and *SiSi*), which differ by the conformation of the carbamate (*s-cis* or *s-trans*), were characterized. Although we were able to locate all the transition states, only the results with the more stable conformer will be shown (*i.e. Re*,*Re*-**cTS**, *Si*,*Si*-**cTS**, *Si*,*Re*-**cTS** and *Re*,*Si*-**cTS**; Fig. 3).

Interestingly, the approaches of **7a** to the most stable binary complex *Si*-**cDC** leading to **4l** or **diast-4l** were favoured over the alternative pathways involving *Re*-**cDC** (Fig. 3). Thus, the (*ReSi*)-**cTS** and (*SiSi*)-**cTS** (13.4 and 16.2 kcal mol^−1^, respectively) were found to be highly favoured when compared to the diastereoisomeric (*SiRe*)-**cTS** and (*ReRe*)-**cTS** transition states (19.1 and 22.5 kcal mol^−1^, respectively). The strongest π-interactions between one of the anthracenyl groups and the aryl group of imine **8** in (*ReSi*)-**cTS** and (*SiSi*)-**cTS** when compared to the same interaction in (*SiRe*)-**cTS** and (*ReRe*)-**cTS** seems to justify the preferential pathways leading to **4l** and **diast-4l**. In other words, it seems that the highly stabilizing π-interactions we had observed in the binary complex *Si*-**cDC**, structurally associated to the particular geometry of the anthracenyl group, are maintained along the course of the reaction, contributing to further stabilize the corresponding transition states (*ReSi*)-**cTS** and (*SiSi*)-**cTS**.

As in the achiral model system, the global processes shown in Fig. 3A are highly exergonic, being the reaction energy values of −17.5, −19.6, −17.5 and −22.2 kcal mol^−1^ for **ent-diast-4l**, **diast-4l**, **ent-4l** and **4l**, respectively. From the transition state energies computed for this multicomponent reaction, the Curtin–Hammett principle predicts that the expected diastereomeric ratio **4l**/**diast-4l** would be *ca.* 99 : 1, and the enantiomeric ratio **4l**/**ent-4l** would be >99 : 1, which is in excellent agreement with the experimental results.

In our reaction, the diastereoselectivity (formation of **4l** or **ent-4l***versus***diast-4l** or **ent-diast-4l**) is determined by the orientation of the enamine **7a** upon reaction with the binary complexes *Si*-**cDC** or *Re*-**cDC** (*endo* or *exo* approaches). As shown, transition states (*SiSi*)-**cTS** and (*ReRe*)-**cTS**, in which the phenyl group of enamine **7a** is oriented towards the catalytic pocket (a formal *endo*-cycloaddition), are destabilized relative to the (*ReSi*)-**cTS** and (*SiRe*)-**cTS** (a formal *exo*-cycloaddition) due to severe steric interactions between the phenyl substituent of the enamine **7a** and one of the anthracenyl groups of the catalyst. Thus, it seems that the steric factors play an important role in determining the diastereoselectivity of the reaction.

At this point, the activation strain model was used to provide further semi-quantitative insight into the stereoinduction and the computed energies are shown in Table 2.^18^ These results provide additional support to the assumption that diastereoselectivity is determined by the distortion in the transition state, favoring those that show lower values. This is reflected in ΔΔ*E*_strain_ for (*ReRe*)-**cTS** and (*SiSi*)-**cTS**, namely 2.92 and 1.76 kcal mol^−1^, respectively, which is related to the above-mentioned steric interactions between the phenyl group of **7a** and the anthracenyl fragment.

**Table 2:** Activation strain model for the transition states of the concerted pathway of the [3 + 2]-cycloaddition catalyzed by **CPAF** computed at the ωB97XD/def2TZVPP level (kcal mol^−1^)

	ΔΔ*E*^‡^^a^	ΔΔ*E*_strain_^b^	ΔΔ*E*_int_^c^
(*Re*,*Re*)-**cTS**	9.51	2.92	2.34
(*Si*,*Re*)-**cTS**	6.33	0.64	1.45
(*Si*,*Si*)-**cTS**	2.62	1.76	0.87
(*Re*,*Si*)-**cTS**	0.00	0.00	0.00

a ΔΔ*E*^‡^ is the activation energy relative to the lower energy transition state (*Re*,*Si*)-**cTS**.

b ΔΔ*E*_strain_ is the relative total distortion energy of the transition states.

c ΔΔ*E*_int_ is the relative interaction energy of the transition states calculated with the corrected energies.

The enantioselectivity of the process (formation of **4l***versus***ent-4l**, or of **diast-4l***versus***ent-diast-4l**), appears to mainly be determined by electronic factors. Thus, (*ReSi*)-**cTS** (13.4 kcal mol^−1^) leading to **4l** is favoured over (*SiRe*)-**cTS** (19.1 kcal mol^−1^) affording **ent-4l**) due to the stronger π-interactions noticed in the former and being established between one of the anthracenyl groups and the 3,5-bis(trifluoromethyl)phenyl group of the imine **8** (see Fig. 3). The same trend can be noticed when comparing (*SiSi*)-**cTS** (16.2 kcal mol^−1^) leading to **diast-4l** with (*ReRe*)-**cTS** (22.5 kcal mol^−1^) generating **ent-diast-4l** (see Fig. 3). As previously noted, those strong π-interactions can ultimately be associated to the particular linear geometry of the anthracenyl group.

Finally, some other particular features of transition state (*ReSi*)-**cTS** that leads to **4l** should be remarked. In addition to the previously commented strong π-interaction between one of the anthracenyl groups and the 3,5-bis(trifluoromethyl)phenyl group of the imine **8**, another strong T-shaped edge-to-face C_Ar_H–π interaction between the 3,5-bis(trifluoromethyl)phenyl group of imine **8** and the phenyl group of the enamine **7a** was observed (see Fig. 3B). This is in agreement with our experimental observation that showed that an aryl substituent at the terminal position of the alkyne of amine **1** (eventually, an aryl substituent in enamine **7**) was required in order to get good results.^9^

**Fig. 4:**
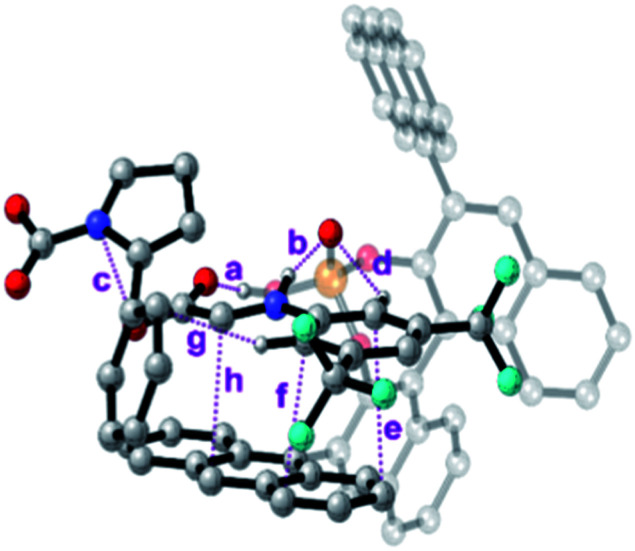
Bond critical points for the most important non-covalent interactions characterized (QTAIM) on the (*Re*,*Si*)-**cTS**.

To further analyse all these non-covalent interactions, we studied the topological distribution of electron density in the optimized geometries of the four transition states using QTAIM (see the ESI‡).^19^ For the transition state of lower activation energy, namely (*Re*,*Si*)-**cTS**, two additional interactions between **7a** and *Si*-**cDC** (c and g, Fig. 4) could be identified along with those already noted before (a, b and d–f, Fig. 4). In particular, the trifluoromethyl substituent favors the H_ortho_–π interactions. The *ρ*_BCP_ for π–π and H–X (X is N or O) interactions were found to be in the range of 0.014–0.005 au and 0.093–0.046 au respectively. For the lowest energy transition state, the π–π interaction between the imine and the anthracenyl fragment becomes 0.006 au. This is consistent with the lower ΔΔ*E*_int_ predicted for this transition state using the activation strain model (Table 2). In contrast, the lower number of non-covalent interactions predicted by QTAIM for the less favored transition state (*Re*,*Re*)-**cTS** (see the ESI‡), translates into higher ΔΔ*E*_int_ (2.34 kcal mol^−1^; Table 2).

Overall, the computational study indicates that the preferential formation of compounds **4** over the alternative isomers (**ent-4**, **diast-4** and **ent-diast-4**) appears to be a summation of key factors related to the specific structure of the anthracenyl-substituted BINOL-derived phosphoric acid catalyst **CPAF** and the reagents. Thus, the particular geometry of the anthracenyl group of the catalyst along with the presence of electron-withdrawing groups in the starting aniline allows the optimization of parallel-displacement π-interactions. This stabilization is less important with alternative enantiopure phosphoric acids having a shorter bicyclic aromatic ring (*i.e.*, naphthyl) or *ortho*-fused (*i.e.* phenanthryl) or analogues with other substituents (*i.e.*, 2,4,6-triisopropylphenyl or triphenylsilane). The rigidity and steric hindrance of the anthracenyl groups of the catalyst likely contributes to the diastereoselectivity of the process, since it determines the size of the catalytic pocket. In this regard, the steric interactions between the aryl group of enamine **7** when approaching the binary complex formed between the catalyst and the imine **8** seems to be determinant for the diastereoselectivity of the reaction. Finally, the excellent enantioselectivity of our process appears to be associated to the strong π-interactions established between the aryl group of imine **8** with one of the anthracenyl substituents of the **CPAF** catalyst and also with the aryl substituent of enamine **7**.

## Conclusions

We have developed a distereo and enantioselective synthesis of furo[2,3-*b*]pyrrole derivatives by means of a multicomponent and multicatalytic process. Thus, the reaction of a 3-butynamine derivative with glyoxylic acid and an aniline derivative in the presence of a dual catalytic system, formed by a gold complex and chiral BINOL-derived phosphoric acid, led to the formation of the corresponding furo[2,3-*b*]pyrrole derivative in very high yield and stereoselectivity. These products, containing three contiguous stereocenters, were selectively obtained only when the BINOL-derived phosphoric acid catalyst was substituted at 3- and 3′-positions with anthracenyl groups (**CPAF**). The extraordinary and unique performance of this catalyst was computationally studied in order to further understand the mode of action of chiral phosphoric acids in asymmetric processes. This study further supports the crucial influence of non-covalent interactions, established between the chiral phosphoric acid and the reagents, on the control of the stereoselectivity of asymmetric reactions. These interactions seem to be at least as important as the usually referred steric effects. In the reaction here described, features intimately related to those non-covalent interactions such as the linear geometry of the anthracenyl group of the catalyst, the electron density of the aniline, the electronic complementarity of aromatic rings involved in van der Waals interactions or the presence of an aryl substituent in the starting 3-butynamine derivative seem to be essential in order to justify the excellent results in terms of yield, diastereo and enantioselectivity experimentally observed. The work here presented not only demonstrates the power of asymmetric multicomponent and multicatalytic reactions to transform simple materials into complex products, but also provides new vistas to better understand how chiral phosphoric acids operate in asymmetric processes.

## Conflicts of interest

There are no conflicts to declare.

## Supplementary Material

SC-011-D0SC03342A-s001

SC-011-D0SC03342A-s002
