# Supplementary material for: Multicomponent and multicatalytic asymmetric synthesis of furo[2,3-b]pyrrole derivatives: further insights into the mode of action of chiral phosphoric acid catalysts
Source: Chem Sci. 2020 Aug 12;11(34):9181–90. doi: 10.1039/d0sc03342a (PMC8161233; doi:10.1039/d0sc03342a)
Supplement: SC-011-D0SC03342A-s001 [file SC-011-D0SC03342A-s001.pdf]

Electronic Supplementary Material (ESI) for Chemical Science.
This journal is © The Royal Society of Chemistry 2020

Supplementary Information for

Multicomponent and multicatalytic asymmetric synthesis of furo[2,3-*b*]pyrrole derivatives: Further insights into the mode of action of chiral phosphoric acid catalysts

Lara Cala, Pedro Villar, Ángel R. de Lera, Francisco J. Fañanás, Rosana Álvarez,* and Félix Rodríguez*

Contents:

		Pag
1.	General Information	2
2.	Experimental Procedures and Characterization Data	3
3.	NMR Spectra and HPLC Chromatograms	11
4.	X-Ray	39
5.	Computational Details	40
5.1.	Computational Methods	40
5.2.	Concerted Mechanism with Achiral Catalyst	41
5.3.	Stepwise Mechanism with Achiral Catalyst	42
5.4.	Concerted Mechanism with Chiral Catalyst	45
5.5	Activation Strain Model	49
5.6	QTAIM analysis	50
5.7	XYZ Coordinates	54

1. General Information

^1H NMR spectra were recorded on a Bruker AMX-400 (400 MHz), Bruker AV-300 (300 MHz) or Bruker DPX-300 (300 MHz). Chemical shifts are reported in ppm from tetramethylsilane with the residual solvent resonance as the internal standard ($CDCl_3$: δ= 7.26 ppm; CD_3CN: δ= 1.94 ppm). Data are reported as follows: chemical shift, multiplicity (s: singlet, d: doublet, dd: double doublet, ddd: double doublet of doublets, dtd: double triplet of doublets, td: triplet of doublets, t: triplet, q: quartet, m: multiplet), coupling constants (J in Hz), integration and assignment. ^{13}C NMR spectra were recorded on a Bruker AMX-400 (100 MHz), Bruker AV-300 (75 MHz) or Bruker DPX-300 (75 MHz) with complete proton decoupling. Chemical shifts are reported in ppm from tetramethylsilane with the solvent resonance as internal standard ($CDCl_3$: δ = 77.2 ppm; C_6D_6 = 128.1 ppm; CD_3CN: δ = 118.3 ppm). Bidimensional NMR experiments (COSY, HSQC, HMBC and NOESY) were recorded on a Bruker AV-300 (300 MHz). High-resolution mass spectrometry was carried out on a Finnigan-Mat 95 spectrometer. Solvents were dried with a PureSolv® column system before use. Starting materials were commercially acquired or prepared according to the methods reported in the literature.[1]

[1] a) V. Belting, N. Krause, *Org. Lett.*, 2006, **8**, 4489. b) J. Barluenga, A. Mendoza, F. Rodríguez, F. J. Fañanás, *Angew. Chem. Int. Ed.*, 2009, **48**, 1644. c) S. Schulz, S. Yildizhan, K. Stritzke, C. Estrada, L. E. Gilbert, *Org. Biomol. Chem.*, 2007, **5**, 3434. d) J. R. Dunetz, R. L. Danheiser, *J. Am. Chem. Soc.*, 2005, **127**, 5776. e) S. A. Hashmi, K. S. Pradipta *Adv. Synth. Catal.*, 2004, **346**, 432. f) D. MacLeod, D. Moorcroft, P. Quayle, M. R. J. Dorrity, J. F. Malone, G. M. Davies, *Tetrahedron Lett.*, 1990, **31**, 6077. g) P. Le Ménez, J.-D. Brion, N. Lensen, E. Chelain, A. Pancrazi, J. Ardisson, *Synthesis*, 2003, 2530. h) K. Miura, D. Wang, Y. Matsumoto, A. Hosomi, *Org. Lett.*, 2005, **7**, 503.

2. Experimental Procedures and Characterization Data

Synthesis of Furo[2,3-*b*]pyrrole Derivatives (+)-4

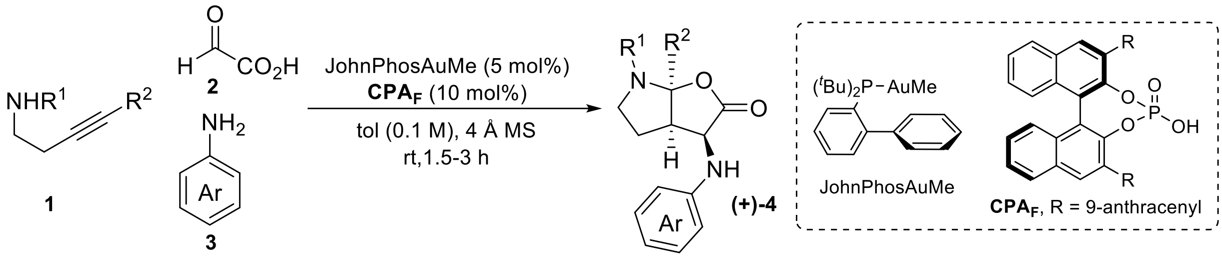

A carousel tube with a magnetic stirring bar was charged under an atmosphere of argon with activated 4 Å molecular sieves (100 mg/mL), (*R*)-3,3′-bis(9-anthracenyl)-1,1′-binaphthyl-2,2′-diyl hydrogenphosphate **CPA$_F$** (10 mol%), methyl[(1,1′-biphenyl-2-yl)di-tert-butyl phosphine]gold(I) (5 mol%) and dry toluene (0.1 M). The mixture was stirred at room temperature for 30 minutes and then, glyoxylic acid **2** (1.6 equiv.) and the corresponding aniline **3** (1.2 equiv.) were added. After 10 minutes at room temperature, the corresponding 3-butyn-1-ylcarbamate derivative **1** (1 equiv.) was added. The reaction was allowed to react for 1.5 – 3h and then the mixture was filtered through a short pad of silica gel and Celite® with a 1:1 mixture of hexanes and ethyl acetate. The solvent was removed under reduced pressure and the residue was purified by flash column chromatography on silica gel to afford the corresponding pure compound **(+)-4**.

tert-Butyl (3*S*,3a*R*,6a*R*)-6a-(4-chlorophenyl)-3-[(3-nitrophenyl)amino]-2-oxohexahydro-6*H*-furo[2,3-*b*]pyrrole-6-carboxylate [(+)-4a]

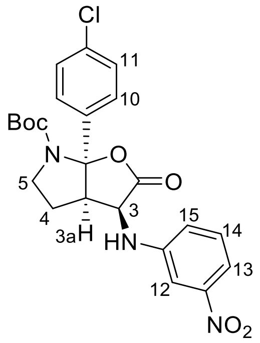

Yellow solid. R$_f$ = 0.35 (silica gel, hexanes:EtOAc 2:1). **^{1}H NMR** (400 MHz, CDCl$_3$) δ 7.65 (dd, *J* = 8.1, 2.1 Hz, 1H, H$_{13}$), 7.47 – 7.42 (m, 2H, H$_{11}$), 7.35 – 7.32 (m, 3H, H$_{10}$ and H$_{14}$), 7.31 (t, *J* = 2.1 Hz, 1H, H$_{12}$), 6.89 (dd, *J* = 7.8, 2.1 Hz, 1H, H$_{15}$), 4.70 (d, *J* = 2.8 Hz, 1H, N*H*), 4.25 (dd, *J* = 7.1, 2.8 Hz, 1H, H$_3$), 3.96 (bt, *J* = 10.1 Hz, 1H,H$_{5a}$), 3.78 (td, *J* = 10.1, 7.1 Hz, 1H, H$_{5b}$), 3.49 (bdt, *J* = 10.1, 7.1 Hz, 1H, H$_{3a}$), 2.07 – 1.97 (m, 1H, H$_{4a}$), 1.97 – 1.87 (m, 1H, H$_{4b}$), 1.60 (bs, 9H, Boc). **^{13}C NMR** (75 MHz, CDCl3) δ 173.4, 152.5, 149.5, 146.9, 134.9, 130.3, 129.3, 126.1, 119.6, 114.2, 106.9,

100.5, 81.9, 55.7, 54.6, 48.4, 28.0, 22.9. **HRMS** (APCI) $C_{23}H_{24}ClN_3O_6$ M^+ calcd. 473.1348, found 473.1347.

$[\alpha]_D^{28}$= +49° (c= 0.1, EtOAc). *er* = 99:1 [by HPLC in comparison with the racemate; Daicel CHIRALPAK AD-H, hexanes:^{i}PrOH 80:20, 0.6 ml/min, 226.9 nm, t_R (major)= 33 min, t_R (minor)= 42 min].

tert-Butyl (3*S*,3a*R*,6a*R*)-3-{[3,5-bis(trifluoromethyl)phenyl]amino}-6a-(4-chlorophenyl)-2-oxo hexahydro-6*H*-furo[2,3-*b*]pyrrole-6-carboxylate [(+)-4b]

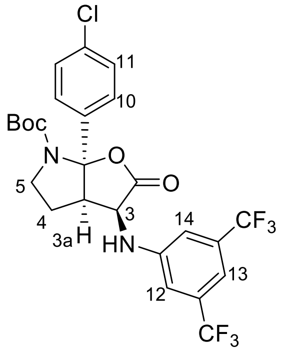

White solid. R_f = 0.31 (silica gel, hexanes:EtOAc 3:1). **^{1}H NMR** (400 MHz, $CDCl_3$) δ 7.48 - 7.43 (m, 2H, H_{11}), 7.36 - 7.32 (m, 2H, H_{10}), 7.28 (bs, 1H, H_{13}), 6.90 (bs, 2H, H_{12} and H_{14}), 4.81 (d, *J* = 2.7 Hz, 1H, N*H*), 4.24 (dd, *J* = 7.0, 2.7 Hz, 1H, H_3), 3.96 (bt, *J* = 10.9 Hz, 1H, H_{5a}), 3.79 (td, *J* = 10.9, 6.8 Hz, 1H, H_{5b}), 3.42 (bdt, *J* = 10.0, 7.0 Hz, 1H, H_{3a}), 2.05 – 1.96 (m, 1H, H_{4a}), 1.96 – 1.84 (m, 1H, H_{4b}), 1.16 (bs, 9H, Boc). **^{13}C NMR** (75 MHz, $CDCl_3$) δ 173.1, 152.4, 146.9, 134.9, 133.1 (q, J_{CF} = 33.5Hz), 129.3, 126.2, 123.3 (q, J_{CF} = 272.8 Hz), 112.6, 100.5, 81.9, 55.6, 54.5, 48.5, 27.9, 22.9. **^{19}F NMR** (282 MHz, $CDCl_3$) δ –63.2. **HRMS** (APCI) $C_{25}H_{23}ClF_6N_2O_4$ M^+ calcd. 563.1167, found 563.1159.

$[\alpha]_D^{28}$= +63° (c= 0.1, EtOAc). *er* = 95:5 [by HPLC in comparison with the racemate; Daicel CHIRALPAK AD-H, hexanes:^{i}PrOH 95:5, 0.3 ml/min, 251.6 nm, t_R (major)= 27 min, t_R (minor)= 49 min].

tert-Butyl (3*S*,3a*R*,6a*R*)-6a-(4-chlorophenyl)-3-[(2-fluoro-5-nitrophenyl)amino]-2-oxohexa hydro-6*H*-furo[2,3-*b*]pyrrole-6-carboxylate [(+)-4c]

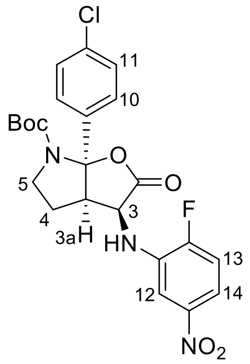

Light yellow solid. R_f = 0.36 (silica gel, hexanes:EtOAc 4:1). **^{1}H NMR** (300 MHz, $CDCl_3$) δ 7.68 (ddd, J_{HH} = 8.9, 2.7 Hz, J_{HF} = 4.2 Hz, 1H, H_{14}), 7.49 – 7.40 (m, 2H, H_{11}), 7.37 – 7.32 (m, 2H, H_{10}), 7.30 (dd, J_{HH} = 2.7 Hz, J_{HF} = 7.5 Hz, 1H, H_{12}), 7.15 (dd, J_{HH} = 8.9 Hz, J_{HF} = 10.2 Hz, 1H, H_{13}), 4.86 (t, J = 3.1 Hz, 1H, NH), 4.28 (dd, J = 7.3, 3.1 Hz, 1H, H_3), 4.00 (ddd, J = 10.3, 8.5, 1.1 Hz, 1H, H_{5a}), 3.80 (td, J = 10.3, 6.6 Hz, 1H, H_{5b}), 3.52 (dt, J = 10.8, 7.3 Hz, 1H, H_{3a}), 2.10 – 1.85 (m, 2H, H_4), 1.22 (bs, 9H, Boc). **^{13}C NMR** (75 MHz, $CDCl_3$) δ 172.9, 154.9 (d, J_{CF} = 253.0 Hz), 152.4, 145.0, 135.5 (d, J_{CF} = 13.2 Hz), 134.9, 129.3 (J_{CF} = 41.3 Hz), 129.31, 126.2, 115.5 (d, J_{CF} = 21.2 Hz), 115.0 (d, J_{CF} = 8.5 Hz), 107.0 (d, J_{CF} = 4.8 Hz), 100.5, 81.9, 55.4, 54.3, 48.4, 27.97, 22.9. **^{19}F NMR** (282 MHz, $CDCl_3$) δ –121.4. **HRMS** (APCI) $C_{23}H_{23}ClFN_3O_6$ M^+, calcd. 491.1254, found 491.1257.

$[\alpha]_D^{28}$= +57° (c= 0.1, EtOAc). *er* = 92:8 [by HPLC in comparison with the racemate; Daicel CHIRALPAK AD-H, hexanes:^{i}PrOH 80:20, 0.6 ml/min, 225.7 nm, t_R (major)= 16 min, t_R (minor)= 37 min].

tert-Butyl (3*S*,3a*R*,6a*R*)-3-[(3-nitrophenyl)amino]-2-oxo-6a-phenylhexahydro-6*H*-furo[2,3-*b*] pyrrole-6-carboxylate [(+)-4d]

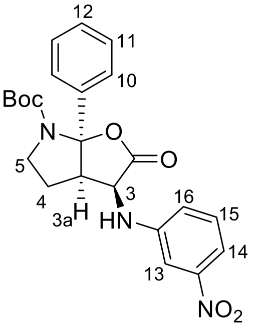

Yellow solid. R_f = 0.23 (silica gel, hexanes:EtOAc 2:1). **^{1}H NMR** (400 MHz, $CDCl_3$) δ 7.62 (dd, J = 8.1, 2.1 Hz, 1H, H_{14}), 7.48 – 7.41 (m, 3H, H_{11} and H_{12}), 7.40 – 7.35 (m, 2H, H_{10}), 7.32 (d, J = 2.3 Hz, 1H, H_{13}), 7.31 (t, J = 8.1 Hz, 1H, H_{15}), 6.88 (dd, J = 8.1, 2.1, Hz, 1H, H_{16}), 4.74 (d, J = 2.6 Hz, 1H, NH), 4.28 (dd, J = 7.3, 2.6 Hz, 1H, H_3), 3.96 (ddd, J = 10.9, 8.7, 1.6 Hz, 1H, H_{5a}), 3.80 (td, J = 10.9, 6.7 Hz, 1H, H_{5b}), 3.52 (dt, J = 10.9, 7.7 Hz, 1H, H_{3a}), 2.00 (dtd, J = 13.4, 7.1, 1.6 Hz, 1H, H_{4a}), 1.94 (dtd, J = 13.4, 10.9, 8.7 Hz, 1H, H_{4b}), 1.10 (bs, 9H, Boc). **^{13}C NMR** (75 MHz, $CDCl_3$) δ 173.7, 152.7, 149.5, 147.1, 130.3, 129.0, 128.8, 124.6, 119.5, 114.0, 106.9, 100.9, 81.6, 55.8, 54.7, 48.5, 27.9, 22.9. **HRMS** (APCI) $C_{23}H_{25}N_3O_6$ M^+ calcd. 438.1660, found 438.1671.

$[\alpha]_D^{28}$= +41° (c= 0.1, EtOAc). *er* = 98:2 [by HPLC in comparison with the racemate; Daicel CHIRALPAK OD-H, hexanes:^{i}PrOH 90:10, 0.5 ml/min, 235.5 nm, t_R (major)= 48 min, t_R (minor)= 75 min].

***tert*-Butyl (3*S*,3a*R*,6a*R*)-3-{[3,5-bis(trifluoromethyl)phenyl]amino}-2-oxo-6a-phenylhexahydro -6*H*-furo[2,3-*b*]pyrrole-6-carboxylate [(+)-4e]**

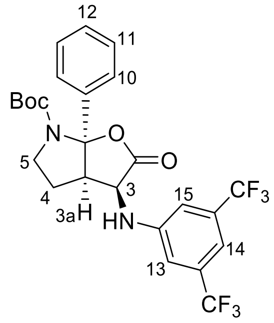

White solid. R_f = 0.32 (silica gel, hexanes:EtOAc 4:1). **^{1}H NMR** (400 MHz, $CDCl_3$) δ 7.50 – 7.42 (m, 3H, H_{11} and H_{12}), 7.41 – 7.36 (m, 2H, H_{10}), 7.26 (bs, 1H, H_{13}), 6.90 (bs, 2H, H_{14} and H_{15}), 4.84 (d, *J* = 3.2 Hz, 1H, N*H*), 4.28 (dd, *J* = 7.8, 3.2 Hz, 1H, H_3), 3.97 (ddd, *J* = 10.9, 8.7, 1.6 Hz, 1H, H_{5a}), 3.81 (td, *J* = 10.9, 6.7 Hz, 1H, H_{5b}), 3.46 (dt, *J* = 10.9, 7.8 Hz, 1H, H_{3a}), 2.02 (dtd, *J* = 13.4, 6.6, 1.8 Hz, 1H, H_{4a}), 1.94 (dtd, *J* = 13.4, 10.9, 8.7 Hz, 1H, H_{4b}), 1.11 (bs, 9H, Boc).**^{13}C NMR** (75 MHz, $CDCl_3$) δ 173.5, 152.7, 147.0, 140.3, 132.9 (q, J_{CF} = 33.0 Hz), 129.1, 128.9, 124.6, 123.3 (q, J_{CF} = 272.9 Hz), 112.5, 101.0, 81.6, 55.6, 54.6, 48.4, 27.9, 22.97. **^{19}F NMR** (282 MHz, $CDCl_3$) δ –63.1. **HRMS** (APCI) $C_{25}H_{24}F_6N_2O_4$ M^+ calcd. 529.1557, found 529.1549.

$[\alpha]_D^{28}$= +57° (c= 0.1, EtOAc). *er* = 99:1 [by HPLC in comparison with the racemate; Daicel CHIRALPAK AD-H, hexanes:^{i}PrOH 97:3, 0.2 ml/min, 251.6 nm, t_R (major)= 80 min, t_R (minor)= 100 min].

***tert*-Butyl (3*S*,3a*R*,6a*R*)-6a-(4-methoxyphenyl)-3-[(3-nitrophenyl)amino]-2-oxo hexahydro-6*H*-furo[2,3-*b*]pyrrole-6-carboxylate [(+)-4f]**

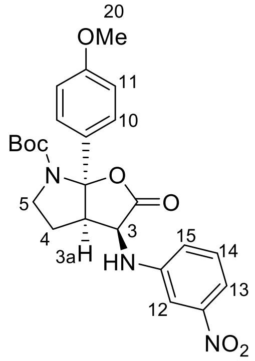

Yellow solid. R_f = 0.14 (silica gel, hexanes:EtOAc 1:1). **^{1}H NMR** (400 MHz, $CDCl_3$) δ 7.61 (dd, *J* = 8.1, 2.1 Hz, 1H, H_{13}), 7.33 – 7.27 (m, 4H, H_{10}, H_{13} and H_{14}), 6.98 – 6.93 (m, 2H, H_{11}), 6.89 (dd, *J* =

8.1, 2.1 Hz, 1H, H_{15}), 4.77 (s, 1H, N*H*), 4.28 (d, *J* = 7.7 Hz, 1H, H3), 3.94 (ddd, *J* = 11.0, 8.6, 1.1 Hz, 1H, H_{5a}), 3.85 (s, 3H, H_{20}), 3.77 (td, *J* = 11.0, 6.7 Hz, 1H, H_{5b}), 3.48 (dt, *J* = 11.1, 7.7 Hz, 1H, H_{3a}), 2.03 – 1.94 (m, 1H, H_{4a}), 1.93 – 1.85 (m, 1H, H_{4b}), 1.15 (bs, 9H). **^{13}C NMR** (75 MHz, $CDCl_3$) δ 173.7, 159.9, 152.8, 149.5, 147.1, 130.2, 125.9, 119.5, 114.3, 113.9, 106.9, 101.0, 81.4, 55.8, 55.6, 54.6, 48.4, 27.9, 22.9. **HRMS** (APCI) $C_{24}H_{27}N_3O_7$ M^+ calcd. 468.1765, found 468.1760.

$[\alpha]_D^{28}$= +38° (c= 0.1, EtOAc). *er* = 99:1 [by HPLC in comparison with the racemate; Daicel CHIRALPAK AD-H, hexanes:^{i}PrOH 80:20, 0.6 ml/min, 230.4 nm, t_R (major)= 41 min, t_R (minor)= 64 min].

***tert*-Butyl (3*S*,3a*R*,6a*R*)-3-{[3,5-bis(trifluoromethyl)phenyl]amino}-6a-(4-methoxy phenyl)-2-oxohexahydro-6*H*-furo[2,3-*b*]pyrrole-6-carboxylate [(+)-4g]**

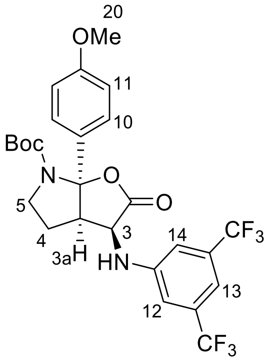

White solid. R_f = 0.36 (silica gel, hexanes:EtOAc 3:1). **^{1}H NMR** (400 MHz, $CDCl_3$) δ 7.31 – 7.28 (m, 2H, H_{10}), 7.26 (bs, 1H, H_{12}), 6.99 – 6.95 (m, 2H, H_{11}), 6.90 (bs, 2H, H_{13} and H_{14}), 4.81 (d, *J* = 3.1 Hz, 1H, N*H*), 4.26 (dd, *J* = 7.3, 3.1 Hz, 1H, H_3), 3.95 (bt, *J* = 10.9 Hz, 1H, H_{5a}), 3.87 (s, 3H, H_{20}), 3.78 (td, *J* = 10.9, 6.6 Hz, 1H, H_{5b}), 3.42 (dt, *J* = 11.1, 7.3 Hz, 1H, H_{3a}), 2.03 – 1.93 (m, 1H, H_{4a}), 1.93 – 1.82 (m, 1H, H_{4b}), 1.15 (bs, 9H, Boc). **^{13}C NMR** (75 MHz, $CDCl_3$) δ 173.5, 160.0, 152.7, 147.1, 132.9 (q, J_{CF} = 32.9 Hz), 125.95, 123.3 (q, J_{CF} = 272.5 Hz), 114.3, 114.1, 112.6, 101.1, 81.5, 55.7, 55.6, 54.6, 48.4, 27.99, 22.9. **^{19}F NMR** (282 MHz, $CDCl_3$) δ –63.1. **HRMS** (APCI) $C_{26}H_{26}F_6N_2O_5$ M^+ calcd. 559.1662, found 559.1670.

$[\alpha]_D^{28}$= +33° (c= 0.1, EtOAc). *er* = 99:1 [by HPLC in comparison with the racemate; Daicel CHIRALPAK AD-H, hexanes:^{i}PrOH 95:5, 0.3 ml/min, 251.6 nm, t_R (major)= 35 min, t_R (minor)= 80 min].

***tert*-Butyl (3*S*,3a*R*,6a*R*)-3-[(3-nitrophenyl)amino]-2-oxo-6a-(*p*-tolyl)hexahydro-6*H*-furo[2,3-*b*] pyrrole-6-carboxylate [(+)-4h]**

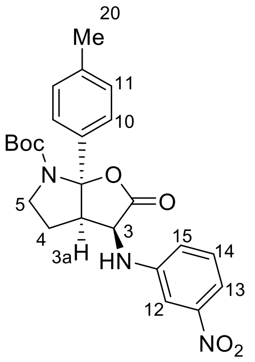

Yellow solid. R_f = 0.23 (silica gel, hexanes:EtOAc 2:1). **^{1}H NMR** (400 MHz, $CDCl_3$) δ 7.60 (dd, *J* = 8.1, 2.3 Hz, 1H, H_{13}), 7.31 (t, *J* = 2.3 Hz, 1H, H_{12}), 7.31 (t, *J* = 8.1 Hz, 1H, H_{14}), 7.25 (s, 4H, H_{10} and H_{11}), 6.88 (dd, *J* = 8.1, 2.3 Hz, 1H, H_{15}), 4.77 (d, *J* = 3.0 Hz, 1H, N*H*), 4.28 (dd, *J* = 7.6, 3.0 Hz, 1H, H_3), 3.94 (bt, *J* = 10.9 Hz, 1H, H_{5a}), 3.78 (td, *J* = 10.9, 6.7 Hz, 1H, H_{5b}), 3.49 (dt, *J* = 10.8, 7.6 Hz, 1H, H_{3a}), 2.41 (s, 3H, H_{20}), 2.03 – 1.94 (m, 1H, H_{4a}), 1.94 – 1.85 (m, 1H, H_{4b}), 1.15 (bs, 9H, Boc). **^{13}C NMR** (101 MHz, $CDCl_3$) δ 173.8, 152.7, 149.5, 147.2, 138.7, 130.2, 129.6, 124.5, 119.5, 113.9, 106.9, 101.1, 81.4, 55.8, 54.6, 48.4, 27.9, 22.9, 21.2. **HRMS** (APCI) $C_{24}H_{27}N_3O_6$ M^+ calcd. 452.1816, found 452.1810.

$[\alpha]_D^{28}$= +78° (c= 0.1, EtOAc). *er* = 99:1 [by HPLC in comparison with the racemate; Daicel CHIRALPAK AD-H, hexanes:^{i}PrOH 80:20, 0.6 ml/min, 226.9 nm, t_R (major)= 28 min, t_R (minor)= 42 min].

Methyl (3*S*,3a*R*,6a*R*)-3-[(3-nitrophenyl)amino]-2-oxo-6a-phenylhexahydro-6*H*-furo[2,3-*b*]pyrrole-6-carboxylate [(+)-4i]

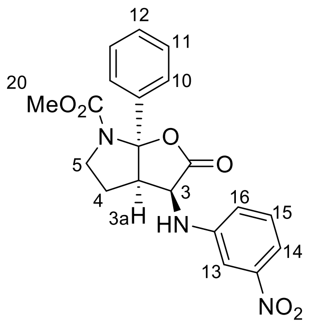

Yellow solid. R_f = 0.15 (silica gel, hexanes:EtOAc 1:1). **^{1}H NMR** (300 MHz, $CDCl_3$) δ 7.62 (dd, *J* = 8.1, 2.3 Hz, 1H, H_{14}), 7.51 – 7.37 (m, 5H, H_{10}-H_{12}), 7.32 (t, *J* = 2.3 Hz, 1H, H_{13}), 7.32 (t, *J* = 8.1 Hz, 1H, H_{15}), 6.90 (dd, *J* = 8.1, 2.3 Hz, 1H, H_{16}), 4.85 (d, *J* = 2.7 Hz, 1H, N*H*), 4.33 (dd, *J* = 7.8, 2.7 Hz, 1H, H_3), 4.02 (bt, *J* = 10.7 Hz, 1H, H_{5a}), 3.84 (td, *J* = 10.7, 6.9 Hz, 1H, H_{5b}), 3.66 (bs, 3H, H_{20}), 3.58 (td, *J* = 10.8, 7.8 Hz, 1H, H_{3a}), 2.10 – 1.93 (m, 2H, H_4). **^{13}C NMR** (75 MHz, $CDCl_3$) δ 173.4, 154.1, 149.4, 147.0, 139.4, 130.3, 129.1, 129.0, 124.5, 119.5, 113.9, 106.9, 100.9, 55.6, 54.2, 52.9, 48.8, 23.2. **HRMS** (APCI) $C_{20}H_{18}N_3O_6$ M^+ calcd. 396.1190, found 396.1195.

$[\alpha]_D^{28}$= +58° (c= 0.1, EtOAc). *er* = 98:2 [by HPLC in comparison with the racemate; Daicel CHIRALPAK AD-H, hexanes:^{i}PrOH 70:30, 0.6 ml/min, 236.3 nm, t_R (major)= 35 min, t_R (minor)= 71 min].

Methyl (3*S*,3a*R*,6a*R*)-3-{[2-fluoro-3-(trifluoromethyl)phenyl]amino}-2-oxo-6a-phenylhexahydro-6*H*-furo[2,3-*b*]pyrrole-6-carboxylate [(+)-4j]

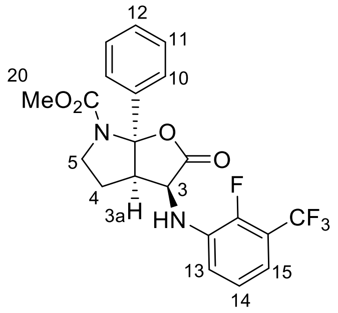

White solid. R_f = 0.37 (silica gel, hexanes:EtOAc 4:1). **^{1}H NMR** (400 MHz, $CDCl_3$) δ 7.48 – 7.37 (m, 5H, H_{10-12}), 7.02 (bt, *J* = 7.9 Hz, 1H, H_{13}), 6.99 – 6.95 (m, 1H, H_{15}), 6.65 (td, *J* = 7.9, 1.5 Hz, 1H, H_{14}), 4.76 (t, *J* = 2.8 Hz, 1H, N*H*), 4.27 (dd, *J* = 7.7, 2.8 Hz, 1H, H_3), 4.03 (bt, *J* = 10.8 Hz, 1H, H_{5a}), 3.84 (td, *J* = 10.8, 6.9 Hz, 1H, H_{5b}), 3.53 (bs, 3H, H_{20}), 3.48 (dt, *J* = 10.9, 7.7 Hz, 1H, H_{3a}), 2.11 – 2.04 (m, 1H, H_{4a}), 2.04 – 1.94 (m, 1H, H_{4b}). **^{13}C NMR** (75 MHz, $CDCl_3$) δ 173.0, 154.1, 148.6 (d, J_{CF} = 250.9 Hz), 139.5, 135.6 (d, J_{CF} = 10.6 Hz), 129.1, 128.96, 124.6, 124.5, 124.47, 120.9, 118.6 (d, J_{CF} = 32.8 Hz), 118.5 (d, J_{CF} = 32.7 Hz), 116.1 – 115.6 (m), 100.7, 55.6, 54.4, 52.9, 48.8, 23.3. **^{19}F NMR** (282 MHz, $CDCl_3$) δ −61.2 (d, J_{FF} = 13.1 Hz), −136.6 (q, J_{FF} = 13.1 Hz). **HRMS** (APCI) $C_{21}H_{17}F_4N_2O_4$ M^+ calcd. 437.1119, found 437.1122.

$[\alpha]_D^{28}$= +76° (c= 0.1, EtOAc). *er* = 96:4 [by HPLC in comparison with the racemate; Daicel CHIRALPAK AD-H, hexanes:^{i}PrOH 80:20, 0.6 ml/min, 239.8 nm, t_R (major)= 18 min, t_R (minor)= 28 min].

Methyl (3*S*,3a*R*,6a*R*)-3-[(2-fluoro-5-nitrophenyl)amino]-2-oxo-6a-phenylhexahydro-6*H*-furo[2,3-*b*]pyrrole-6-carboxylate [(+)-4k]

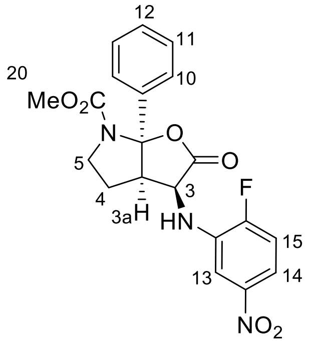

Light yellow solid. R_f = 0.24 (silica gel, hexanes:EtOAc 2:1). **^{1}H NMR** (401 MHz, $CDCl_3$) δ 7.67 (ddd, J_{HH} = 8.9, 2.6 Hz, J_{HF} = 4.2 Hz, 1H, H_{14}), 7.50 – 7.37 (m, 5H, H_{10}-H_{12}), 7.29 (dd, J_{HH} = 2.6 Hz,

J_{HF} = 7.4 Hz, 1H, H_{13}), 7.14 (dd, J_{HH} = 8.9 Hz, J_{HF} = 10.2 Hz, 1H, H_{15}), 4.86 (t, *J* = 3.3 Hz, 1H, N*H*), 4.31 (dd, *J* = 7.4, 3.3 Hz, 1H, H_3), 4.03 (bt, *J* = 10.8 Hz, 1H, H_{5a}), 3.85 (td, *J* = 10.8, 6.8 Hz, 1H, H_{5b}), 3.59 (td, *J* = 10.9, 7.4 Hz, 1H, H_{3a}), 3.50 (bs, 3H, H_{20}), 2.10 – 1.92 (m, 2H, H_4). **^{13}C NMR** (75 MHz, $CDCl_3$) δ 172.8, 154.9 (d, J_{CF} = 253.0 Hz), 153.2, 145.0, 135.5 (d, *J* = 13.3 Hz), 129.1, 128.9 (J_{CF} = 41.2 Hz), 124.4, 115.2 (d, J_{CF} = 21.2 Hz), 114.9 (d, J_{CF} = 8.7 Hz), 107.0, 55.3, 52.9, 48.7, 23.2. **^{19}F NMR** (282 MHz, $CDCl_3$) δ –123.5. **HRMS** (APCI) $C_{20}H_{17}FN_3O_6$ M^+ calcd. 414.1096, found 414.1098.

$[\alpha]_D^{28}$= +36° (c= 0.1, EtOAc). *er* = 91:9 [by HPLC in comparison with the racemate; Daicel CHIRALPAK AD-H, hexanes:^{i}PrOH 70:30, 0.6 ml/min, 243.3 nm, t_R (major)= 33 min, t_R (minor)= 55 min].

3. NMR Spectra and HPLC Chromatograms

***tert*-Butyl (3*S*,3a*R*,6a*R*)-6a-(4-chlorophenyl)-3-[(3-nitrophenyl)amino]-2-oxohexahydro-6*H*-furo[2,3-*b*]pyrrole-6-carboxylate [(+)-4a]**

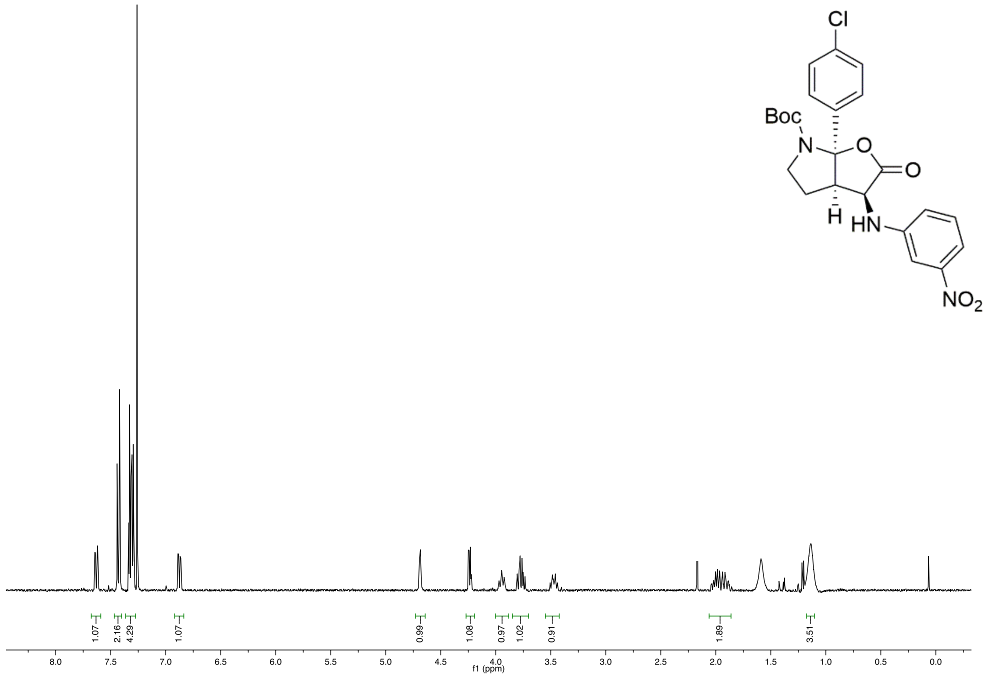

^{1}H-NMR

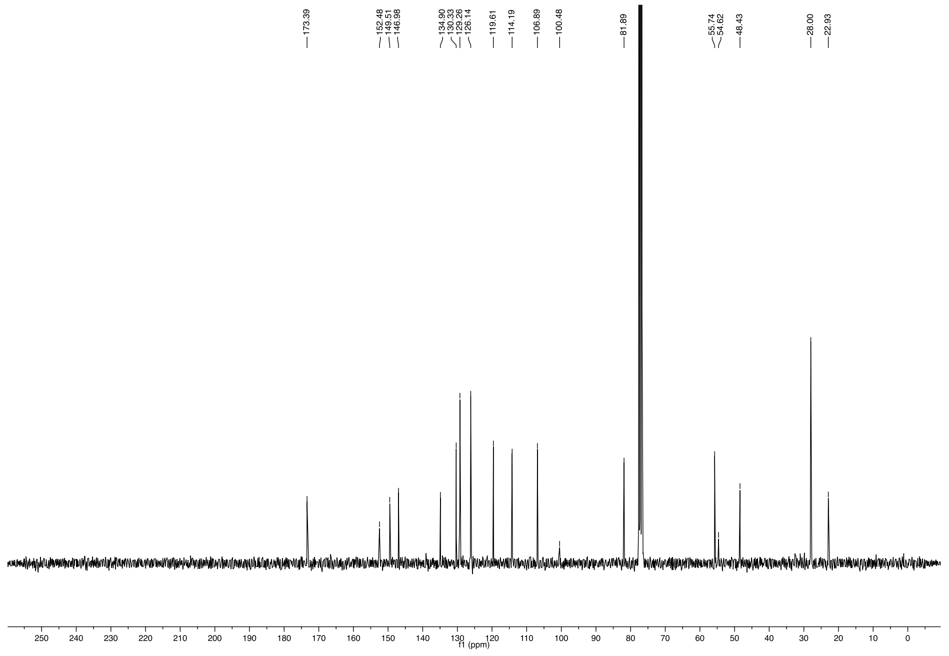

^{13}C-NMR

HPLC Chromatograms:

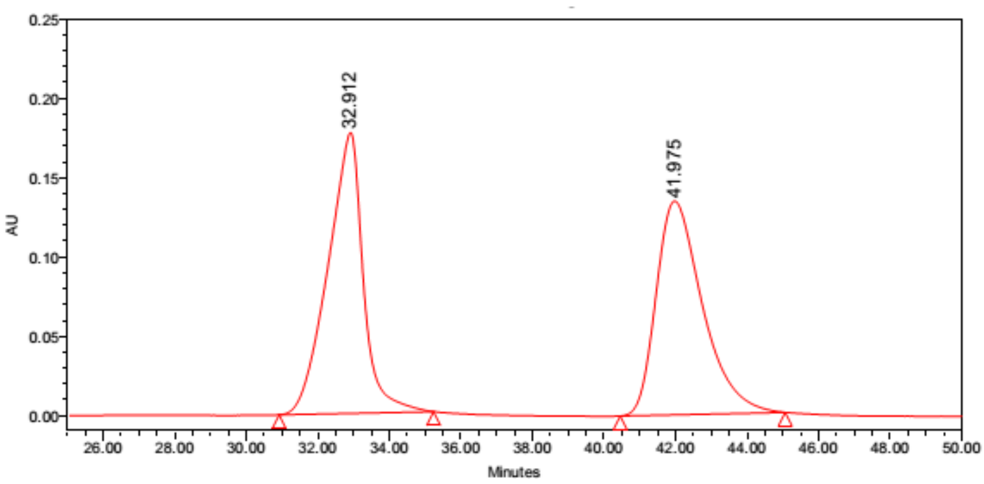

	RT	Area	% Area	Height
1	32.912	12130272	50.02	177036
2	41.975	12119762	49.98	134713

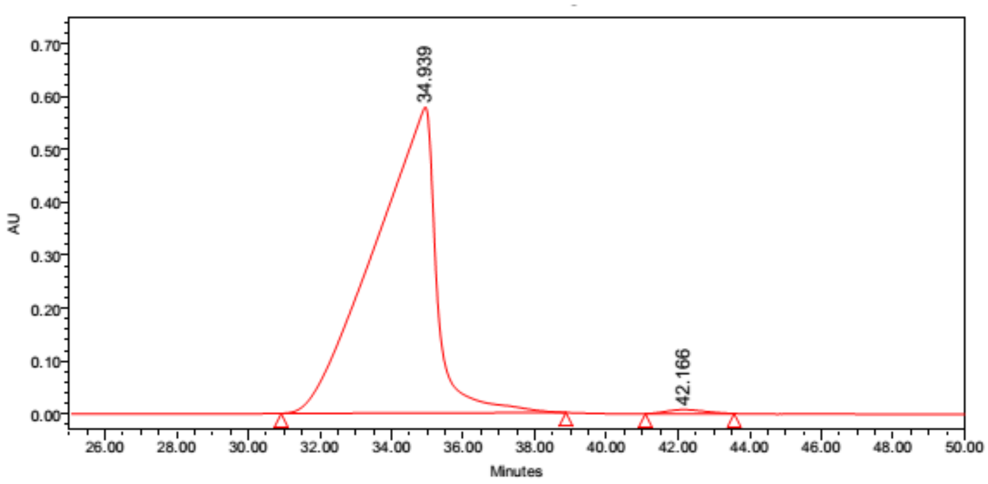

	RT	Area	% Area	Height
1	34.939	69944738	99.23	577401
2	42.166	543866	0.77	7421

***tert*-Butyl (3*S*,3a*R*,6a*R*)-3-{[3,5-bis(trifluoromethyl)phenyl]amino}-6a-(4-chlorophenyl)-2-oxo hexahydro-6*H*-furo[2,3-*b*]pyrrole-6-carboxylate [(+)-4b]**

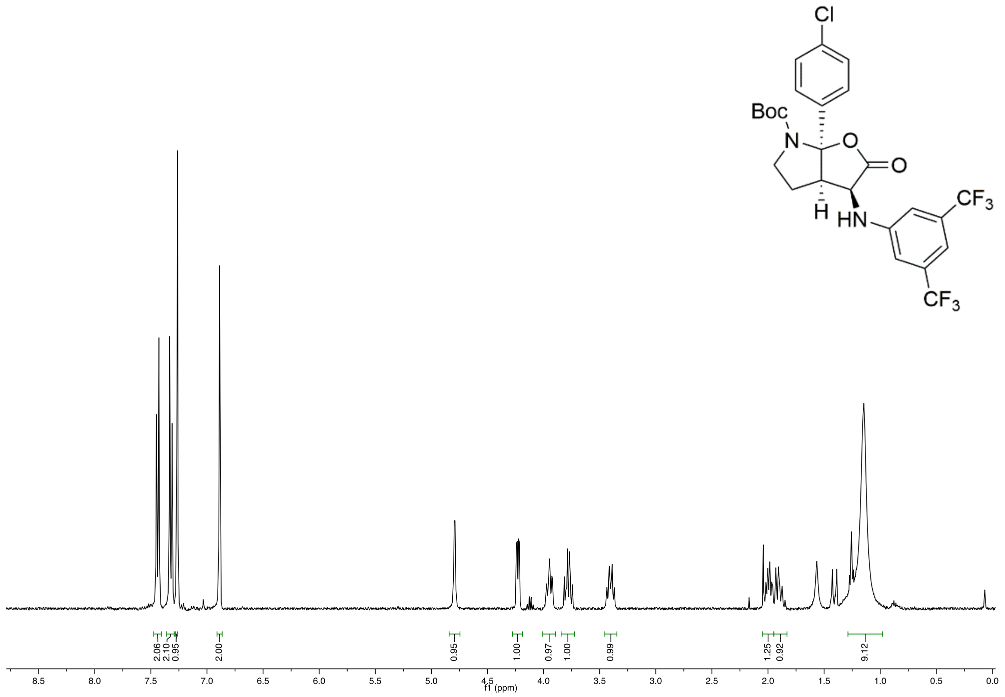

^{1}H-NMR

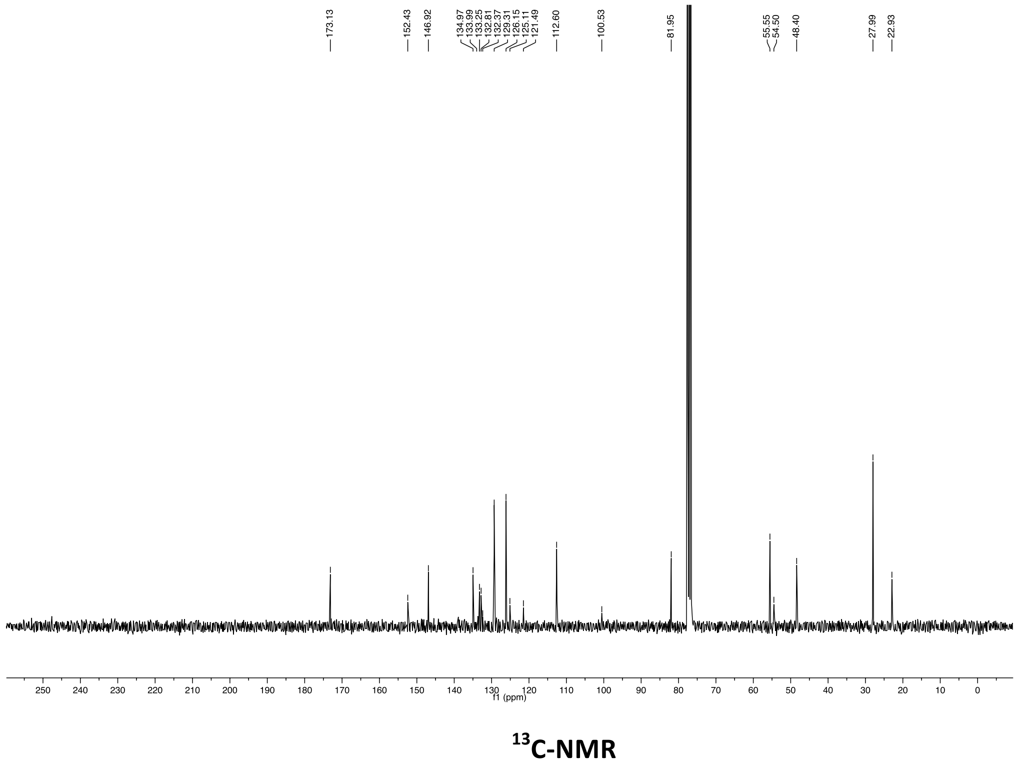

^{13}C-NMR

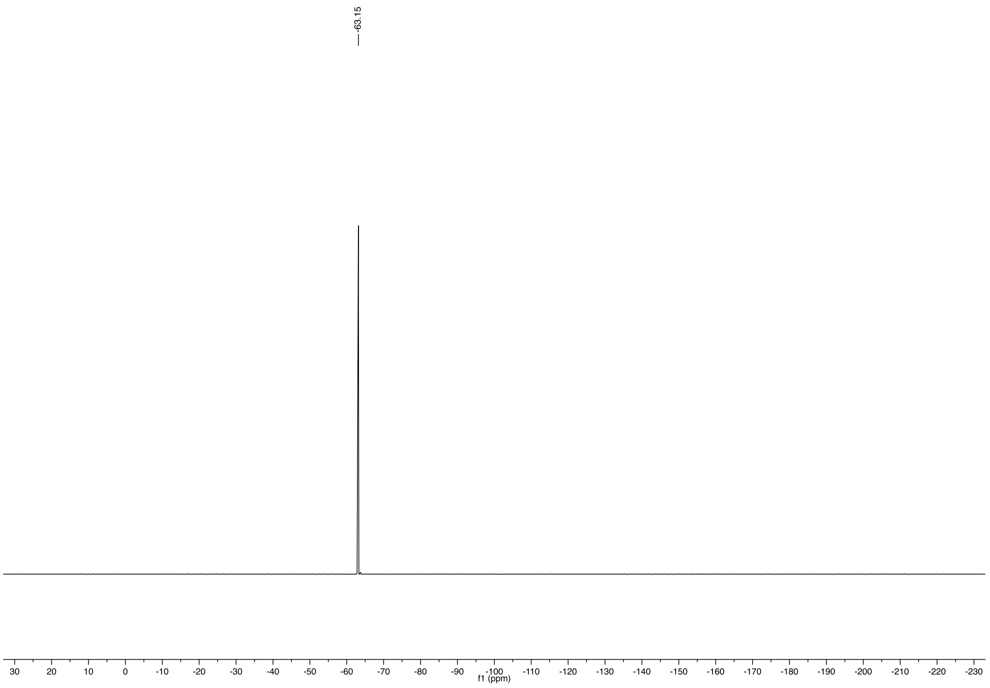
-63.15
30 20 10 0 -10 -20 -30 -40 -50 -60 -70 -80 -90 -100 -110 -120 -130 -140 -150 -160 -170 -180 -190 -200 -210 -220 -230
f1 (ppm)

^{19}F-NMR

HPLC Chromatograms:

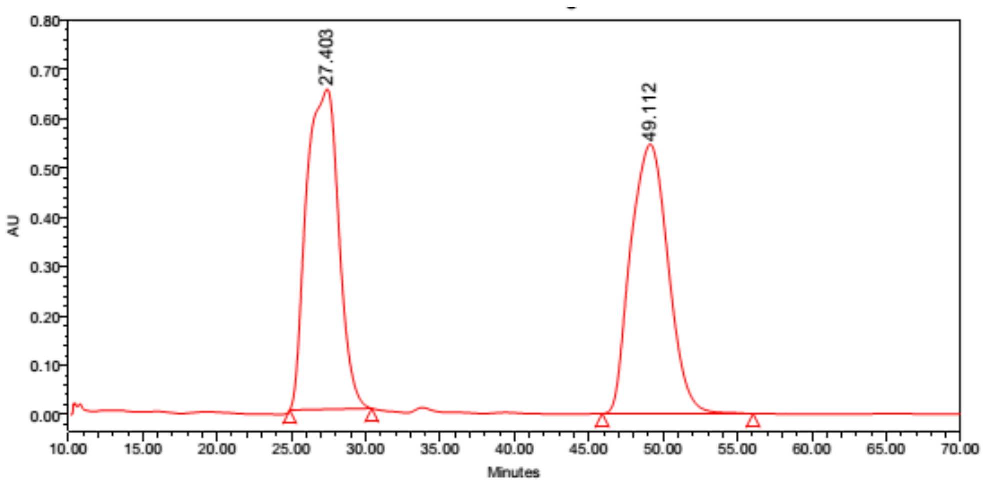

	RT	Area	% Area	Height
1	27.403	97898961	50.52	649527
2	49.112	95866746	49.48	546298

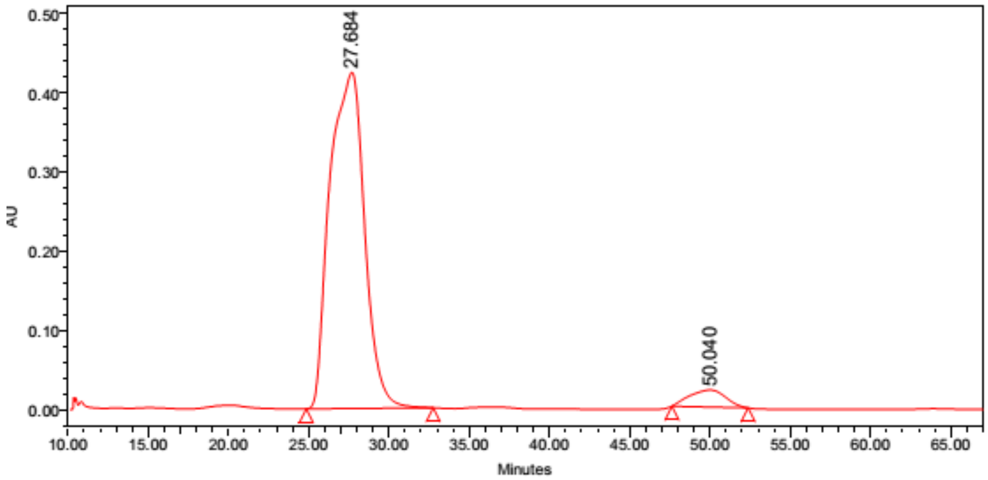

	RT	Area	% Area	Height
1	27.684	63644087	94.99	424196
2	50.040	3355935	5.01	21602

tert-Butyl (3_S_,3a_R_,6a_R_)-6a-(4-chlorophenyl)-3-[(2-fluoro-5-nitrophenyl)amino]-2-oxohexahydro-6_H_-furo[2,3-_b_]pyrrole-6-carboxylate [(+)-4c]

* Signals marked with an asterisk correspond to _tert_-butyl [4-(4-chlorophenyl)-4-oxobutyl]carbamate. This compound could not be separated from the final product.

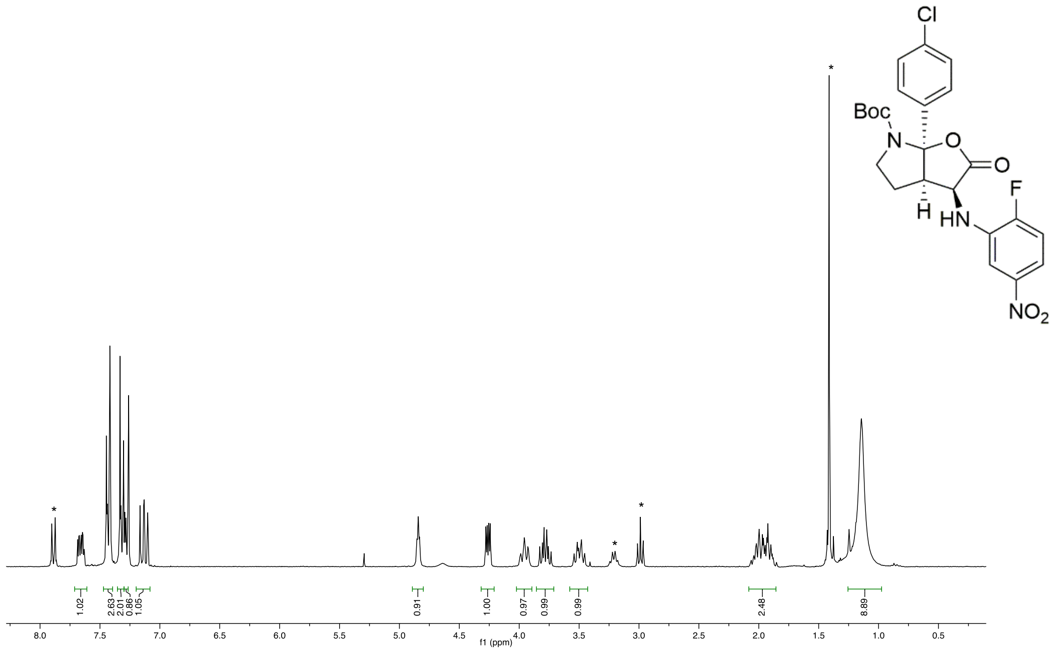

^{1}H-NMR

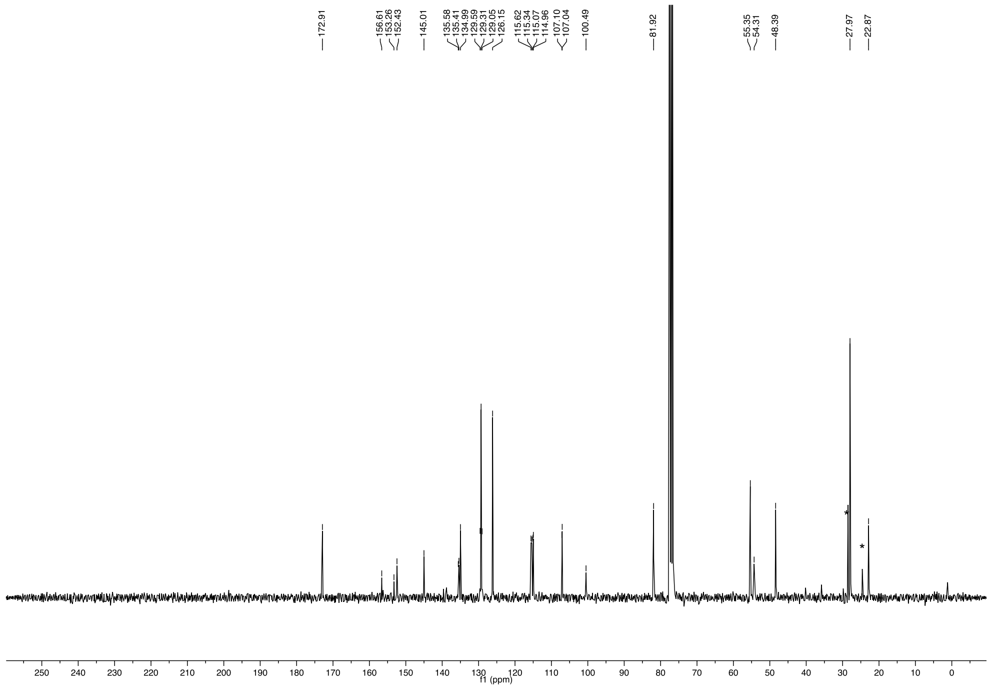

^{13}C-NMR

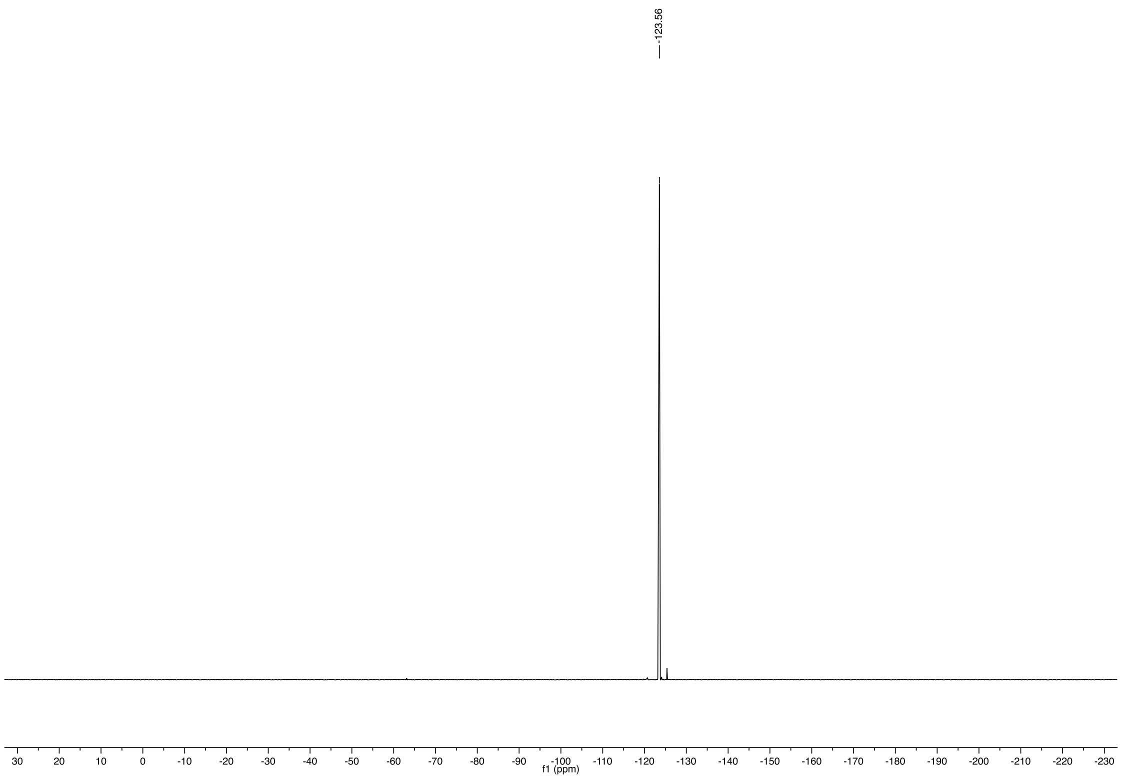

-123.56
30
20
10
0
-10
-20
-30
-40
-50
-60
-70
-80
-90
-100
-110
-120
-130
-140
-150
-160
-170
-180
-190
-200
-210
-220
-230
f1 (ppm)

^{19}F-NMR

HPLC Chromatograms:

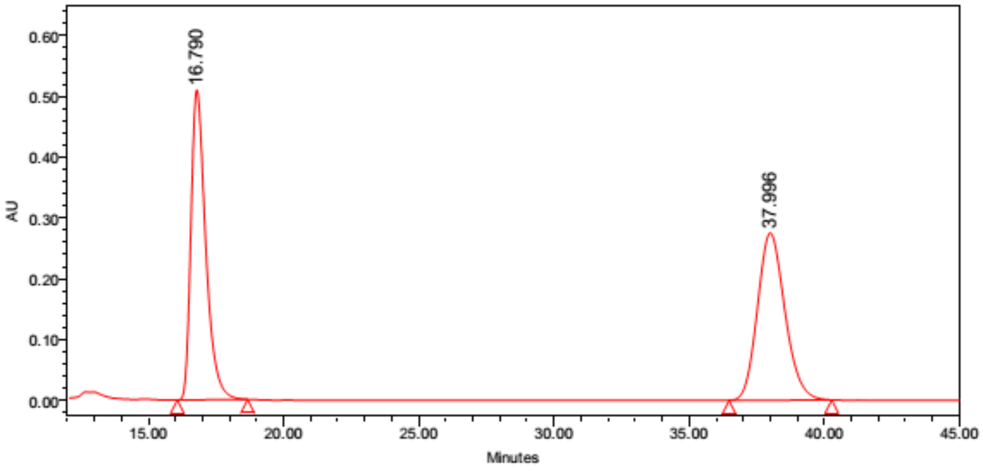

	RT	Area	% Area	Height
1	16.790	19323975	49.86	510153
2	37.996	19430101	50.14	275073

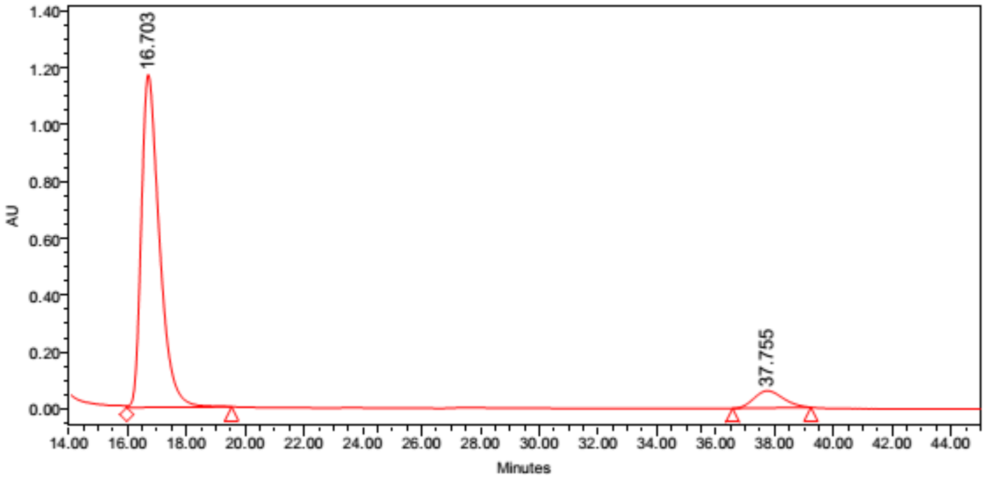

	RT	Area	% Area	Height
1	16.703	49889192	92.24	1171491
2	37.755	4198688	7.76	60530

***tert*-Butyl (3*S*,3a*R*,6a*R*)-3-[(3-nitrophenyl)amino]-2-oxo-6a-phenylhexahydro-6*H*-furo[2,3-*b*] pyrrole-6-carboxylate [(+)-4d]**

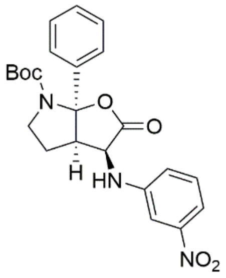

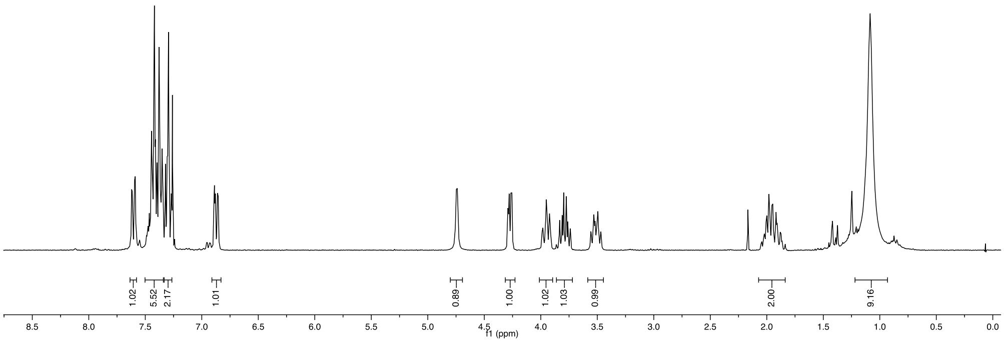

^{1}H-NMR

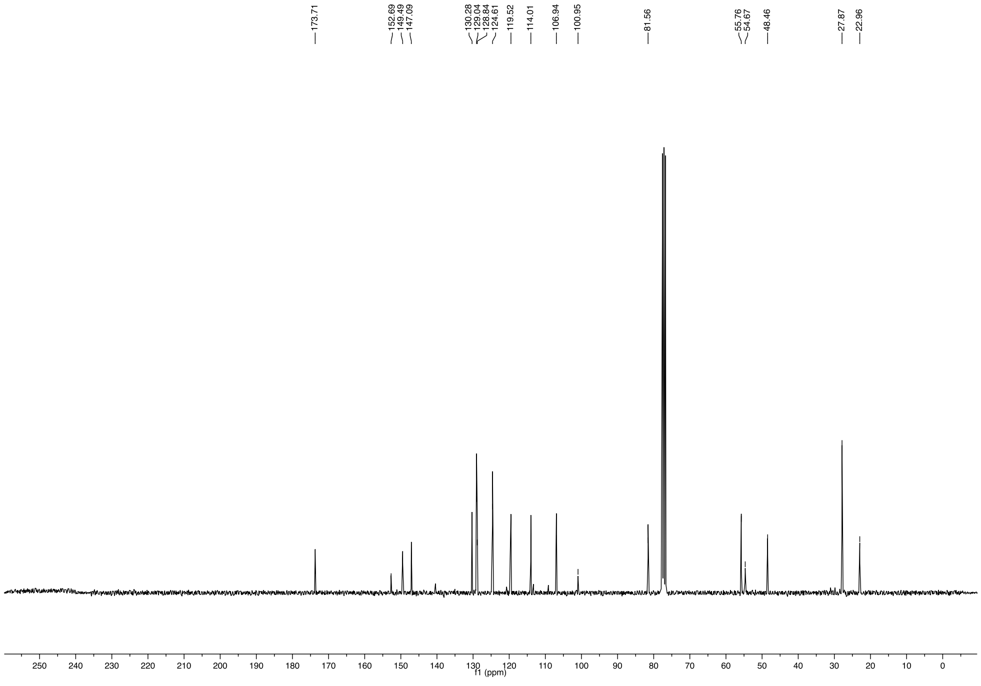

^{13}C-NMR

HPLC Chromatograms:

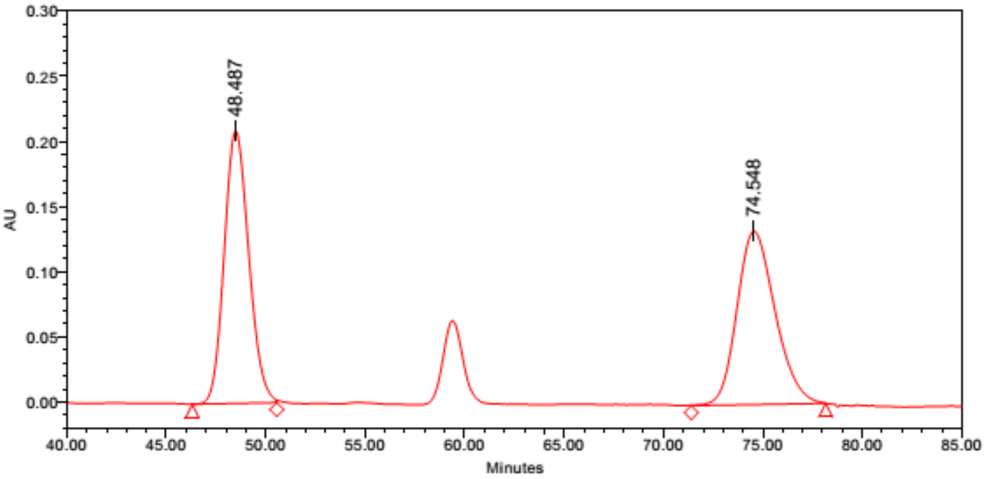

	RT	Area	% Area	Height
1	48.487	17935698	50.27	208909
2	74.548	17743000	49.73	133157

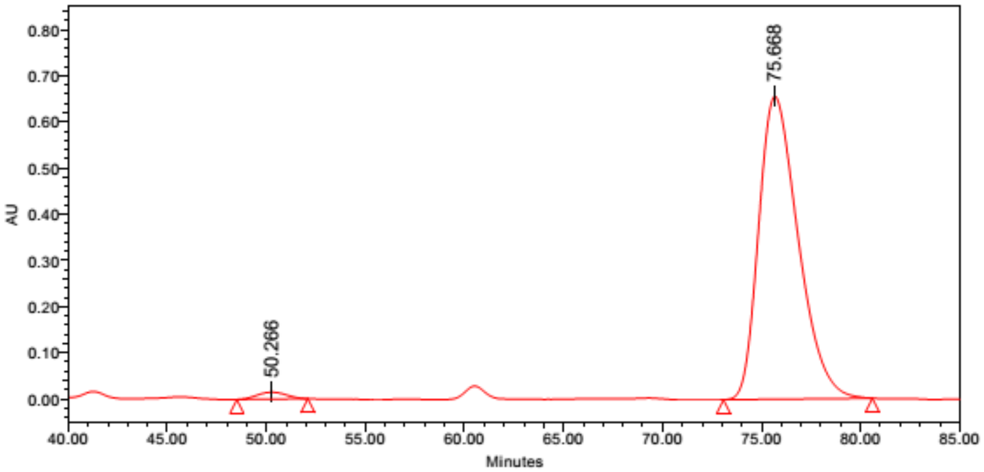

	RT	Area	% Area	Height
1	50.266	1534432	1.66	14821
2	75.668	90938758	98.34	655920

***tert*-Butyl (3*S*,3a*R*,6a*R*)-3-{[3,5-bis(trifluoromethyl)phenyl]amino}-2-oxo-6a-phenylhexahydro -6*H*-furo[2,3-*b*]pyrrole-6-carboxylate [(+)-4e]**

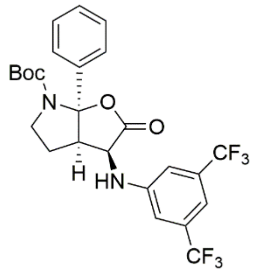

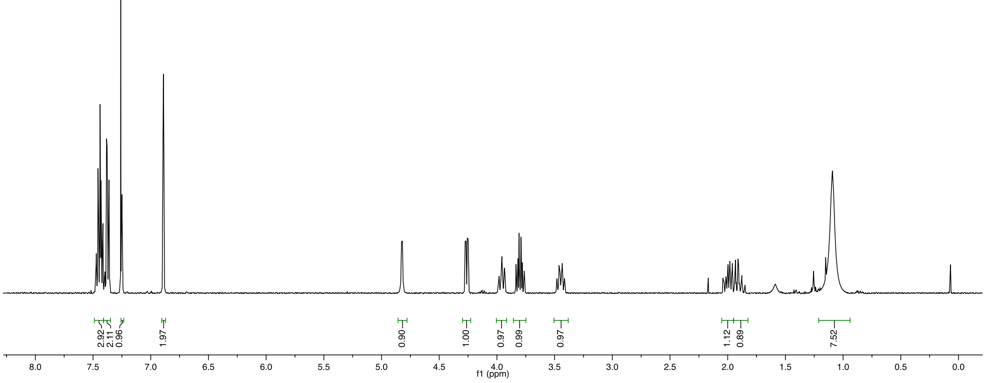

^{1}H-NMR

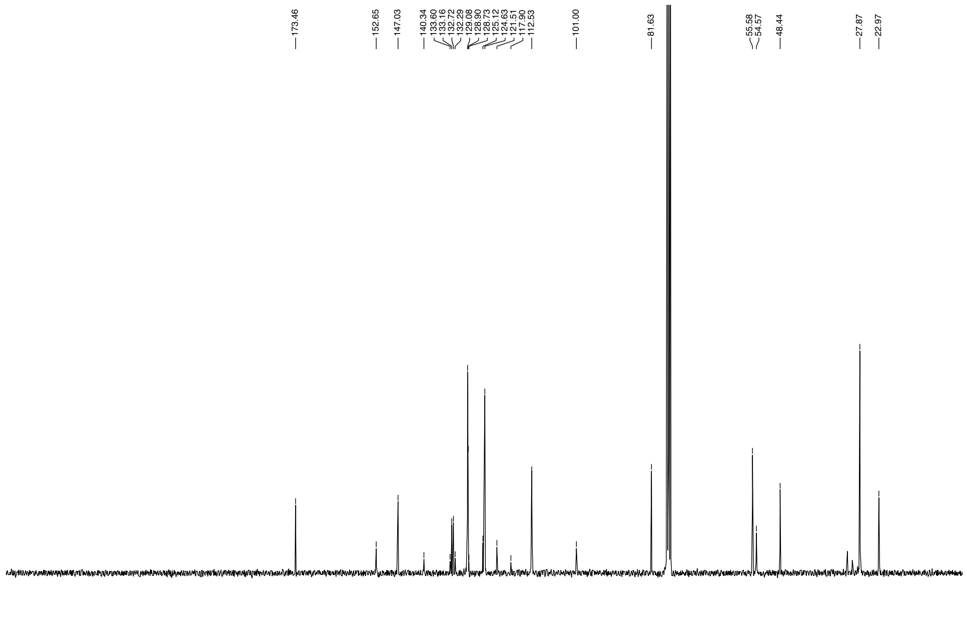

^{13}C-NMR

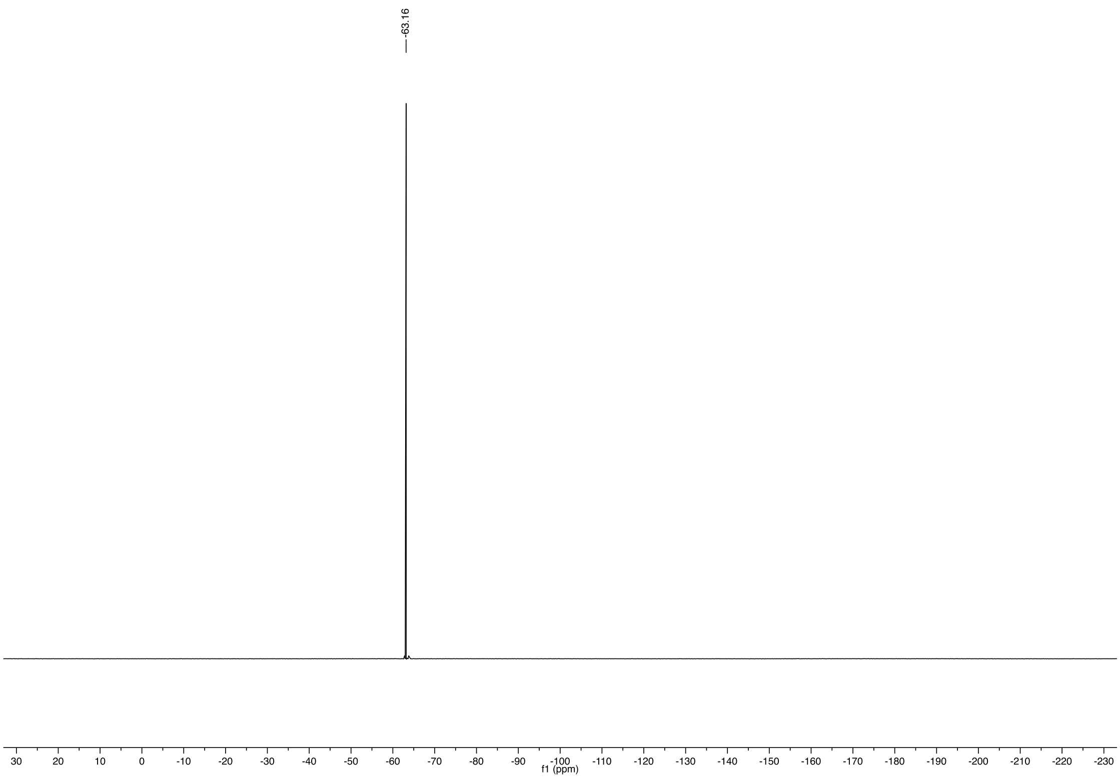

-63.16
30
20
10
0
-10
-20
-30
-40
-50
-60
-70
-80
-90
-100
-110
-120
-130
-140
-150
-160
-170
-180
-190
-200
-210
-220
-230
f1 (ppm)

^{19}F-NMR

HPLC Chromatograms:

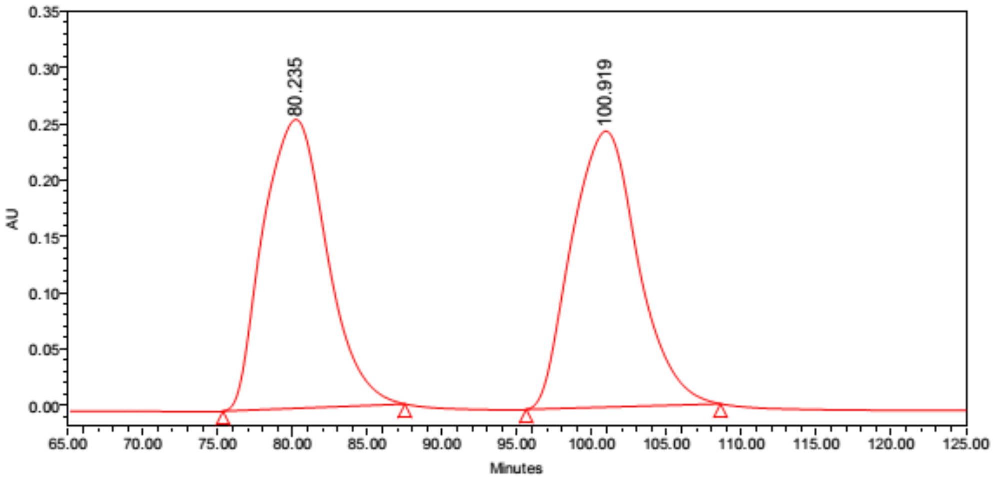

	RT	Area	% Area	Height
1	80.235	75933885	50.07	256329
2	100.919	75711923	49.93	244899

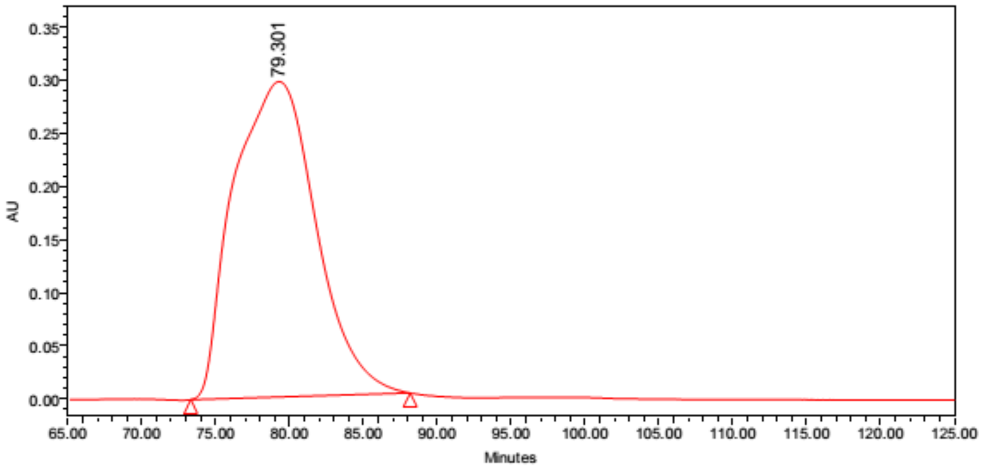

	RT	Area	% Area	Height
1	79.301	115148733	100.00	296948

***tert*-Butyl (3*S*,3a*R*,6a*R*)-6a-(4-methoxyphenyl)-3-[(3-nitrophenyl)amino]-2-oxo hexahydro-6*H*-furo[2,3-*b*]pyrrole-6-carboxylate [(+)-4f]**

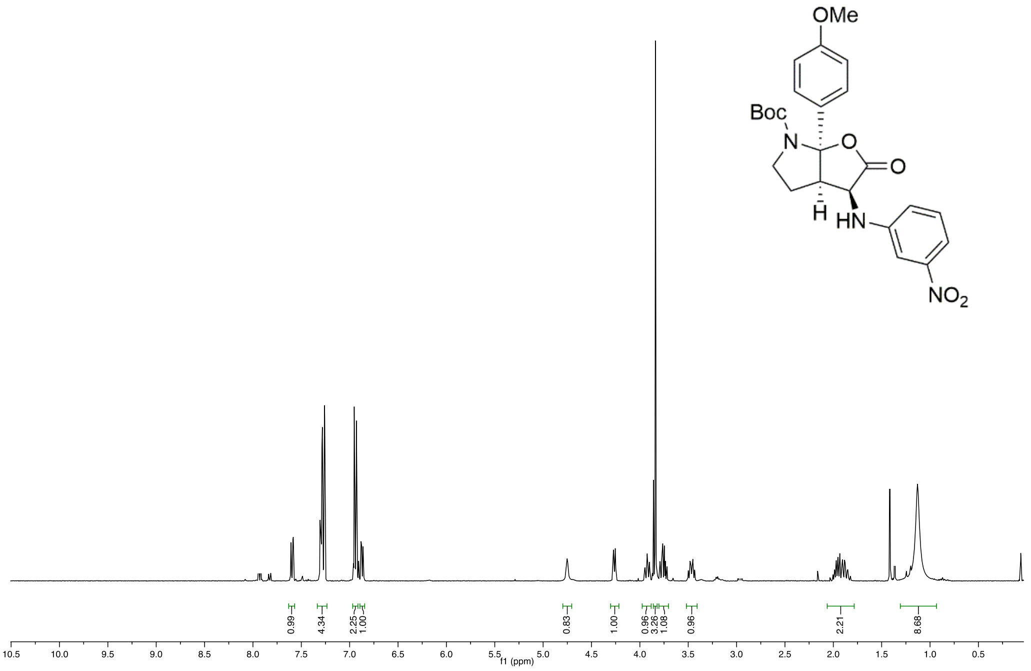

^{1}H-NMR

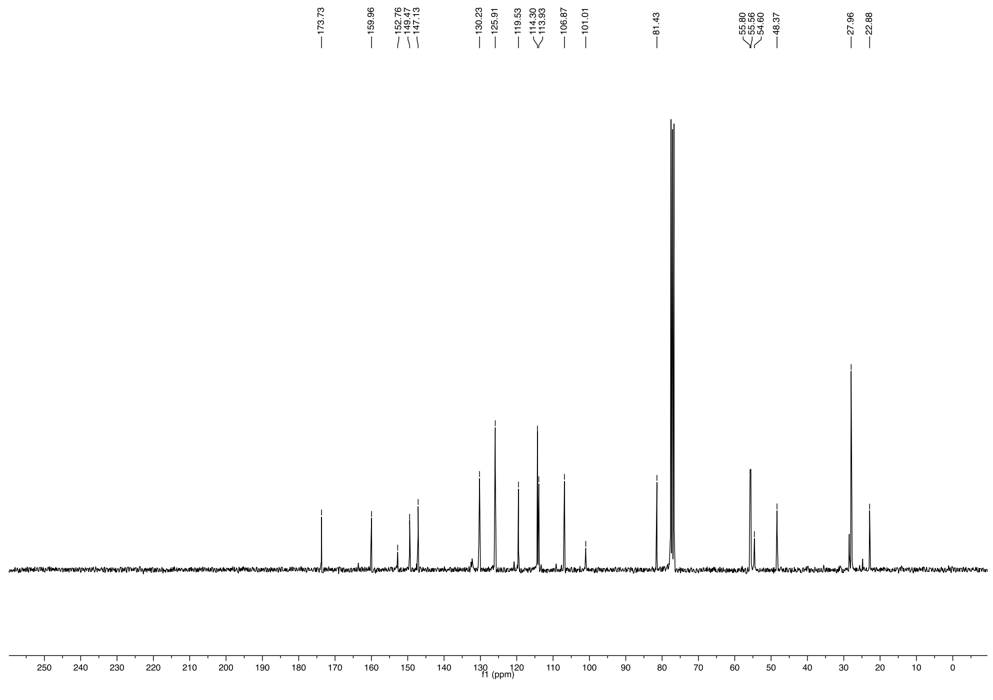

^{13}C-NMR

HPLC Chromatograms:

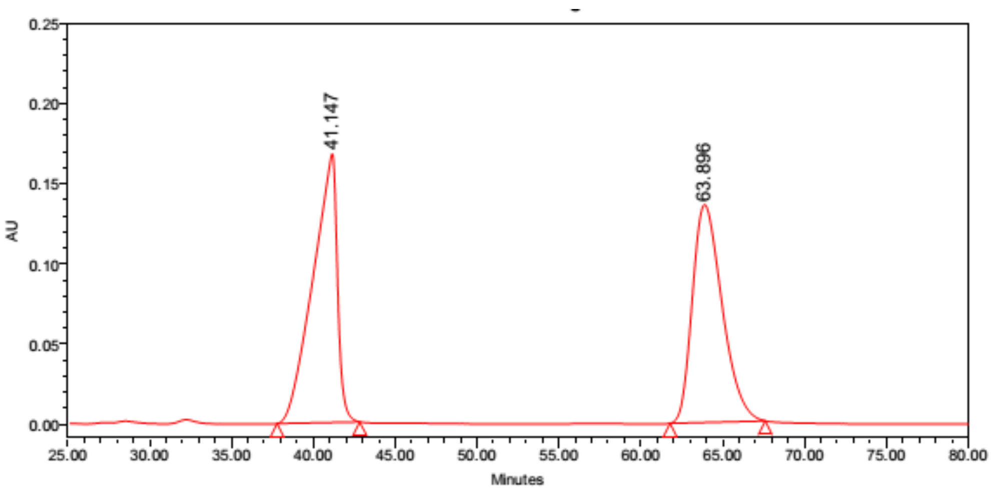

	RT	Area	% Area	Height
1	41.147	17340620	50.24	167462
2	63.896	17175273	49.76	135675

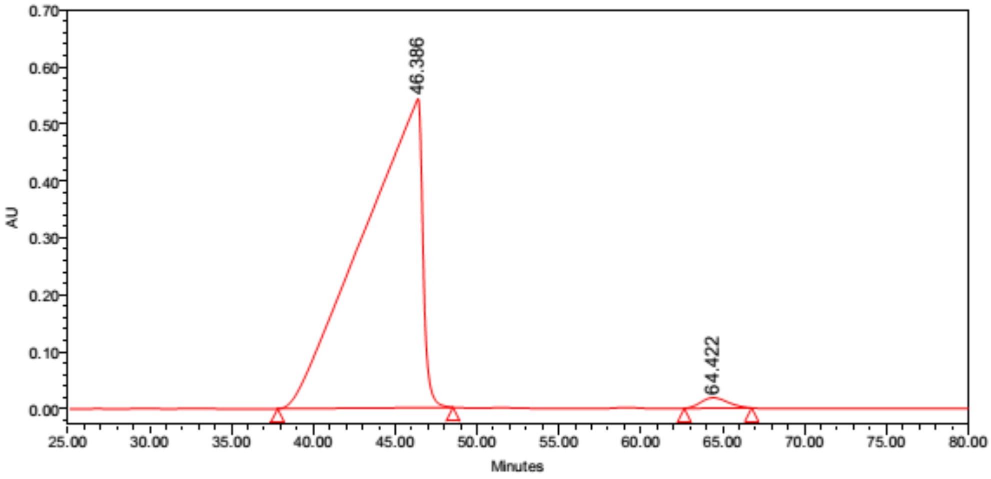

	RT	Area	% Area	Height
1	46.386	137261544	98.51	541982
2	64.422	2079204	1.49	18178

***tert*-Butyl (3*S*,3a*R*,6a*R*)-3-{[3,5-bis(trifluoromethyl)phenyl]amino}-6a-(4-methoxy phenyl)-2-oxohexahydro-6*H*-furo[2,3-*b*]pyrrole-6-carboxylate [(+)-4g]**

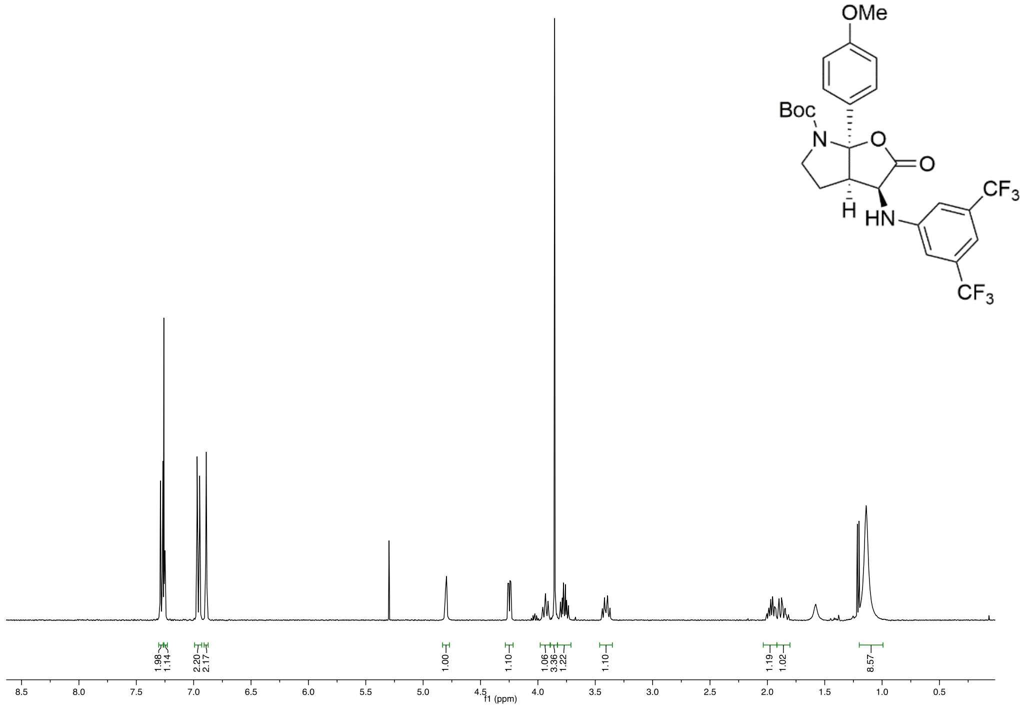

^{1}H-NMR

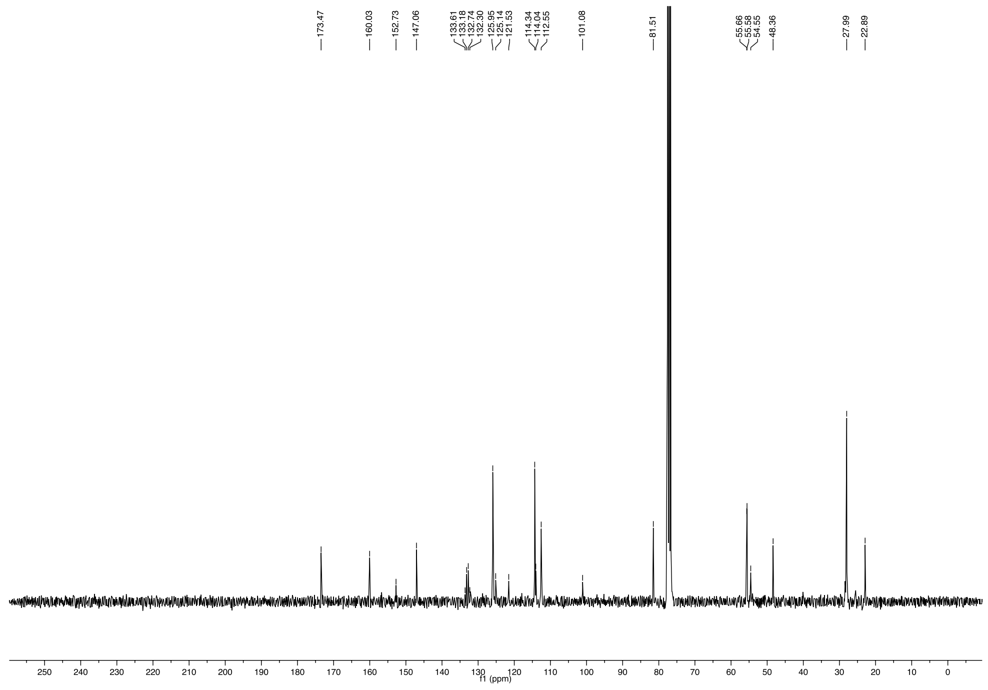

^{13}C-NMR

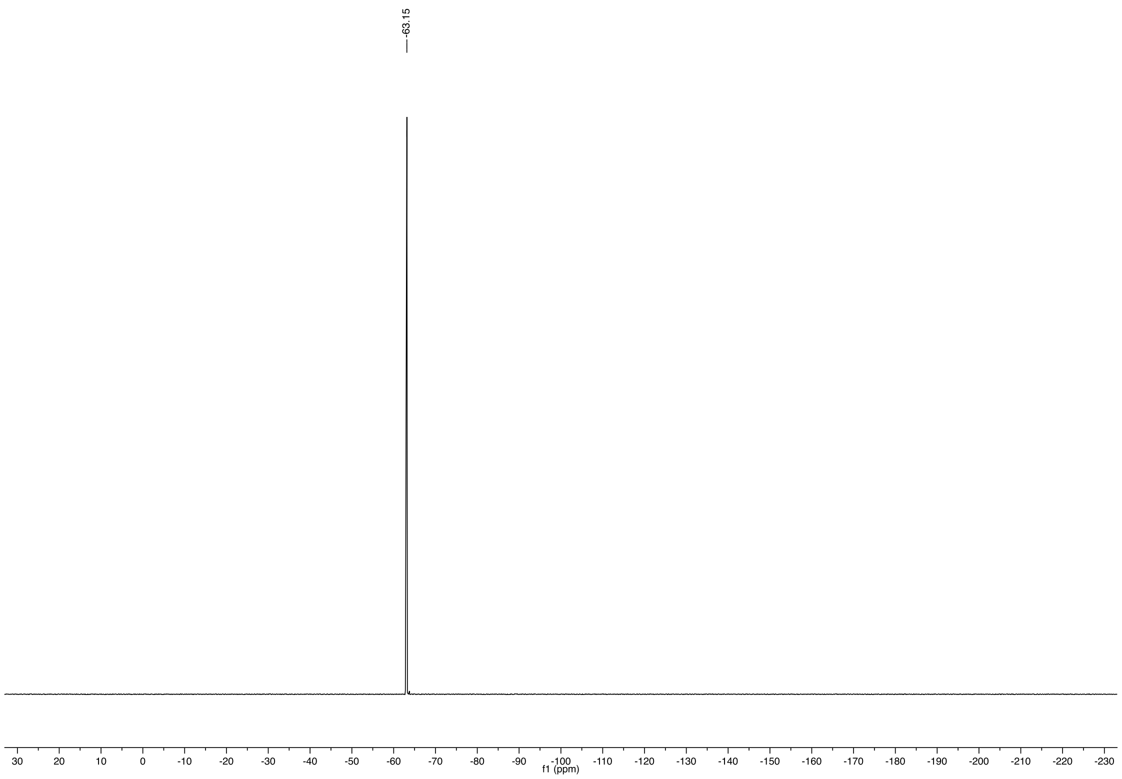
-63.15
30
20
10
0
-10
-20
-30
-40
-50
-60
-70
-80
-90
-100
-110
-120
-130
-140
-150
-160
-170
-180
-190
-200
-210
-220
-230
f1 (ppm)

^{19}F-NMR

HPLC Chromatograms:

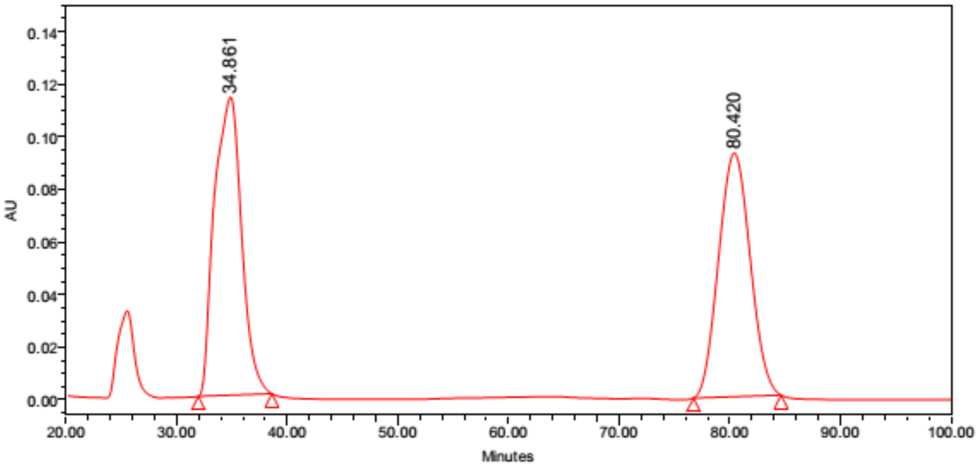

	RT	Area	% Area	Height
1	34.861	19300484	51.22	113291
2	80.420	18380905	48.78	92634

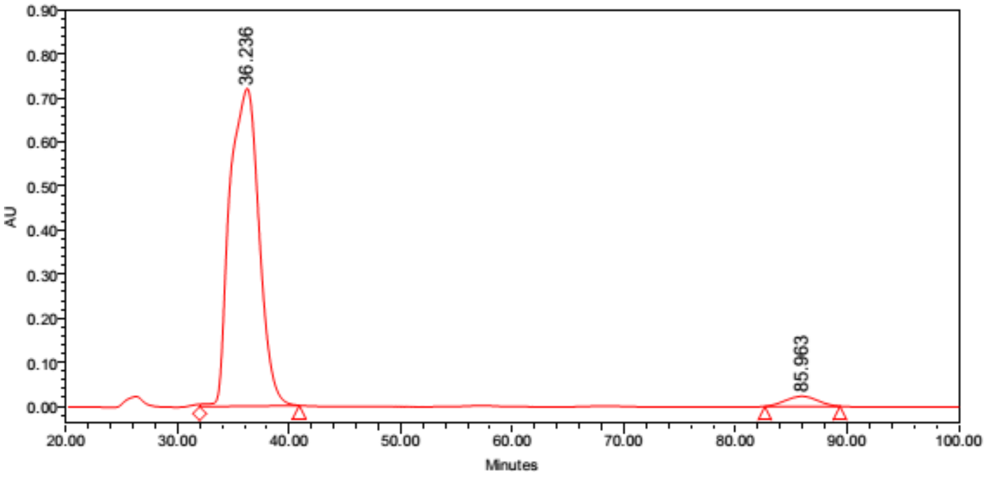

	RT	Area	% Area	Height
1	36.236	131053314	96.65	720832
2	85.963	4547454	3.35	22753

***tert*-Butyl (3*S*,3a*R*,6a*R*)-3-[(3-nitrophenyl)amino]-2-oxo-6a-(*p*-tolyl)hexahydro-6*H*-furo[2,3-*b*] pyrrole-6-carboxylate [(+)-4h]**

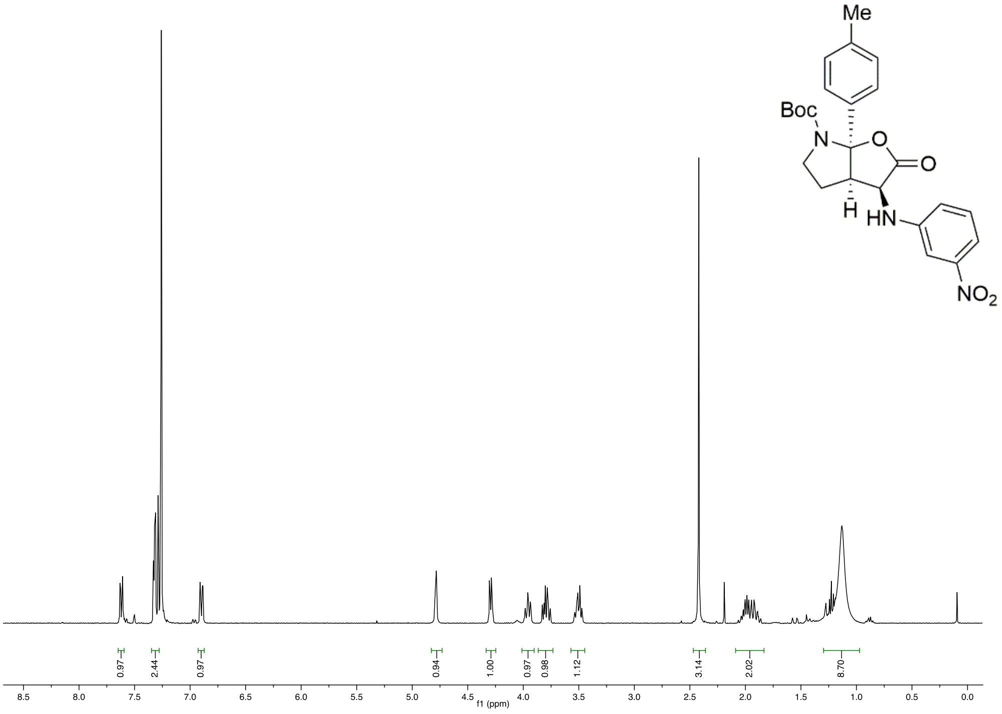

^{1}H-NMR

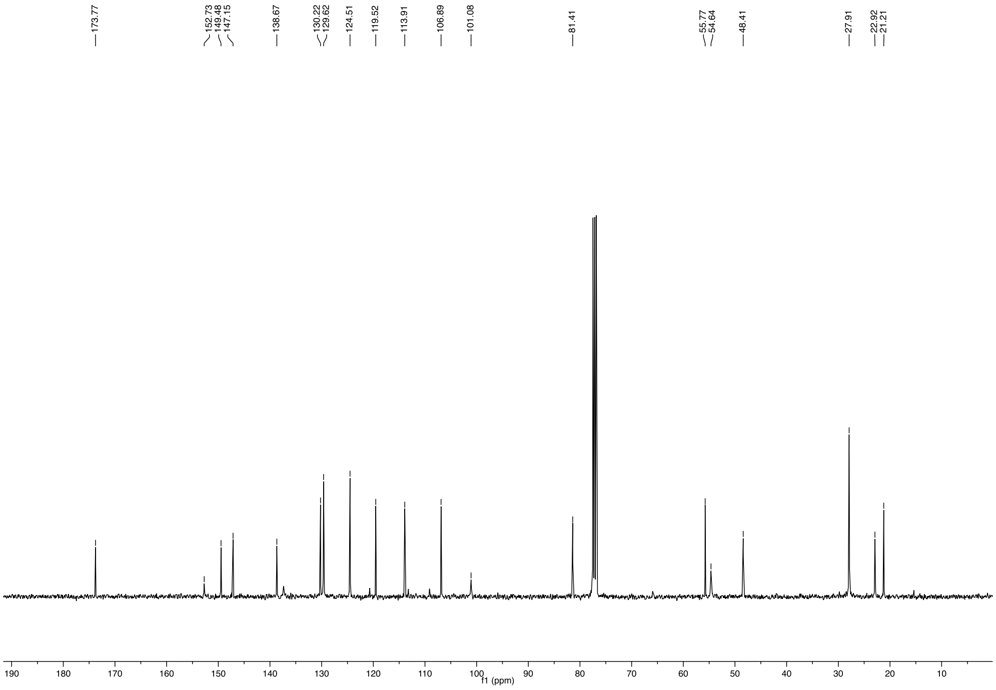

^{13}C-NMR

HPLC Chromatograms:

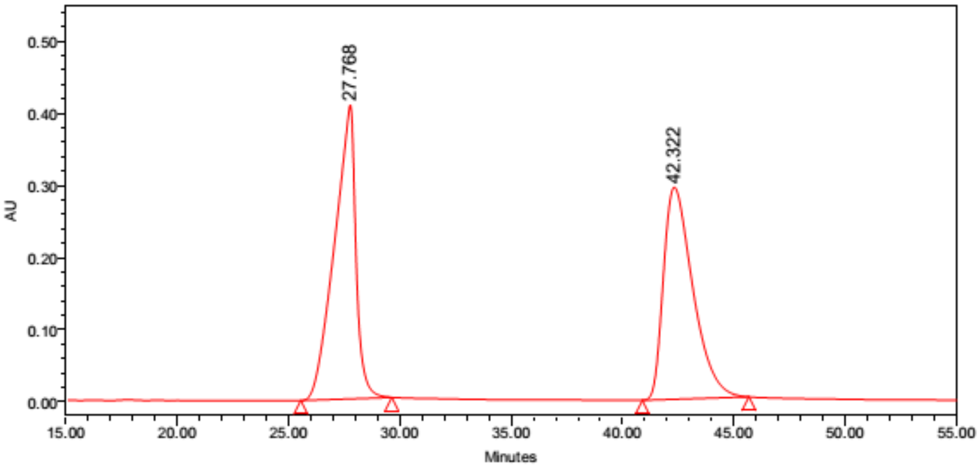

	RT	Area	% Area	Height
1	27.768	27259089	50.21	407274
2	42.322	27036070	49.79	294005

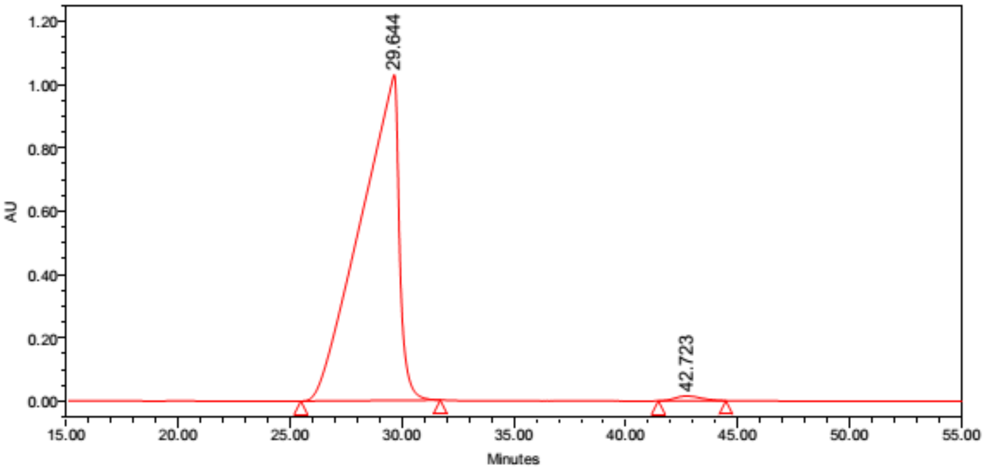

	RT	Area	% Area	Height
1	29.644	123427528	99.01	1028468
2	42.723	1239676	0.99	15247

Methyl (3*S*,3a*R*,6a*R*)-3-[(3-nitrophenyl)amino]-2-oxo-6a-phenylhexahydro-6*H*-furo[2,3-*b*] pyrrole-6-carboxylate [(+)-4i]

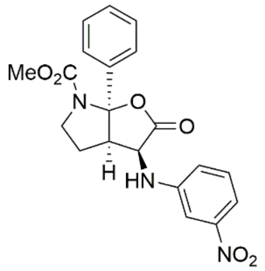

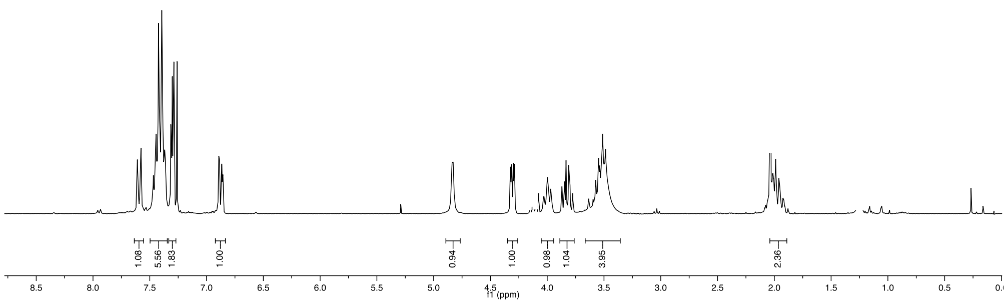

^{1}H-NMR

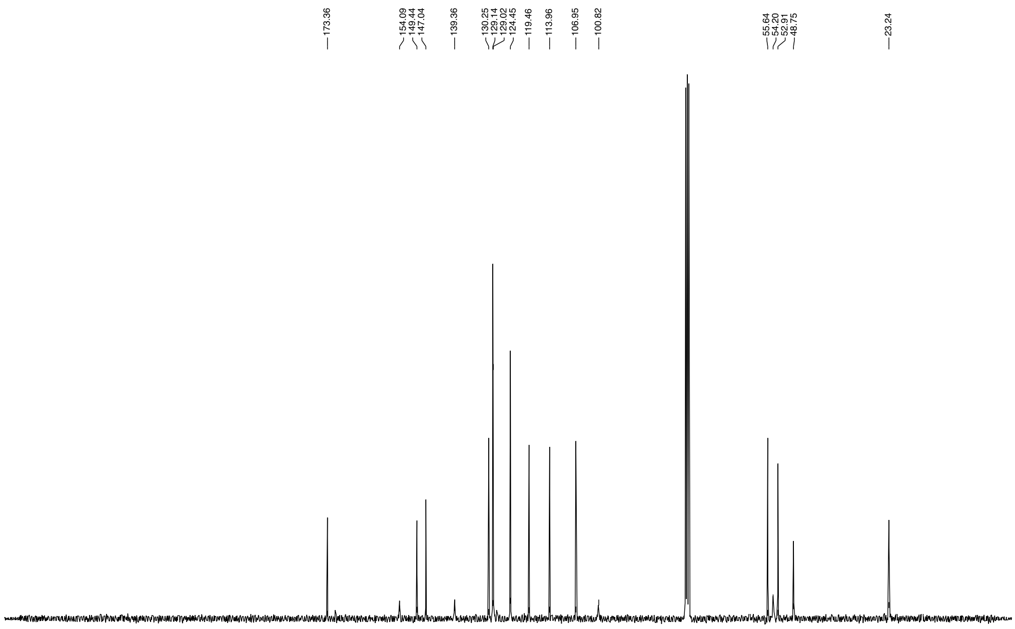

^{13}C-NMR

HPLC Chromatograms:

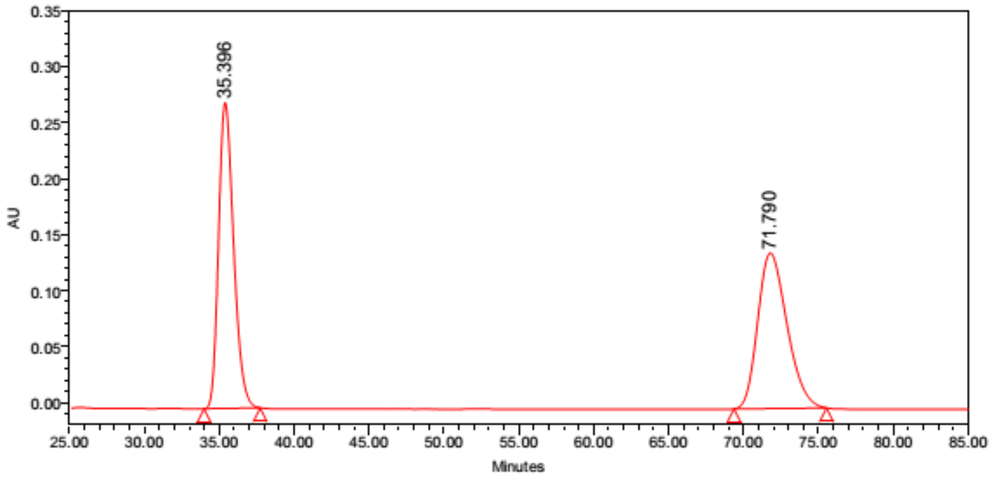

	RT	Area	% Area	Height
1	35.396	19057294	50.27	273107
2	71.790	18849374	49.73	138924

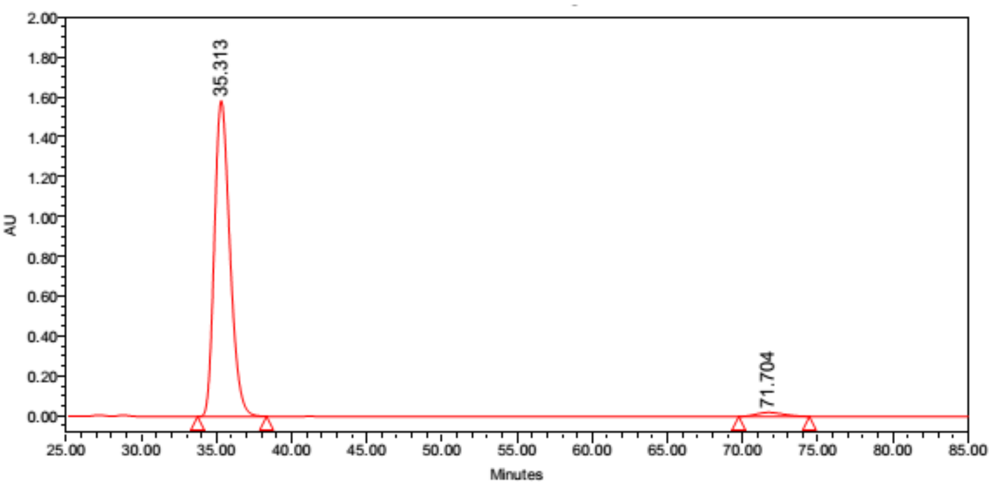

	RT	Area	% Area	Height
1	35.313	113559795	97.76	1584947
2	71.704	2603696	2.24	20410

Methyl (3*S*,3a*R*,6a*R*)-3-{[2-fluoro-3-(trifluoromethyl)phenyl]amino}-2-oxo-6a-phenylhexahydro-6*H*-furo[2,3-*b*]pyrrole-6-carboxylate [(+)-4j]

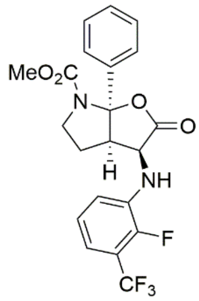

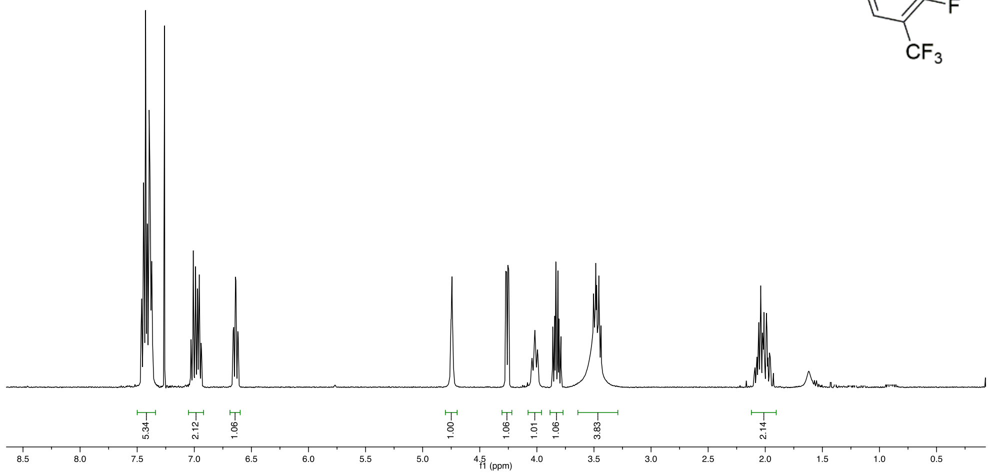

^{1}H-NMR

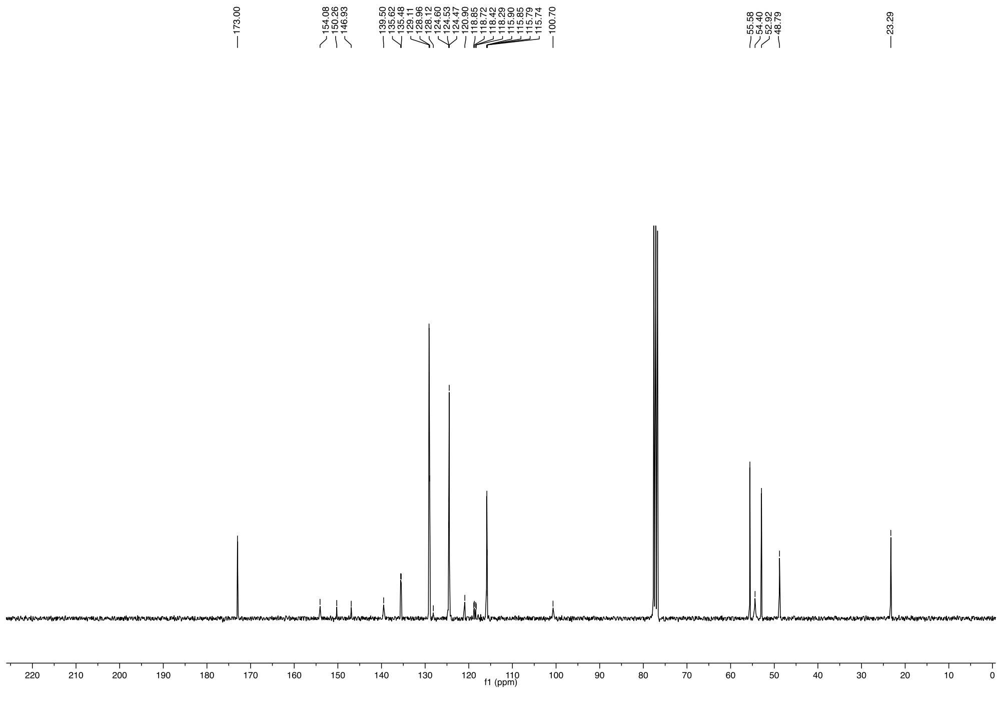

^{13}C-NMR

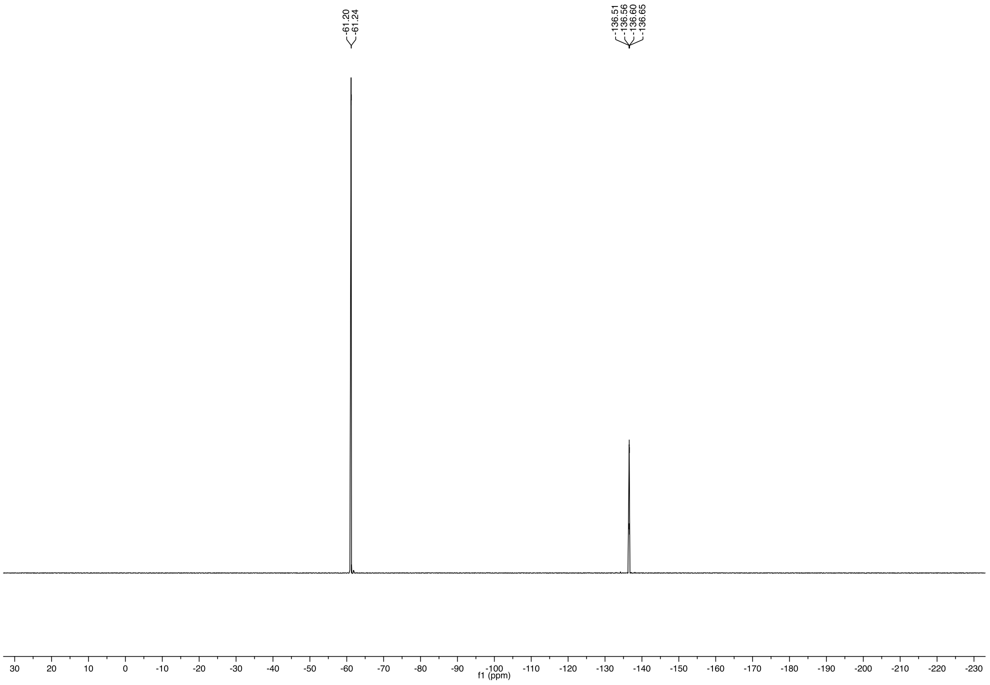
-61.20
-61.24
-136.51
-136.56
-136.60
-136.65
30 20 10 0 -10 -20 -30 -40 -50 -60 -70 -80 -90 -100 -110 -120 -130 -140 -150 -160 -170 -180 -190 -200 -210 -220 -230
f1 (ppm)

^{19}F-NMR

HPLC Chromatograms:

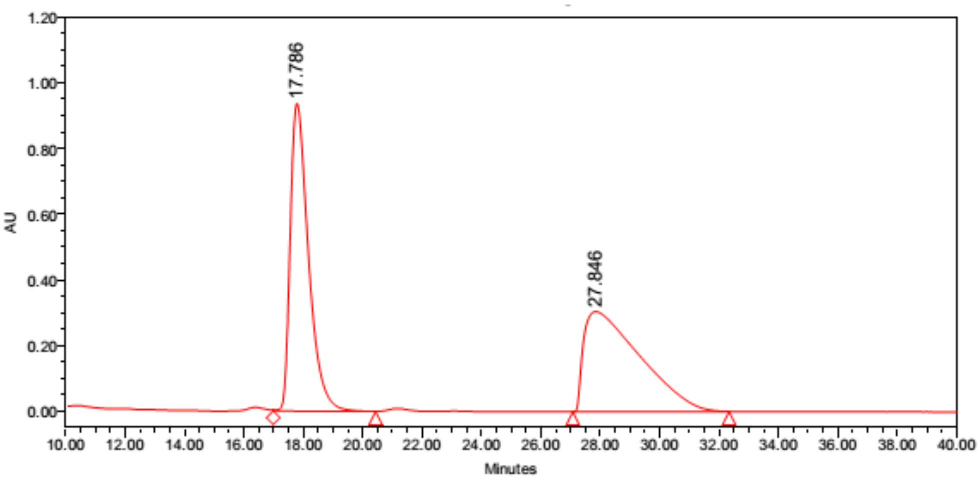

	RT	Area	% Area	Height
1	17.786	39913404	49.81	936719
2	27.846	40214652	50.19	304790

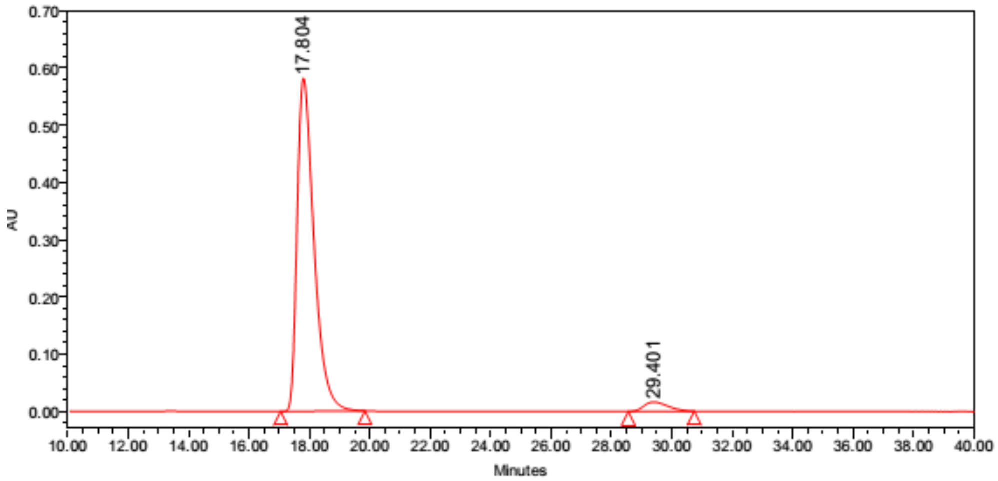

	RT	Area	% Area	Height
1	17.804	22537760	96.13	582169
2	29.401	907038	3.87	16382

Methyl (3*S*,3a*R*,6a*R*)-3-[(2-fluoro-5-nitrophenyl)amino]-2-oxo-6a-phenylhexahydro-6*H*-furo [2,3-*b*]pyrrole-6-carboxylate [(+)-4k]

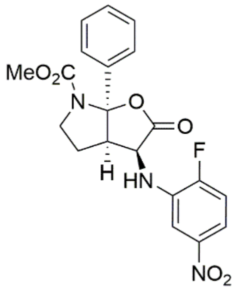

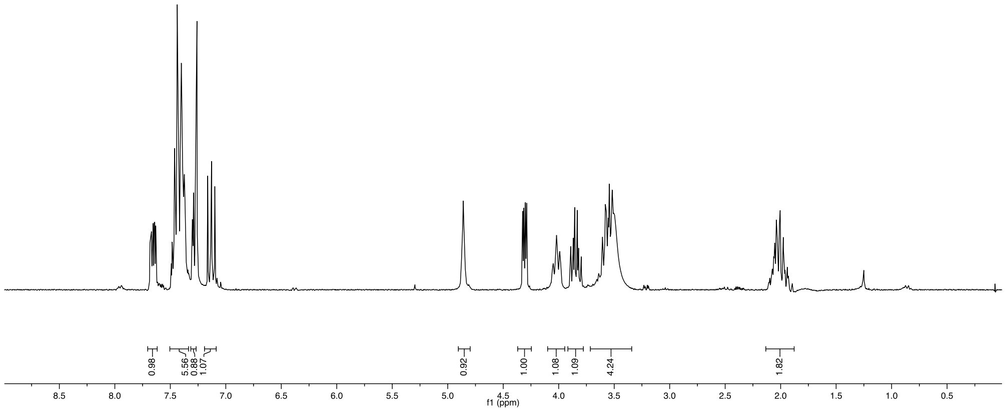

^{1}H-NMR

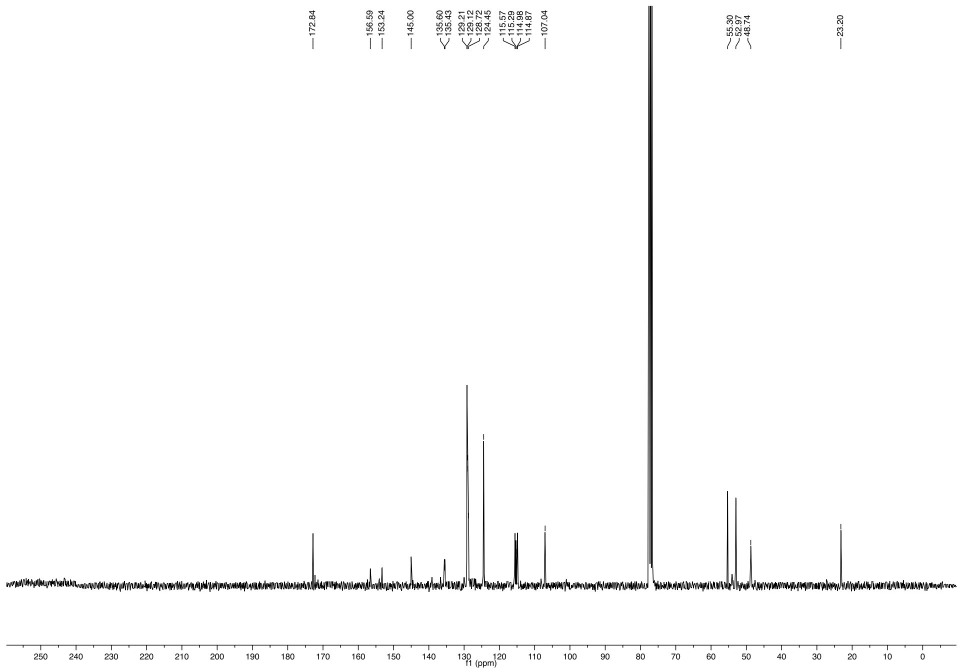

^{13}C-NMR

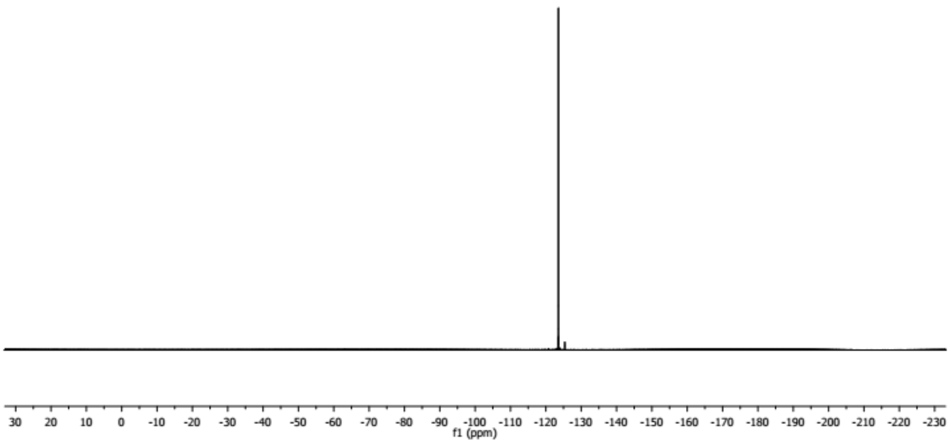

30 20 10 0 -10 -20 -30 -40 -50 -60 -70 -80 -90 -100 -110 -120 -130 -140 -150 -160 -170 -180 -190 -200 -210 -220 -230
f1 (ppm)

^{19}F-NMR

HPLC Chromatograms:

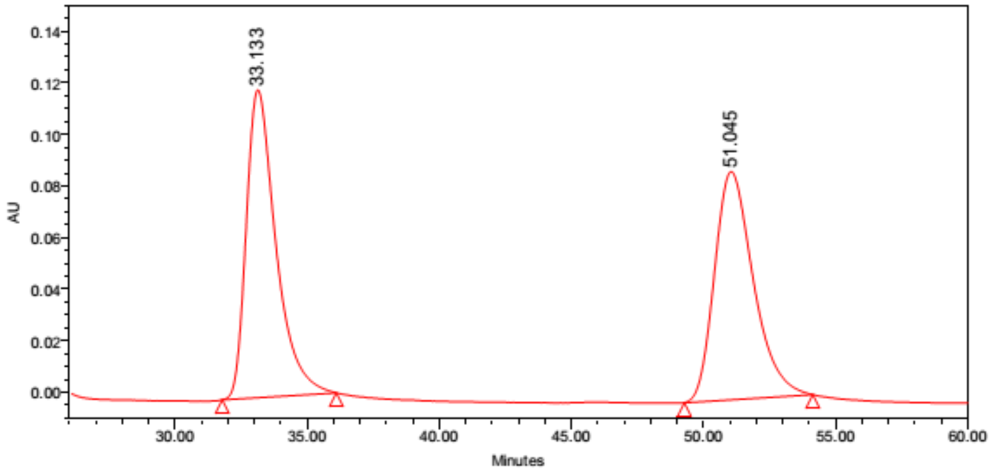

	RT	Area	% Area	Height
1	33.133	9300738	50.72	119376
2	51.045	9034980	49.28	88493

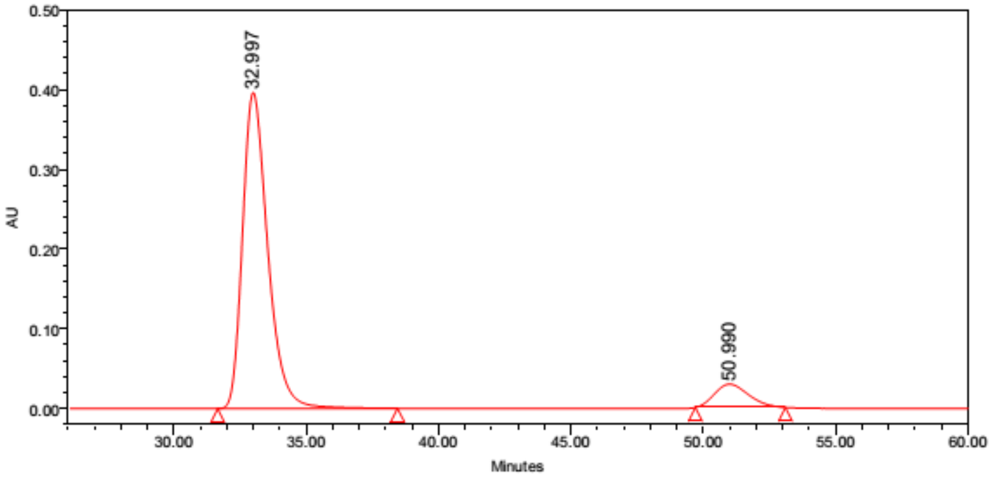

	RT	Area	% Area	Height
1	32.997	26198169	90.94	396413
2	50.990	2609945	9.06	29199

4. X-Ray

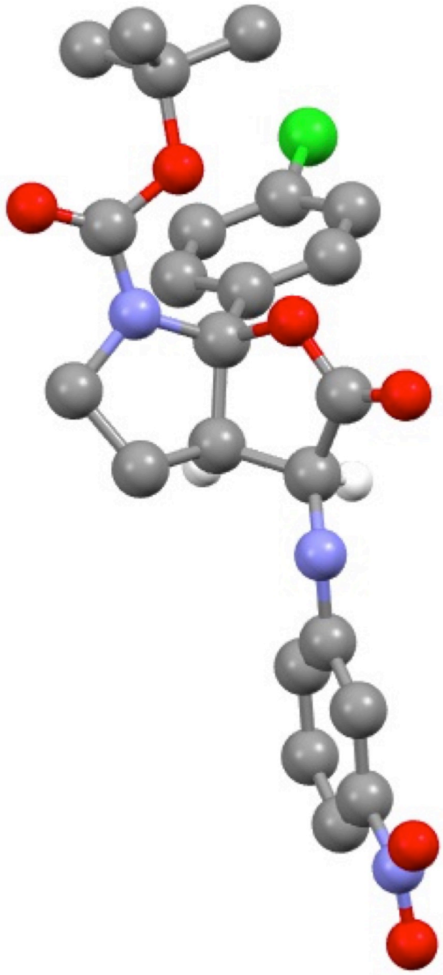

Figure. Ortep representation of *tert*-butyl (3*S*,3a*R*,6a*R*)-6a-(4-chlorophenyl)-3-[(3-nitrophenyl)amino]-2-oxohexahydro-6*H*-furo[2,3-*b*]pyrrole-6-carboxylate [**(+)-4a**]

5. Computational Details

5.1. Computational Methods

All calculations were carried out using the Gaussian 09 program package[2] and UωB97XD functional developed by Chai and Head-Gordon.[3] The def2SVPP basis set developed by Ahlrichs and co-workers was used. Single point energy calculations were carried out with a triple ζ basis (def2TZVPP). The SMD model was used to include the solvent (toluene) in both, optimizations and single point calculations. The nature of the different saddle points was determined by the number of imaginary frequencies, and these structures were connected via IRC. All the energies showed in the manuscript are calculated at the sum of the high-basis set electronic energies plus the thermochemistry corrections at standard conditions (298.15K and 1 atm) adding 1.89 kcal/mol to account for standard concentration (1M). The free energy values were corrected employing the Quasi-Harmonic Approximation with the software GoodVibes developed by Paton and co-workers.[4] Specifically using the free-rotor approximation for vibrational frequencies as described by Grimme[5] with a cutoff of 100 cm^{-1}. The non-covalent interactions calculation have been done with NCIplot program[6] and all 3D representations were created using the CYLview software.[7]

[2] M. J. Frisch, G. W. Trucks, H. B. Schlegel, G. E. Scuseria, M. A. Robb, J. R. Cheeseman, G. Scalmani, V. Barone, B. Mennucci, G. A. Petersson, H. Nakatsuji, M. Caricato, X. Li, H. P. Hratchian, A. F. Izmaylov, J. Bloino, G. Zheng, J. L. Sonnenberg, M. Hada, M. Ehara, K. Toyota, R. Fukuda, J. Hasegawa, M. Ishida, T. Nakajima, Y. Honda, O. Kitao, H. Nakai, T. Vreven, J. A. Montgomery, J. E. Peralta, F. Ogliaro, M. Bearpark, J. J. Heyd, E. Brothers, K. N. Kudin, V. N. Staroverov, R. Kobayashi, J. Normand, K. Raghavachari, A. Rendell, J. C. Burant, S. S. Iyengar, J. Tomasi, M. Cossi, N. Rega, J. M. Millam, M. Klene, J. E. Knox, J. B. Cross, V. Bakken, C. Adamo, J. Jaramillo, R. Gomperts, R. E. Stratmann, O. Yazyev, A. J. Austin, R. Cammi, C. Pomelli, J. W. Ochterski, R. L. Martin, K. Morokuma, V. G. Zakrzewski, G. A. Voth, P. Salvador, J. J. Dannenberg, S. Dapprich, A. D. Daniels, Ö. Farkas, J. B. Foresman, J. V. Ortiz, J. Cioslowski, D. J. Fox, in *Gaussian 09, Revision B.01, Gaussian, Inc., Wallingford CT*, Wallingford CT, 2009.
[3] J.-D. Chai, M. Head-Gordon, *PhysChemChemPhys*, 2008, **10**, 6615-6620.
[4] G. Luchini, J. Alegre-Requena, I. Funes-Ardoiz, R. Paton, *F1000Research*, 2020, **9**.
[5] S. Grimme, *Chem. Eur. J.*, 2012, **18**, 9955-9964.
[6] J. Contreras-García, E. R. Johnson, S. Keinan, R. Chaudret, J.-P. Piquemal, D. N. Beratan, W. Yang, *J. Chem. Theory Comput.*, 2011, **7**, 625-632.
[7] C. Y. Legault, 1.0b ed., Université de Sherbrooke, Québec, Montreal, Canada, 2009, p. http://www.cylview.org.

5.2. Concerted Mechanism with Achiral Catalyst

Table S1. Calculated activation energies (in toluene, kcal/mol) for the **DMP**-promoted (3+2)-cycloaddition by a concerted mechanism [wB97XD/DEF2TZVPP(SMD,toluene)//wB97XD/DEF2SVPP(SMD,toluene)].[a]

Structures	ΔG (toluene)	*Structures*	ΔG (toluene)
**aDC+7a**	*0.0*		
**aTC$_{exo}$**[b]	9.4	**aTC$_{endo}$**[b]	---
**aTS$_{exo}$**	15.9	**aTS$_{endo}$**	---
***rac*-4l**	–19.8	***rac-diast*-4l**	–17.4

[a] The activation energies refer to the differences between the energies of the structure and the sum of energies of the enamine **7a** and the starting complex **aDC**. [b] TC, ternary complex between **7a** and **aDC**.

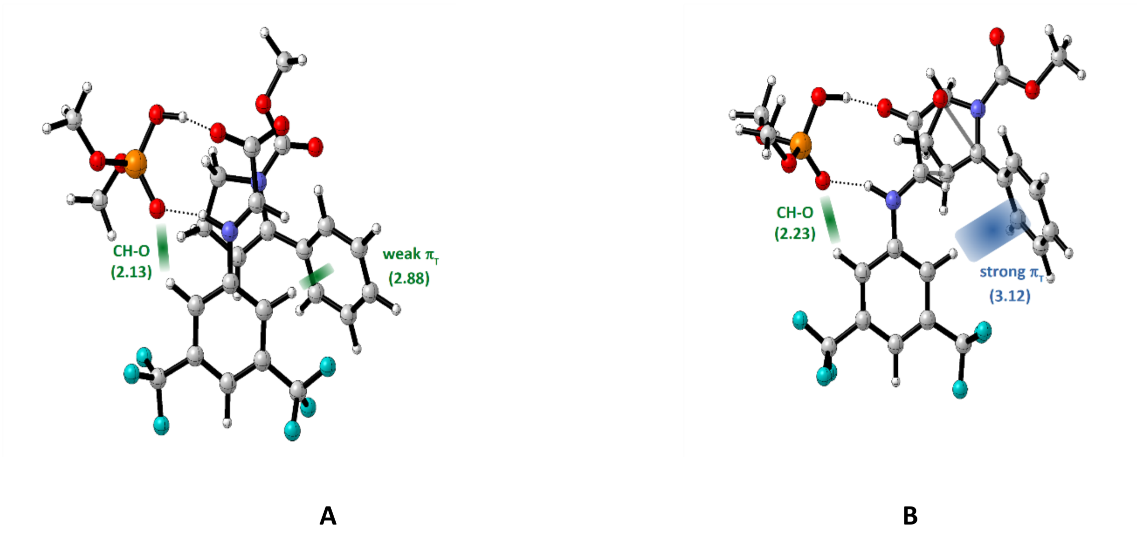

Figure S1. Representation of ternary complex (**aTC$_{exo}$**, **A**) and transition structure (**aTS$_{exo}$**, **B**) with distances indicated in Å, computed at the [wB97XD/DEF2TZVPP(SMD,toluene)//wB97XD/DEF2SVPP(SMD,toluene)] level.

5.3. Stepwise Mechanism with Achiral Catalyst

Table S2. Calculated relative free energies (kcal/mol) for the **DMP**-promoted (3+2)-cycloaddition by a stepwise mechanism at the wB97XD/DEF2TZVPP(SMD,toluene)//wB97XD/DEF2SVPP(SMD,toluene) level of theory.[a]

***anti*-Approach**		***syn*-Approach**	
Structures	**ΔG (toluene)**	***Structures***	**ΔG (toluene)**
**aTC$_{anti}$**[b]	10.4	**aTC$_{syn}$**[b]	10.3
**aTS-I$_{anti}$**	15.8	**aTSI$_{syn}$**	15.9
**aInt-I$_{anti}$**	0.4	**aInt-I$_{syn}$**	5.2
**aInt-II$_{anti}$**	----	**aInt-II$_{syn}$**	7.3
**aTS-II$_{anti}$**	----	**aTS-II$_{syn}$**	6.7
***rac*-4l**	–19.8	***rac-diast*-4l**	–17.4

[a] The activation energies refer to the differences between the energies of the structure and the sum of energies of the enamine **7a** and the starting complex **aDC**. [b] TC, ternary complex between **7a** and **aDC**.

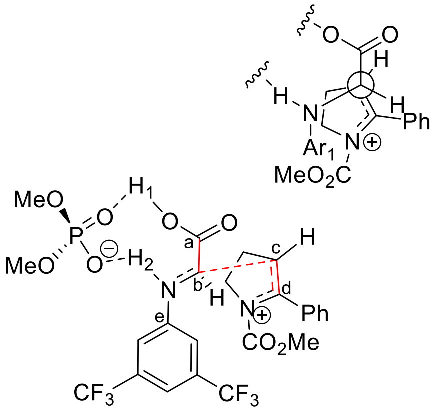

aTS-I$_{syn}$

$C_aC_bC_cC_d$ -153.7º
C_bC_c, 2.18 Å
CH-O, 2.19 Å
H_1-O, 1.49 Å
H_2-O, 1.77 Å

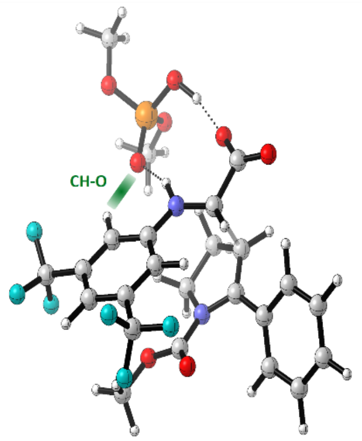

A

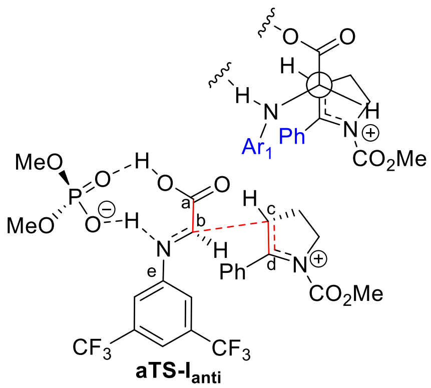

aTS-I$_{anti}$

$C_aC_bC_cC_d$ -174.6º
C_bC_c, 2.23 Å
p-p (PD), 3.58 Å
CH-O, 2.23 Å
H_1-O, 1.36 Å
H_2-O, 1.69 Å

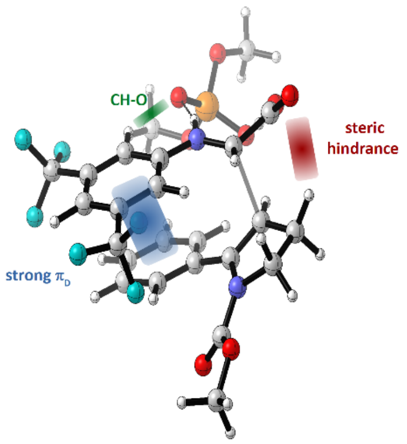

B

Figure S2. Structural representation of computed transition states ***a*TS-I$_{syn}$** (**A**) and ***a*TS-I$_{anti}$** (**B**) for the first step of the **DMP**-promoted (3+2)-cycloadition by a stepwise mechanism. Distances are indicated in Å. The PD π interaction (blue) and the methylene steric distortion (red) are also indicated [wB97XD/DEF2TZVPP(SMD,toluene)//wB97XD/DEF2SVPP(SMD,toluene)].

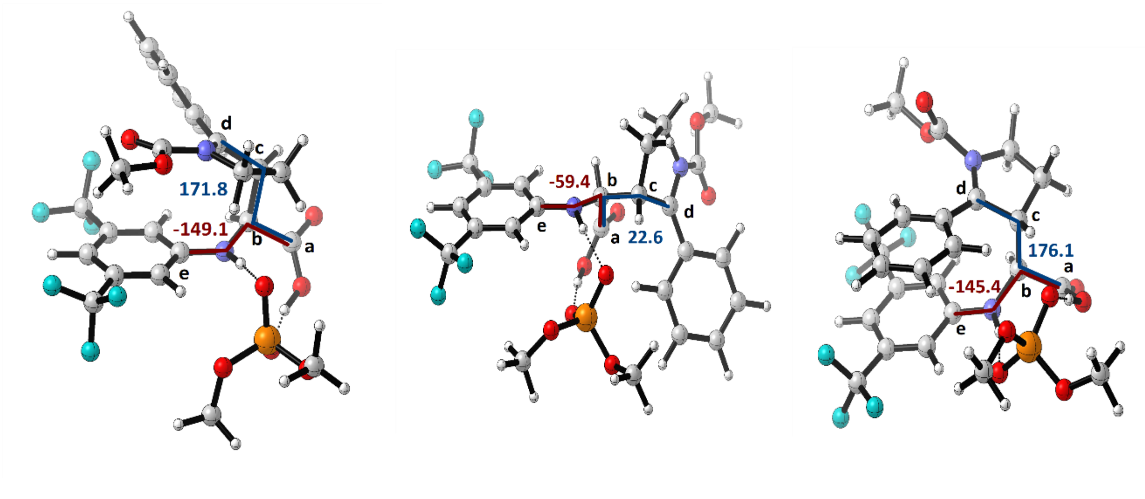

*a*Int-I$_{syn}$ *a*Int-II$_{syn}$ *a*Int-I$_{anti}$

Figure S3. Structural representation of computed intermediates ***a*Int-I$_{syn}$**, ***a*Int-I$_{anti}$** and ***a*Int-II$_{syn}$**, with selected dihedral angles in degrees ($C_aC_bC_cC_d$ angle in blue color; $C_aC_bNC_e$ angle in red color) [wB97XD/DEF2TZVPP(SMD,toluene)//wB97XD/DEF2SVPP(SMD,toluene)].

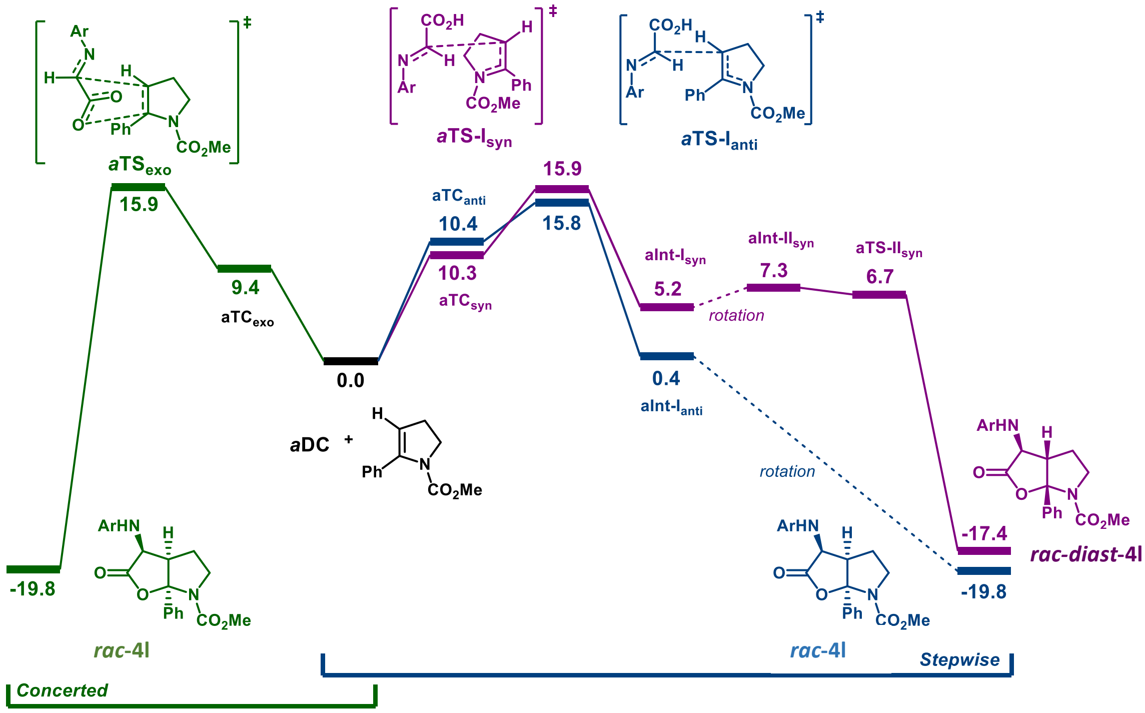

Figure S4. Computed reaction free energy profiles for the **DMP**-promoted (3+2)-cycloaddition (Ar = 3,5-$(CF_3)_2$-C_6H_3) at the wB97XD/DEF2TZVPP(SMD,toluene)//wB97XD/DEF2SVPP(SMD,toluene) level. The concerted (green colour) and stepwise (*anti* in blue and *syn* in purple colour) mechanisms are shown. The energies are in kcal/mol. **aTC** has been removed for clarity.

5.4. Concerted Mechanism with Chiral Catalyst

Table 3. Activation energies (kcal/mol) for the **CPA$_F$**-promoted concerted (3+2)-cycloaddition computed at the wB97XD/DEF2TZVPP(SMD,toluene)//wB97XD/DEF2SVPP(SMD, toluene) level.[a]

Re-imine		*Si-imine*	
Re-**cDC+7a**	2.7	*Si*-**cDC+7a**	*0.0*
(ReRe)-***c*TC**	8.2	*(SiSi)*-***c*TC**	6.4
(ReRe)-***c*TS**	22.5	*(SiSi)*-***c*TS**	16.2
***ent-diast*-4l**	–17.5	***diast*-4l**	–19.6
(SiRe)-***c*TC**	4.5	*(ReSi)*-***c*TC**	4.2
(SiRe)-***c*TS**	19.1	*(ReSi)*-***c*TS**	13.4
***ent*-4l**	–17.5	**4l**	–22.2

[a] The activation energies refer to the differences between the energies of the structure and the sum of energies of the enamine **7a** and the starting complex **cDC**.

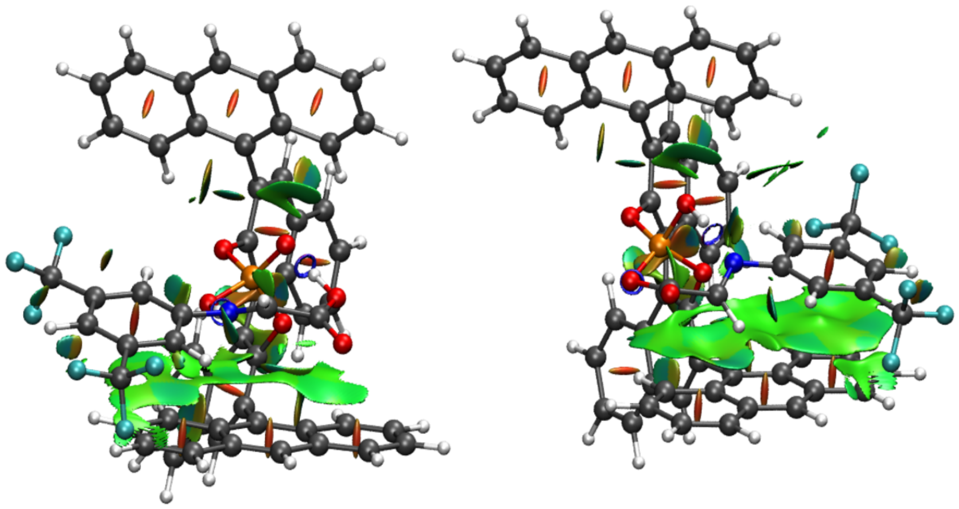

Re-**cDC** *Si*-**cDC**

Figure S5. Representation of computed non-covalent interactions (NCI) of binary complexes *Si*-***c*DC** and *Re*-***c*DC** (wB97XD/DEF2SVPP(SMD,toluene)). The second density Hessian eigenvalue (λ_H) is represented from an attractive strong interaction (blue, λ_H= 0.04) to a strong repulsive interaction (red, λ_H= -0.04), green means a weak interaction with λ_H close to 0.

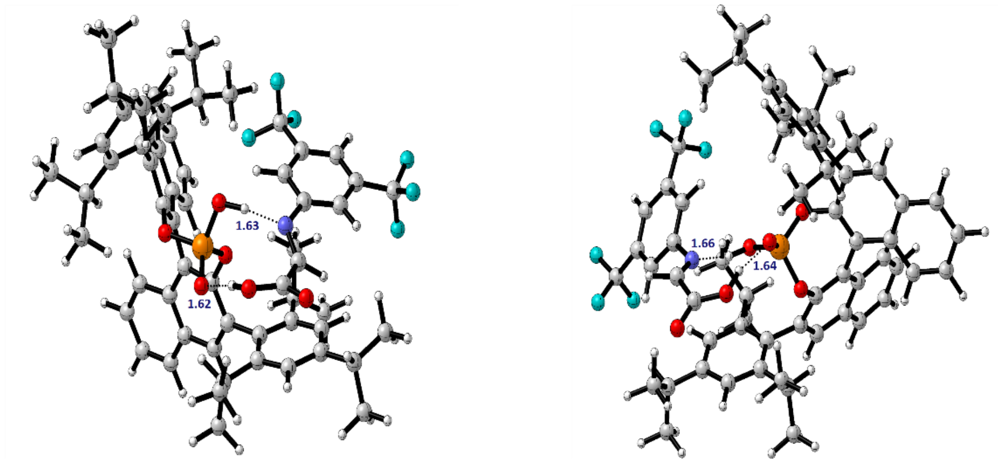

Si-***c*DC_TRIP** *Re*-***c*DC_TRIP**

Figure S6. Representation of binary complexes *Si*-***c*DC** and *Re*-***c*DC** for the enantiopure (*R*)-3,3′-bis(9-2,4,6-triisopropylphenyl)-1,1′-binaphthyl-2,2′-diyl hydrogenphosphate (**CPA$_B$**) ligand [wB97XD/DEF2TZVPP(SMD,toluene)//wB97XD/DEF2SVPP(SMD,toluene)]. Bond distances are indicated in Å.

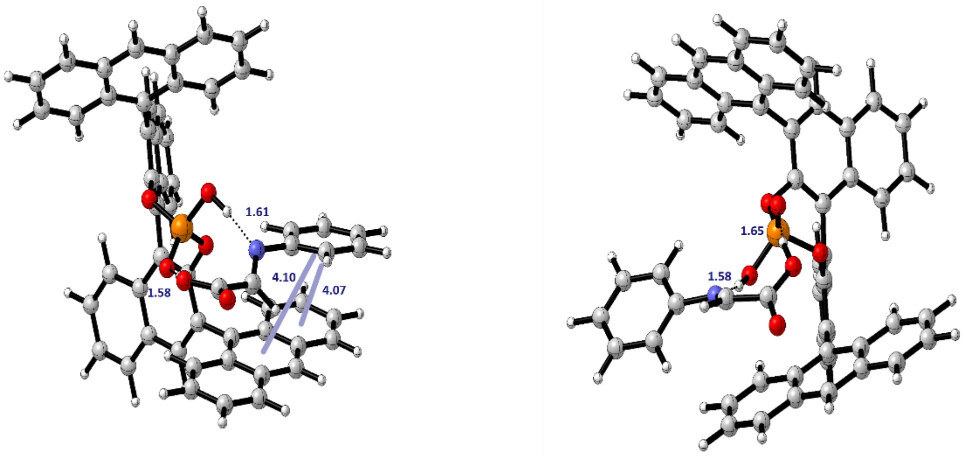

Si-***c*DC_Ph** *Re*-***c*DC_Ph**

Figure S7. Representation of computed binary complexes *Si*-***c*DC** and *Re*-***c*DC** between (*R*)-3,3′-bis(9-anthracenyl)-1,1′-binaphthyl-2,2′-diyl hydrogenphosphate) and the imine formed from aniline and glyoxylic acid [wB97XD/DEF2TZVPP(SMD,toluene)//wB97XD/DEF2SVPP(SMD,toluene)]. Distances are indicated in Å.

Table S4. Calculated relative free energies (kcal/mol) for the different binary chiral complexes and selected structural parameters, specifically the ones corresponding to the H bonds of the bifunctional activation mode and the ones corresponding to the non-covalent π interactions at the wB97XD/DEF2TZVPP(SMD,toluene)//wB97XD/DEF2SVPP(SMD,toluene) level of theory.

	ΔG (in kcal/mol)	r(H$_1$-O) (in Å)	r(H$_2$-N) (in Å)	r1(π-π) (in Å)	r1(π-π) (in Å)
**Anthracenyl_CF$_3$**					
Si-**cDC**	0.0	1.59	1.64	3.68	3.81
Re-**cDC**	2.7	1.59	1.65	3.95	-
**Anthracenyl_Ph**					
Si-**cDC_Ph**	0.0	1.58	1.61	4.07	4.10
Re-**cDC_Ph**	0.7	1.65	1.58	-	-
**TRIP**					
Si-**cDC_TRIP**	0.0	1.62	1.63	-	-
Re-**cDC_TRIP**	1.8	1.64	1.66	-	-

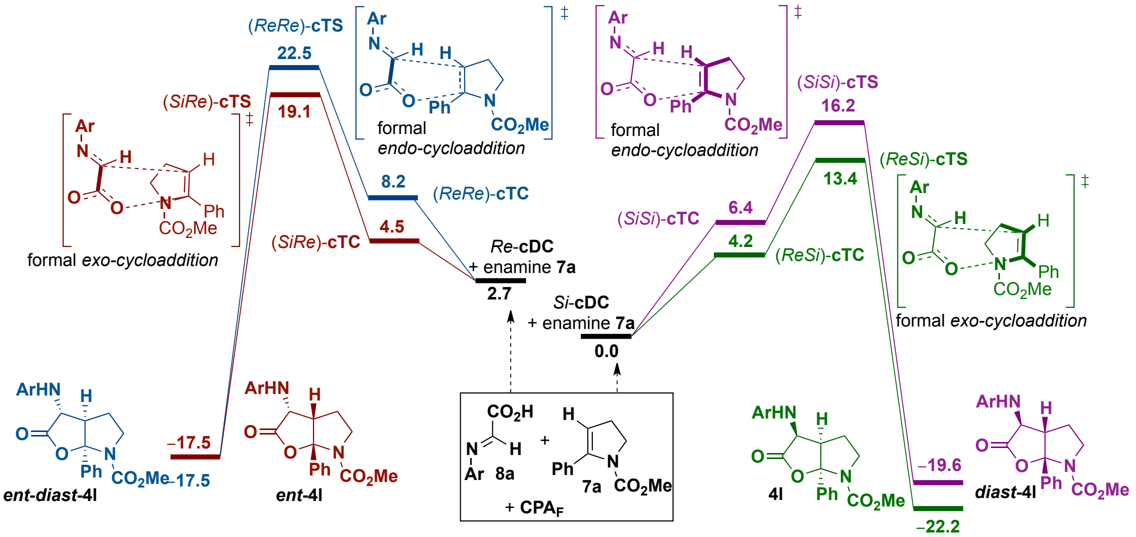

Figure S8. Reaction free energy profiles for the concerted pathway of the (3+2)-cycloaddition (Ar = 3,5-(CF$_3$)$_2$-C$_6$H$_3$) catalysed by **CPA$_F$** computed at the wB97XD/DEF2TZVPP(SMD,toluene)//wB97XD/DEF2SVPP(SMD,toluene) level. The *ReSi* (green), *SiRe* (red), *ReRe* (blue) and *SiSi* (purple) paths are shown. The energies are in kcal/mol. **CPA$_F$** has been removed for clarity.

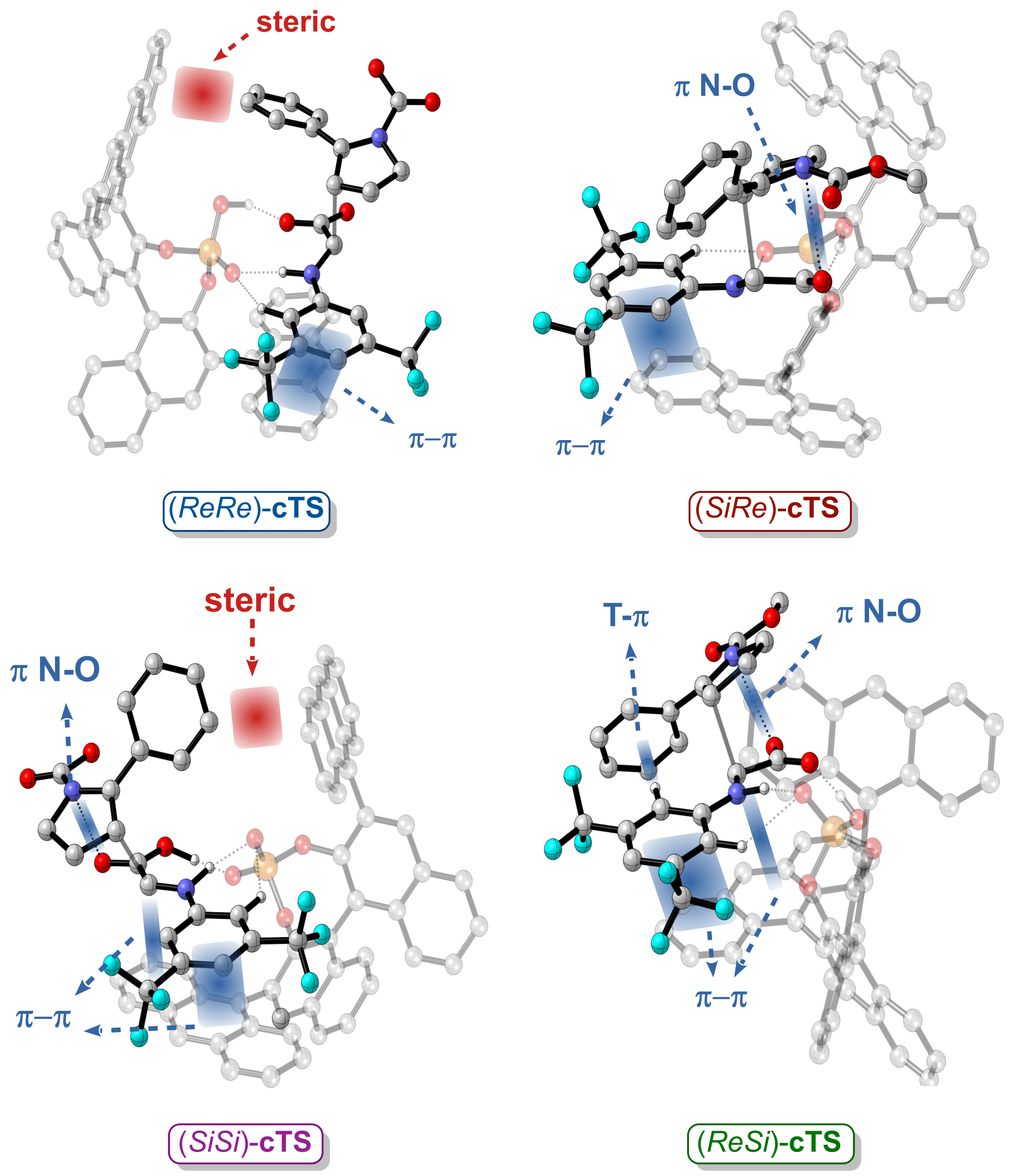

Figure S9. Representation of computed transition states for the four approaches (irrelevant hydrogen atoms have been removed for clarity) regarding the concerted pathway of the [3+2]-cycloaddition catalysed by **CPA$_F$** calculated at the wB97XD/DEF2TZVPP(SMD,toluene)//wB97XD/DEF2SVPP(SMD,toluene) level.

5.5. Activation Strain Model

Table S5. Strain interaction energy details computed at the ωB97XD/DEF2TZVPP(SMD,toluene) level of theory. Initially distorted energies were calculated using the *Si*-**cDC** and **7** as energy reference. **$\Delta\Delta E^{\ddagger}$** is the electronic activation energy of each TS, **E_{strain}** is the single point energy calculated for each fragment with the structure of the TS, **$\Delta\Delta E_{strain}$** is the strain energy difference setting the lowest energy TS as reference, **ΔE_{strain}** is the energy difference between the distorted and not distorted energy calculation, **ΔE_{int}** and **$\Delta\Delta E_{int}$** are the relative interaction energies (referred to the reactant or to the lower energy transition state). Superscript **c** means that the difference energy value between *Re*-**cDC** and *Si*-**cDC** was discarded to account for real distortion (and not the difference in starting point stability).

TS	$\Delta\Delta E^{\ddagger}$	E_{strain} *(cDC)*	$\Delta\Delta E_{strain}$ *(cDC)*	$\Delta\Delta E^{c}_{strain}$ *(cDC)*	E_{strain} *(7)*	$\Delta\Delta E_{strain}$ *(7)*	ΔE_{strain}	$\Delta\Delta E_{strain}$	$\Delta\Delta E^{c}_{strain}$	ΔE_{int}	$\Delta\Delta E_{int}$
ReRe	9.51	-2307740.53	5.65	1.40	-420645.52	1.52	23.96	7.17	2.92	-17.87	2.34
SiRe	6.33	-2307741.75	4.43	0.18	-420646.58	0.46	21.67	4.89	0.64	-18.77	1.45
SiSi	2.62	-2307745.19	0.99	0.99	-420646.28	0.76	18.54	1.76	1.76	-19.35	0.87
ReSi	0.00	-2307746.18	0.00	0.00	-420647.04	0.00	16.79	0.00	0.00	-20.21	0.00

5.6. QTAIM Analysis

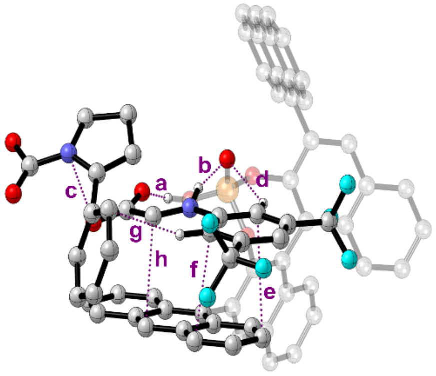

Figure S10. Representation of computed BCPs for *ReSi*-**cTS** using QTAIM at the (wB97XD/DEF2SVPP(SMD,toluene)) level of theory.

Table S6. List of BCPs obtained for *ReSi*-**cTS** using QTAIM at the (wB97XD/DEF2SVPP(SMD,toluene)) level of theory and its relation with non-covalent interactions. For each interaction the distance between interacting atoms is specified also, the electron density, the Laplacian of the electron density, the potential energy and the kinetic energy at the corresponding BCP.

BCP	*Interaction*	*distance (Å)*	*ρ x 10^{-2} (a.u.)*	*$\nabla^2\rho$ x 10^{-2} (a.u.)*	*V x 10^{-2} (a.u.)*	*K x 10^{-2} (a.u.)*
a	Imine O ··· H-O Catalyst	1.49	7.1837	18.3059	-7.7941	1.6088
b	Imine N-H ··· O Catalyst	1.80	3.8289	12.7905	-3.2654	0.0245
c	Enamine N(π) ··· O(π) Imine	2.83	1.3303	4.3745	-0.9672	-0.0632
d	Imine C-H ··· O Catalyst	2.37	1.2770	4.2367	-0.9953	-0.0319
e	Imine π ··· π Catalyst	3.57	0.5354	1.2416	-0.2302	-0.0401
f	Imine π ··· π Catalyst	3.40	0.6786	1.6097	-0.2887	-0.0569
g	Imine π ··· C-H Enamine	2.81	0.6137	1.8955	-0.3166	-0.0786
h	Imine N=C (π) ··· π Catalyst	3.34	0.5979	1.9584	-0.2634	-0.1131

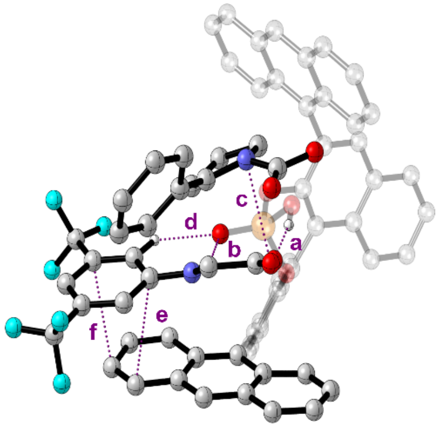

Figure S11. Representation of computed BCPs for *SiRe*-**cTS** using QTAIM at the (wB97XD/DEF2SVPP(SMD,toluene)) level of theory.

Table S7. List of BCPs obtained for *SiRe*-**cTS** using QTAIM at the (wB97XD/DEF2SVPP(SMD,toluene)) level of theory and its relation with non-covalent interactions. For each interaction the distance between interacting atoms is specified also, the electron density, the Laplacian of the electron density, the potential energy and the kinetic energy at the corresponding BCP.

BCP	*Interaction*	*distance (Å)*	*ρ x 10^{-2} (a.u.)*	*$\nabla^2\rho$ x 10^{-2} (a.u.)*	*V x 10^{-2} (a.u.)*	*K x 10^{-2} (a.u.)*
a	Imine O ··· H-O Catalyst	1.45	7.9559	17.7475	-9.0447	2.3039
b	Imine N-H ··· O Catalyst	1.83	3.4898	11.7379	-2.9459	0.0057
c	Enamine N(π) ··· O(π) Imine	2.98	1.2131	4.0664	-0.8698	-0.0734
d	Imine C-H ··· O Catalyst	2.22	1.4939	5.4450	-1.1968	-0.0822
e	Imine π ··· π Catalyst	3.42	0.6114	1.5941	-0.2793	-0.0596
f	Imine π ··· π Catalyst	3.28	0.7254	1.9684	-0.3303	-0.0809

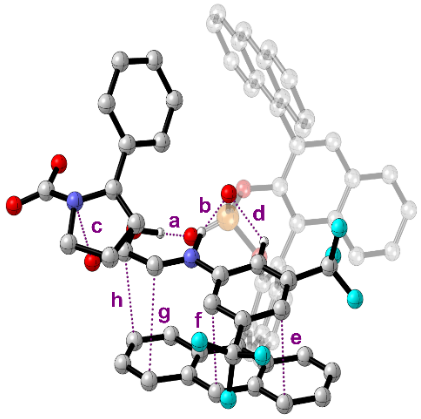

Figure S12. Representation of computed BCPs for *SiSi*-**cTS** using QTAIM at the (wB97XD/DEF2SVPP(SMD,toluene)) level of theory.

Table S8. List of BCPs obtained for *SiSi*-**cTS** using QTAIM at the (wB97XD/DEF2SVPP(SMD,toluene)) level of theory and its relation with non-covalent interactions. For each interaction the distance between interacting atoms is specified also, the electron density, the Laplacian of the electron density, the potential energy and the kinetic energy at the corresponding BCP.

BCP	*Interaction*	*distance (Å)*	*ρ x 10^{-2} (a.u.)*	*$\nabla^2\rho$ x 10^{-2} (a.u.)*	*V x 10^{-2} (a.u.)*	*K x 10^{-2} (a.u.)*
a	Imine O ··· H-O Catalyst	1.40	9.3517	12.3350	-10.9556	3.9359
b	Imine N-H ··· O Catalyst	1.75	4.6289	14.8424	-3.9820	0.1357
c	Enamine N(π) ··· O(π) Imine	2.80	1.3641	4.7241	-1.0037	-0.0887
d	Imine C-H ··· O Catalyst	2.23	1.7205	5.4885	-1.3823	0.0051
e	Imine π ··· π Catalyst	3.32	0.6643	1.6937	-0.2869	-0.0682
f	Imine π ··· π Catalyst	3.27	0.7371	1.8708	-0.3249	-0.0714
g	Imine C=O (π) ··· π Catalyst	3.25	0.5955	2.0710	-0.2744	-0.1217
h	Imine N=C (π) ··· π Catalyst	2.87	0.6404	1.9785	-0.3035	-0.0955

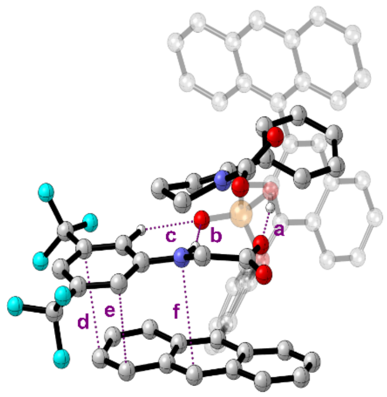

Figure S13. Representation of computed BCPs for *ReRe*-**cTS** using QTAIM at the (wB97XD/DEF2SVPP(SMD,toluene)) level of theory.

Table S9. List of BCPs obtained for *ReRe*-**cTS** using QTAIM at the (wB97XD/DEF2SVPP(SMD,toluene)) level of theory and its relation with non-covalent interactions. For each interaction the distance between interacting atoms is specified also, the electron density, the Laplacian of the electron density, the potential energy and the kinetic energy at the corresponding BCP.

BCP	*Interaction*	*distance (Å)*	*ρ x 10^{-2} (a.u.)*	*$\nabla^2\rho$ x 10^{-2} (a.u.)*	*V x 10^{-2} (a.u.)*	*K x 10^{-2} (a.u.)*
a	Imine O ··· H-O Catalyst	1.52	6.6063	19.1731	-7.0128	1.1097
b	Imine N-H ··· O Catalyst	1.82	3.5844	12.0191	-3.0356	0.0154
c	Imine C-H ··· O Catalyst	2.20	1.5707	5.7074	-1.2626	-0.0821
d	Imine π ··· π Catalyst	3.34	0.6798	1.8386	-0.3118	-0.0739
e	Imine π ··· π Catalyst	3.32	0.6998	1.8489	-0.3117	-0.0753
f	Imine N=C (π) ··· π Catalyst	3.45	0.5653	1.4976	-0.2999	-0.0372

5.5. XYZ Coordinates

aDC

Zero-point correction= 0.256672 (Hartree/Particle)
Thermal correction to Energy= 0.283160
Thermal correction to Enthalpy= 0.284104
Thermal correction to Gibbs Free Energy= 0.194717
Sum of electronic and zero-point Energies= -1909.093720
Sum of electronic and thermal Energies= -1909.067232
Sum of electronic and thermal Enthalpies= -1909.066288
Sum of electronic and thermal Free Energies= -1909.155675
Quasi-Harmonic Approximation corrected Free energy= -1909.148443
E(wB97XD/Def2TZVPP)= -1911.37705791

O	4.764387	-0.527866	0.612936
P	3.325032	-0.155074	0.062810
O	3.388949	0.819934	-1.065439
O	2.625857	-1.543348	-0.342634
O	2.486828	0.312249	1.314598
O	2.822384	3.271697	-0.500094
C	1.579779	3.656121	-0.387500
O	1.244900	4.791711	-0.165146
C	0.461566	2.646366	-0.519871
N	0.424362	1.562174	0.143212
C	-0.733898	0.750181	0.108844
C	-2.023308	1.292291	0.172263
C	-3.127116	0.448071	0.121719
C	-2.966323	-0.934469	0.006272
C	-1.681250	-1.463296	-0.036736
C	-0.564108	-0.631651	0.031519
H	1.690008	0.866755	1.017112
H	2.984602	2.306811	-0.760168
H	0.441960	-1.059486	0.007794
H	-3.838137	-1.590546	-0.039282
H	-2.160274	2.370028	0.284456
C	-1.449406	-2.950283	-0.155373
F	-0.776487	-3.423301	0.897599
F	-2.589282	-3.635233	-0.245390
F	-0.720747	-3.239791	-1.242164
C	-4.526170	1.008027	0.218572
F	-5.104134	0.672232	1.376575
F	-4.541806	2.339456	0.135994
F	-5.306545	0.534785	-0.757961
H	-0.382949	2.978879	-1.147317
C	2.919576	-2.182314	-1.581584
H	3.981334	-2.480176	-1.625958
H	2.285747	-3.078932	-1.630442
H	2.687322	-1.515211	-2.427871
C	4.957089	-1.420961	1.706570
H	4.449576	-2.383266	1.523356
H	6.040546	-1.587580	1.789214
H	4.580245	-0.973252	2.640654

aInt-I_anti

Zero-point correction= 0.495260 (Hartree/Particle)
Thermal correction to Energy= 0.534870
Thermal correction to Enthalpy= 0.535814
Thermal correction to Gibbs Free Energy= 0.419449
Sum of electronic and zero-point Energies= -2578.497562
Sum of electronic and thermal Energies= -2578.457953
Sum of electronic and thermal Enthalpies= -2578.457009
Sum of electronic and thermal Free Energies= -2578.573373
Quasi-Harmonic Approximation corrected Free energy= -2578.563831
E(wB97XD/Def2TZVPP)= -2581.75990355

O	-5.482224	0.113210	-0.656249
P	-4.012651	0.067482	0.023837
O	-4.289607	0.600070	1.540189
O	-3.564633	-1.380626	0.199558
O	-3.116698	0.997978	-0.738213
O	-2.886311	-2.561453	-1.926000
C	-1.630067	-2.571028	-2.286030
O	-1.240474	-3.136193	-3.279091
C	-0.606019	-1.849096	-1.376739
N	-0.780830	-0.430646	-1.405467
C	0.254105	0.465456	-1.316480
C	1.605990	0.111123	-1.461311
C	2.608042	1.064399	-1.276806
C	2.308965	2.382161	-0.958478
C	0.960030	2.735241	-0.830647
C	-0.053004	1.811380	-1.009786
C	4.049630	0.632804	-1.299064
C	-0.636684	-2.504266	0.059702
C	0.409669	-1.893976	0.942748
N	1.458860	-2.684988	0.985540
C	1.298810	-3.922541	0.189621
C	-0.214934	-3.981326	0.005156
C	0.305125	-0.596386	1.583349
C	1.436157	0.215729	1.808141
C	1.288203	1.483453	2.344448
C	0.014084	1.957394	2.673976
C	-1.109605	1.163910	2.456452
C	-0.972629	-0.103755	1.901476
C	2.669048	-2.498779	1.753332
O	3.682315	-2.474376	0.923652
C	4.990825	-2.337690	1.489012
O	2.682046	-2.436290	2.942523
H	-1.715355	-0.031152	-1.221330
H	-3.138159	-2.067527	-1.054624
H	-0.493759	-4.477443	-0.935775
H	1.856520	-3.813173	-0.754731
H	-0.678873	-4.535615	0.835825
H	1.716711	-4.770629	0.751262
H	-1.643418	-2.339933	0.470848
H	5.672494	-2.250133	0.634349
H	5.040842	-1.435562	2.116527
H	5.233327	-3.228548	2.089375
H	0.354442	-2.102385	-1.840809
H	1.896146	-0.910720	-1.705325
H	3.099479	3.118797	-0.800144
C	0.632542	4.149358	-0.425802
H	-1.103511	2.082616	-0.876750
H	-1.866802	-0.704317	1.723052
H	-2.108182	1.531077	2.699333
H	-0.099317	2.965053	3.082716
H	2.165344	2.120107	2.479590
H	2.429538	-0.098275	1.482872
F	4.849189	1.548633	-1.846490
F	4.508966	0.430185	-0.042854
F	4.239504	-0.513708	-1.957402
F	1.059901	5.037427	-1.330643
F	-0.673124	4.349494	-0.253453
F	1.235423	4.468234	0.734115
C	-4.727332	1.930987	1.721069
H	-5.727183	2.087783	1.275909
H	-4.791080	2.112986	2.805802

H	-4.023683	2.652285	1.268118
C	-6.481892	-0.792545	-0.239322
H	-6.218841	-1.830210	-0.509735
H	-6.648202	-0.739845	0.852607
H	-7.413327	-0.511007	-0.755545

aInt-II_syn

Zero-point correction= 0.495342
(Hartree/Particle)
Thermal correction to Energy= 0.534827
Thermal correction to Enthalpy= 0.535772
Thermal correction to Gibbs Free Energy= 0.418330
Sum of electronic and zero-point Energies= -
2578.485379
Sum of electronic and thermal Energies= -
2578.445894
Sum of electronic and thermal Enthalpies= -
2578.444949
Sum of electronic and thermal Free Energies= -
2578.562391
Quasi-Harmonic Approximation corrected Free energy= -2578.552076
E(wB97XD/Def2TZVPP)= -2581.74855945

O	-0.179738	4.209658	-0.595805
P	0.592515	2.906512	0.001353
O	0.680663	1.924863	-1.148282
O	2.076479	3.465565	0.349193
O	-0.027762	2.463276	1.301797
O	-0.369402	-0.231477	-1.774117
C	-1.049682	-0.954645	-0.942220
O	-1.927537	-1.721243	-1.289632
C	-0.752129	-0.886990	0.579332
N	0.471593	-0.282842	1.002365
C	1.710906	-0.735442	0.621241
C	1.925444	-1.987578	0.029461
C	3.217497	-2.386025	-0.325392
C	4.314470	-1.561390	-0.122315
C	4.094586	-0.305268	0.456809
C	2.827130	0.104106	0.830610
H	0.406157	0.714308	1.250149
H	0.147374	0.603219	-1.411568
H	2.676539	1.092508	1.271067
H	5.317608	-1.877810	-0.411235
H	1.090567	-2.662594	-0.171947
H	-0.756520	-1.954342	0.860754
C	5.263626	0.632228	0.616259
F	4.999209	1.646770	1.443062
F	6.343907	0.001226	1.093712
F	5.622956	1.169706	-0.559337
C	3.389563	-3.758023	-0.924482
F	4.641270	-3.989490	-1.326918
F	3.076890	-4.718815	-0.040566
F	2.591020	-3.941575	-1.982540
C	-5.106031	1.160176	-0.204582
C	-3.729388	0.856216	-0.118930
C	-2.795765	1.752573	-0.681640
C	-3.221468	2.882532	-1.363442
C	-4.585863	3.159112	-1.454089
C	-5.523347	2.307090	-0.861087
C	-3.232779	-0.244636	0.669078
C	-1.906434	-0.200394	1.387589
C	-2.229441	-1.016680	2.643820
C	-3.175224	-2.083700	2.104963
N	-3.866518	-1.363602	1.005550
C	-4.858644	-2.032512	0.213218
O	-5.378564	-1.578664	-0.758485
O	-5.058473	-3.227105	0.726893
C	-5.957280	-4.082583	0.017624
H	-2.740712	-0.388435	3.391156
H	-3.919693	-2.434587	2.831137
H	-1.324601	-1.438162	3.104402
H	-2.653977	-2.957289	1.683161
H	-1.609574	0.838046	1.592745
H	-6.959612	-3.629028	-0.028299
H	-5.581157	-4.265058	-1.000899
H	-5.985201	-5.018854	0.589717
H	-1.726362	1.562401	-0.615646
H	-2.473861	3.548374	-1.800235
H	-4.925366	4.056702	-1.979048
H	-6.589091	2.543692	-0.910719
H	-5.847031	0.522739	0.276408
C	3.018578	3.678433	-0.683075
H	3.245121	2.740810	-1.218119
H	2.652067	4.425130	-1.411486
H	3.937436	4.059444	-0.211479
C	-0.557848	5.250610	0.278641
H	-1.273918	4.893937	1.040002
H	0.319954	5.684848	0.791681
H	-1.034857	6.034789	-0.330941

aInt-I_syn

Zero-point correction= 0.494789
(Hartree/Particle)
Thermal correction to Energy= 0.534123
Thermal correction to Enthalpy= 0.535068
Thermal correction to Gibbs Free Energy= 0.418097
Sum of electronic and zero-point Energies= -
2578.489393
Sum of electronic and thermal Energies= -
2578.450059
Sum of electronic and thermal Enthalpies= -
2578.449115
Sum of electronic and thermal Free Energies= -
2578.566086
Quasi-Harmonic Approximation corrected Free energy= -2578.555566
E(wB97XD/Def2TZVPP)= -2581.75187629

O	-5.440648	-1.444367	-0.035526
P	-3.958537	-0.865477	-0.337366
O	-3.526567	-1.412523	-1.680400
O	-4.196554	0.742540	-0.451967
O	-3.033013	-1.064079	0.841133
O	-1.938642	-3.314516	-1.817182
C	-0.752050	-3.502982	-1.339261
O	-0.197308	-4.579999	-1.350885
C	0.056282	-2.299887	-0.772865
N	-0.667336	-1.088312	-0.631627
C	-0.121553	0.166777	-0.747660
C	1.146629	0.415131	-1.294278
C	1.662448	1.710452	-1.331363
C	0.940535	2.795012	-0.852879
C	-0.331045	2.549325	-0.323831
C	-0.856854	1.268823	-0.256923
H	-1.545133	-1.135688	-0.089543
H	-2.488198	-2.418463	-1.738162
H	-1.848615	1.101010	0.170750
H	1.353962	3.803244	-0.879766
H	1.755263	-0.398720	-1.690799
H	0.851486	-2.165313	-1.519530
C	-1.179261	3.697150	0.163214
F	-1.911074	3.359933	1.231581
F	-0.434712	4.754017	0.516498
F	-2.032948	4.117540	-0.776614
C	3.063039	1.882337	-1.860079
F	3.439163	3.161566	-1.910233
F	3.955864	1.235670	-1.097335
F	3.188035	1.381387	-3.096963
C	4.039289	-1.177893	1.569066
C	3.059173	-1.715417	0.711580
C	3.470987	-2.324385	-0.493754
C	4.807388	-2.316115	-0.865225
C	5.762149	-1.748681	-0.019003

C	5.378314	-1.202472	1.206370
C	1.658147	-1.768547	1.086553
C	0.705227	-2.800376	0.581510
C	-0.340638	-2.884884	1.703528
C	-0.296843	-1.509887	2.366055
N	1.017455	-0.963941	1.930895
C	1.349732	0.406003	2.219694
O	2.302873	0.979641	1.787093
O	0.409934	0.909295	2.984001
C	0.514722	2.300342	3.295360
H	-0.062391	-3.677714	2.414252
H	-0.302851	-1.549551	3.464483
H	-1.352128	-3.100308	1.326889
H	-1.112791	-0.852784	2.033697
H	1.195119	-3.756341	0.364184
H	1.354775	2.464110	3.988860
H	0.670416	2.887088	2.378365
H	-0.436232	2.569439	3.771218
H	2.746868	-2.798748	-1.158380
H	5.108266	-2.765364	-1.814610
H	6.816257	-1.748032	-0.309872
H	6.128704	-0.787525	1.883657
H	3.761037	-0.766268	2.538484
C	-4.805614	1.296228	-1.598564
H	-4.274686	0.992218	-2.517226
H	-5.864563	0.987812	-1.678908
H	-4.756781	2.391945	-1.496108
C	-6.076041	-1.132209	1.184716
H	-5.482435	-1.485682	2.046547
H	-6.239595	-0.043350	1.287127
H	-7.053054	-1.641118	1.185703

aTC_anti

Zero-point correction= 0.491272
(Hartree/Particle)
Thermal correction to Energy= 0.532242
Thermal correction to Enthalpy= 0.533186
Thermal correction to Gibbs Free Energy= 0.412125
Sum of electronic and zero-point Energies= -
2578.475343
Sum of electronic and thermal Energies= -
2578.434374
Sum of electronic and thermal Enthalpies= -
2578.433430
Sum of electronic and thermal Free Energies= -
2578.554491
Quasi-Harmonic Approximation corrected Free energy= -2578.543421
E(wB97XD/Def2TZVPP)= -2581.73814657

O	-5.621664	-0.583167	-0.580260
P	-4.212925	-0.380497	0.145915
O	-4.532485	0.399053	1.505119
O	-3.708421	-1.759848	0.683119
O	-3.298470	0.349848	-0.789218
O	-2.659252	-3.030138	-1.181581
C	-1.772876	-2.883100	-2.053638
O	-1.598286	-3.464069	-3.116052
C	-0.693271	-1.823295	-1.847962
N	-0.943453	-0.601072	-1.534096
C	0.005262	0.450470	-1.417040
C	1.369825	0.243858	-1.610652
C	2.251714	1.294370	-1.392053
C	1.785619	2.550272	-1.004991
C	0.418079	2.742088	-0.829993
C	-0.479247	1.698416	-1.031014
C	3.739281	1.050010	-1.466803
C	0.497326	-2.950241	0.488293
C	1.181473	-1.913215	1.015412
N	2.569088	-2.060301	0.729814
C	2.726694	-3.124453	-0.276492
C	1.426087	-3.935714	-0.166868
C	0.595363	-0.712050	1.647896
C	1.295778	0.498584	1.756996
C	0.676221	1.637688	2.261991
C	-0.659559	1.598598	2.660294
C	-1.368177	0.401737	2.558941
C	-0.745613	-0.741233	2.063586
C	3.590448	-1.707394	1.581809
O	4.775209	-2.010544	1.033200
C	5.920338	-1.660507	1.790048
O	3.464618	-1.188291	2.663998
H	-1.935688	-0.319348	-1.268470
H	-3.223401	-2.317009	-0.053617
H	1.066201	-4.280819	-1.150433
H	2.842100	-2.674298	-1.279454
H	1.552689	-4.838710	0.460445
H	3.629613	-3.712298	-0.073684
H	-0.584575	-3.074992	0.534973
H	6.784769	-1.987761	1.195028
H	5.966850	-0.571100	1.949946
H	5.922303	-2.169836	2.767685
H	0.323161	-2.106070	-2.131121
H	1.764527	-0.729287	-1.902144
H	2.487436	3.366964	-0.820544
C	-0.093093	4.096546	-0.401900
H	-1.550855	1.828405	-0.862953
H	-1.321190	-1.667477	2.008497
H	-2.415469	0.346977	2.865500
H	-1.144879	2.497845	3.050583
H	1.242924	2.570197	2.332579
H	2.333063	0.565759	1.434052
F	4.374893	2.048892	-2.081488
F	4.268787	0.949712	-0.238040
F	4.032049	-0.078237	-2.115024
F	-0.203698	4.928867	-1.441906
F	-1.295714	4.016887	0.172044
F	0.733570	4.674767	0.475674
C	-4.764264	1.801725	1.488380
H	-5.580999	2.063094	0.793053
H	-5.056882	2.085038	2.509966
H	-3.849579	2.348082	1.203291
C	-6.643386	-1.391315	-0.010862
H	-6.354034	-2.455096	-0.031959
H	-6.859701	-1.087899	1.028199
H	-7.543070	-1.245109	-0.626362

aTC_exo

Zero-point correction= 0.490516
(Hartree/Particle)
Thermal correction to Energy= 0.532062
Thermal correction to Enthalpy= 0.533006
Thermal correction to Gibbs Free Energy= 0.408673
Sum of electronic and zero-point Energies= -
2578.476868
Sum of electronic and thermal Energies= -
2578.435322
Sum of electronic and thermal Enthalpies= -
2578.434378
Sum of electronic and thermal Free Energies= -
2578.558712
Quasi-Harmonic Approximation corrected Free energy= -2578.546561
E(wB97XD/Def2TZVPP)= -2581.73735590

C	2.848214	2.011908	-1.617198
C	1.646749	2.319224	-0.961052
C	1.703098	3.076347	0.219754
C	2.930180	3.485070	0.739437
C	4.119140	3.161969	0.085465
C	4.072290	2.429155	-1.100251
C	0.383257	1.730903	-1.453659
C	0.263344	0.505855	-1.997409
C	-1.177490	0.130888	-2.198639

C	-1.936917	1.302065	-1.545123
N	-0.894452	2.291142	-1.226381
C	-1.190159	3.620189	-1.041518
O	-0.403196	4.535483	-1.069179
O	-2.502711	3.760756	-0.824451
C	-2.936149	5.048607	-0.427869
O	-2.330793	-1.479357	0.563346
P	-3.804046	-1.266018	0.415999
O	-4.583972	-2.432950	1.177453
O	-4.315486	-1.329251	-1.100788
O	-4.379751	0.108575	0.894854
O	-2.513280	1.210465	2.139695
C	-1.484271	1.917606	2.075469
O	-1.314882	3.129730	2.089098
C	-0.181689	1.116728	1.927676
N	-0.190097	-0.010129	1.314573
C	0.920389	-0.818203	0.941020
C	2.242478	-0.382444	1.063297
C	3.266024	-1.225020	0.647363
C	2.988719	-2.475531	0.088694
C	1.667085	-2.884216	-0.036209
C	0.626930	-2.066124	0.399269
C	4.710194	-0.796453	0.761128
H	-1.134303	-0.418336	1.056973
H	-3.698940	0.675551	1.413721
H	-1.420715	-0.821522	-1.699327
H	-2.456246	1.007081	-0.621263
H	-1.433100	0.012432	-3.267867
H	-2.689607	1.747418	-2.209750
H	1.100621	-0.164431	-2.194376
H	-4.028406	4.978641	-0.324969
H	-2.487576	5.320165	0.540914
H	-2.676042	5.807798	-1.183633
H	0.762391	1.542318	2.279932
H	2.485284	0.607305	1.453856
H	3.802703	-3.121123	-0.247481
C	1.308241	-4.213578	-0.654623
H	-0.417158	-2.378828	0.308877
H	0.785387	3.361646	0.738019
H	2.952167	4.074778	1.660744
H	5.080105	3.486081	0.494793
H	4.996274	2.178310	-1.628110
H	2.822436	1.446687	-2.552758
F	5.452848	-1.762960	1.311459
F	5.238199	-0.540045	-0.440222
F	4.855998	0.295758	1.509686
F	2.377814	-4.856483	-1.122819
F	0.707322	-5.016339	0.227488
F	0.457278	-4.049869	-1.675349
C	-5.998206	-2.410300	1.325742
H	-6.303198	-1.605311	2.014750
H	-6.501359	-2.269021	0.353231
H	-6.288670	-3.383745	1.747162
C	-4.064020	-2.482834	-1.891726
H	-4.665678	-3.339820	-1.541515
H	-4.355679	-2.232055	-2.921990
H	-2.995315	-2.757862	-1.873906

aTC_syn

Zero-point correction= 0.491776
(Hartree/Particle)
Thermal correction to Energy= 0.532693
Thermal correction to Enthalpy= 0.533638
Thermal correction to Gibbs Free Energy= 0.412812
Sum of electronic and zero-point Energies= -
2578.476919
Sum of electronic and thermal Energies= -
2578.436001
Sum of electronic and thermal Enthalpies= -
2578.435057
Sum of electronic and thermal Free Energies= -
2578.555883

Quasi-Harmonic Approximation corrected Free energy= -2578.545103
E(wB97XD/Def2TZVPP)= -2581.73867544

O	-4.638421	-0.961047	1.081880
P	-4.427986	-0.442643	-0.424243
O	-4.628828	-1.728421	-1.295085
O	-5.676774	0.493518	-0.741783
O	-3.155616	0.325131	-0.596461
O	-2.293779	-2.611397	-1.266849
C	-1.147432	-2.787990	-1.738237
O	-0.654504	-3.725299	-2.350913
C	-0.191178	-1.608144	-1.505077
N	-0.651400	-0.436547	-1.241837
C	0.100377	0.759631	-1.078398
C	1.392019	0.893202	-1.590247
C	2.083157	2.077291	-1.375600
C	1.506523	3.123423	-0.653339
C	0.221660	2.971004	-0.147916
C	-0.497611	1.798440	-0.370297
H	-1.692512	-0.333748	-1.073556
H	-3.754567	-2.245395	-1.391442
H	-1.520388	1.679025	-0.002628
H	2.063139	4.046427	-0.479398
H	1.865931	0.085982	-2.149661
H	0.884182	-1.748549	-1.654110
C	-0.409672	4.039548	0.709153
F	-0.584413	3.597583	1.963099
F	0.341275	5.139529	0.779096
F	-1.611337	4.395017	0.252012
C	3.481026	2.252218	-1.918838
F	4.316452	2.698003	-0.973511
F	3.981141	1.117507	-2.400248
F	3.502569	3.153524	-2.908108
C	3.437907	-2.926103	1.594172
C	2.273496	-3.002470	0.816420
C	2.239324	-3.890488	-0.267701
C	3.351405	-4.674928	-0.574054
C	4.506396	-4.587746	0.200642
C	4.543161	-3.713760	1.289205
C	1.055341	-2.247916	1.185978
C	-0.199343	-2.740150	1.214129
C	-1.164138	-1.735788	1.776164
C	-0.236015	-0.640047	2.330247
N	1.063170	-0.928556	1.703257
C	2.051891	0.005604	1.554562
O	3.048016	-0.112391	0.879018
O	1.756090	1.100631	2.272727
C	2.737754	2.120383	2.324196
H	-1.805242	-2.168303	2.561067
H	-0.138513	-0.700632	3.428260
H	-1.844334	-1.358700	0.991843
H	-0.558491	0.380063	2.078780
H	-0.465053	-3.758772	0.931426
H	3.530994	1.848342	3.041884
H	3.194609	2.288481	1.338755
H	2.221530	3.026767	2.671042
H	1.339354	-3.973613	-0.885768
H	3.308821	-5.357540	-1.427585
H	5.379329	-5.201228	-0.041027
H	5.443228	-3.644391	1.906977
H	3.477392	-2.252018	2.452960
C	-7.009166	-0.004526	-0.729314
H	-7.175471	-0.687725	-1.578414
H	-7.228347	-0.531173	0.215618
H	-7.673883	0.866655	-0.822919
C	-4.421342	-0.084310	2.175954
H	-3.387619	0.301902	2.178083
H	-5.125887	0.765947	2.148711
H	-4.594691	-0.666982	3.092672

aTS_exo

Zero-point correction= 0.491382
(Hartree/Particle)
Thermal correction to Energy= 0.531780
Thermal correction to Enthalpy= 0.532724
Thermal correction to Gibbs Free Energy= 0.409991
Sum of electronic and zero-point Energies= -2578.465005
Sum of electronic and thermal Energies= -2578.424607
Sum of electronic and thermal Enthalpies= -2578.423663
Sum of electronic and thermal Free Energies= -2578.546396
Quasi-Harmonic Approximation corrected Free energy= -2578.533928
E(wB97XD/Def2TZVPP)= -2581.72864867

C	-3.403609	2.333376	-0.506392
C	-3.496863	1.038040	0.019856
C	-3.820616	0.862864	1.373140
C	-4.058803	1.973560	2.177556
C	-3.982883	3.262003	1.644784
C	-3.656131	3.441246	0.301423
C	-3.064122	-0.099908	-0.818775
C	-1.819492	-0.148681	-1.401847
C	-1.687146	-1.386160	-2.248180
C	-2.932342	-2.203691	-1.856347
N	-3.741503	-1.271533	-1.039902
C	-4.985076	-1.620277	-0.531019
O	-5.722920	-0.892850	0.079729
O	-5.258577	-2.881670	-0.855473
C	-6.501137	-3.392890	-0.397564
O	2.545090	-2.133872	-0.461916
P	2.781968	-3.441142	0.218117
O	3.490851	-3.171274	1.633520
O	3.769820	-4.430017	-0.562457
O	1.545098	-4.368983	0.469328
O	-0.440041	-2.860778	0.289577
C	-1.188509	-1.980759	0.782611
O	-2.249141	-2.088980	1.390064
C	-0.707300	-0.545031	0.539483
N	0.554241	-0.341305	0.217930
C	1.167087	0.916416	0.098002
C	0.439792	2.105135	0.169614
C	1.100086	3.327467	0.025941
C	2.471153	3.379655	-0.184098
C	3.188362	2.180474	-0.255672
C	2.552448	0.955794	-0.121278
C	0.268874	4.585996	0.075982
H	1.132957	-1.165396	-0.061402
H	0.661243	-3.851296	0.445671
H	-0.757221	-1.941397	-2.042617
H	-2.678504	-3.084452	-1.250586
H	-1.689538	-1.123492	-3.320707
H	-3.521146	-2.535369	-2.723661
H	-1.218068	0.749517	-1.552759
H	-6.548445	-4.429813	-0.757583
H	-6.547646	-3.373639	0.703117
H	-7.340956	-2.809945	-0.809299
H	-1.243127	0.255617	1.053727
H	-0.642267	2.103255	0.325450
H	2.980641	4.338794	-0.296557
C	4.676984	2.247702	-0.495924
H	3.101967	0.013288	-0.186806
H	-3.843994	-0.144247	1.792895
H	-4.302624	1.830268	3.233950
H	-4.171234	4.130527	2.282430
H	-3.583283	4.447392	-0.119534
H	-3.136517	2.475307	-1.557515
F	1.012714	5.689026	-0.005006
F	-0.610781	4.622775	-0.935130
F	-0.440794	4.660721	1.206617
F	4.951063	2.806994	-1.680771
F	5.284377	2.994319	0.433483
F	5.249353	1.044391	-0.480392
C	3.734443	-4.226214	2.551018
H	2.786076	-4.644629	2.928459
H	4.327326	-5.033131	2.085206
H	4.303551	-3.796998	3.389166
C	5.031885	-3.955728	-1.008170
H	5.638121	-3.578251	-0.165523
H	5.548362	-4.809837	-1.470507
H	4.908309	-3.154059	-1.756012

aTS-I_anti

Zero-point correction= 0.490663
(Hartree/Particle)
Thermal correction to Energy= 0.530590
Thermal correction to Enthalpy= 0.531534
Thermal correction to Gibbs Free Energy= 0.413517
Sum of electronic and zero-point Energies= -2578.471115
Sum of electronic and thermal Energies= -2578.431188
Sum of electronic and thermal Enthalpies= -2578.430244
Sum of electronic and thermal Free Energies= -2578.548260
Quasi-Harmonic Approximation corrected Free energy= -2578.538016
E(wB97XD/Def2TZVPP)= -2581.72999342

O	-5.539227	-0.543708	-0.691885
P	-4.116845	-0.391930	0.029228
O	-4.440664	0.257504	1.462045
O	-3.589238	-1.804896	0.426159
O	-3.222823	0.436502	-0.837326
O	-2.550887	-2.902955	-1.490960
C	-1.431239	-2.862134	-2.067508
O	-1.023449	-3.547955	-2.992945
C	-0.401969	-1.810245	-1.613671
N	-0.764665	-0.555169	-1.437281
C	0.118700	0.534045	-1.326658
C	1.492719	0.403958	-1.520740
C	2.323779	1.502273	-1.306890
C	1.802827	2.731883	-0.922731
C	0.421173	2.853223	-0.756044
C	-0.422661	1.772099	-0.957493
C	3.814004	1.290387	-1.390662
C	0.090530	-2.816974	0.317536
C	0.941424	-1.940435	0.961612
N	2.260286	-2.354451	0.785537
C	2.301809	-3.495224	-0.153113
C	0.866368	-4.029239	-0.133162
C	0.508859	-0.697144	1.619011
C	1.347274	0.424053	1.759119
C	0.857424	1.616227	2.273036
C	-0.481141	1.723536	2.656619
C	-1.323848	0.622582	2.526280
C	-0.835547	-0.575958	2.011584
C	3.366389	-2.008241	1.550879
O	4.473419	-2.461737	0.963391
C	5.696295	-2.191839	1.633889
O	3.344703	-1.409608	2.594234
H	-1.770133	-0.318245	-1.262428
H	-3.095958	-2.302761	-0.396048
H	0.555067	-4.415904	-1.115745
H	2.597811	-3.136955	-1.154942
H	0.750340	-4.849686	0.598592
H	3.048251	-4.228996	0.174095
H	-0.980053	-2.830256	0.513122
H	6.482482	-2.643495	1.013487
H	5.860230	-1.105882	1.718937
H	5.701563	-2.642896	2.639297
H	0.607923	-1.997971	-1.976981

H	1.940698	-0.548627	-1.807033
H	2.462163	3.583103	-0.741278
C	-0.141248	4.178830	-0.306667
H	-1.500846	1.850235	-0.799763
H	-1.521352	-1.420684	1.925991
H	-2.373621	0.683487	2.820696
H	-0.863848	2.668499	3.052549
H	1.523333	2.478773	2.357072
H	2.383471	0.385491	1.431322
F	4.488523	2.435352	-1.470120
F	4.264267	0.641954	-0.299258
F	4.153522	0.542072	-2.443049
F	-0.056347	5.102461	-1.269074
F	-1.423371	4.089827	0.050854
F	0.533514	4.656095	0.748512
C	-4.812142	1.626387	1.538879
H	-5.700067	1.834033	0.915843
H	-5.057017	1.831531	2.591620
H	-3.982749	2.280645	1.219711
C	-6.536154	-1.416548	-0.181908
H	-6.232139	-2.469862	-0.302425
H	-6.739464	-1.213114	0.884256
H	-7.450129	-1.232222	-0.765851

aTS-II_syn

Zero-point correction= 0.492591
(Hartree/Particle)
Thermal correction to Energy= 0.531456
Thermal correction to Enthalpy= 0.532400
Thermal correction to Gibbs Free Energy= 0.415477
Sum of electronic and zero-point Energies= -
2578.486674
Sum of electronic and thermal Energies= -
2578.447809
Sum of electronic and thermal Enthalpies= -
2578.446865
Sum of electronic and thermal Free Energies= -
2578.563788
Quasi-Harmonic Approximation corrected Free energy= -2578.553190
E(wB97XD/Def2TZVPP)= -2581.746935

O	0.281535	4.203476	-0.973060
P	0.856991	2.900230	-0.198828
O	1.151444	1.866396	-1.278494
O	2.265088	3.403725	0.422967
O	-0.036822	2.507590	0.946889
O	-0.364182	0.084675	-1.864551
C	-1.161437	-0.521886	-1.072368
O	-2.241304	-0.992786	-1.412724
C	-0.798653	-0.757087	0.414571
N	0.439968	-0.253287	0.913022
C	1.672526	-0.753136	0.561957
C	1.856892	-1.971648	-0.102835
C	3.145867	-2.426119	-0.397815
C	4.271205	-1.686475	-0.066580
C	4.083003	-0.457843	0.579242
C	2.818026	0.002428	0.895890
H	0.409348	0.750436	1.133621
H	0.380235	0.844914	-1.515498
H	2.693501	0.968547	1.390260
H	5.272470	-2.045406	-0.308990
H	1.001548	-2.573457	-0.415781
H	-0.799753	-1.859093	0.490529
C	5.293508	0.384683	0.887304
F	5.012563	1.415824	1.688435
F	6.254613	-0.331558	1.484621
F	5.834075	0.892473	-0.230148
C	3.278449	-3.755727	-1.094611
F	4.548561	-4.077320	-1.349576
F	2.761545	-4.749055	-0.354012
F	2.622366	-3.771547	-2.261103
C	-5.284684	1.127936	0.065761
C	-3.894286	0.899688	0.070926
C	-3.036233	1.913989	-0.403311
C	-3.553832	3.084852	-0.937372
C	-4.934588	3.284258	-0.962542
C	-5.794914	2.314753	-0.441110
C	-3.293907	-0.276242	0.671726
C	-1.937435	-0.228969	1.340743
C	-2.151951	-1.166697	2.532730
C	-3.100434	-2.215461	1.966428
N	-3.887583	-1.432823	0.982191
C	-4.919442	-2.065572	0.223991
O	-5.481582	-1.582654	-0.710611
O	-5.113893	-3.275902	0.711504
C	-6.069905	-4.088783	0.030444
H	-2.629749	-0.627189	3.366377
H	-3.779463	-2.652399	2.709440
H	-1.205175	-1.593424	2.893970
H	-2.582025	-3.034647	1.442186
H	-1.685812	0.798310	1.636018
H	-7.063973	-3.615690	0.057988
H	-5.760980	-4.245951	-1.014714
H	-6.085141	-5.043157	0.572653
H	-1.952274	1.805274	-0.346582
H	-2.868625	3.843316	-1.323483
H	-5.345149	4.210159	-1.375491
H	-6.874304	2.485780	-0.431945
H	-5.971148	0.393552	0.484413
C	3.388531	3.595188	-0.416731
H	3.678739	2.654824	-0.914259
H	3.183726	4.359363	-1.188803
H	4.215441	3.941969	0.221297
C	-0.098314	5.339981	-0.225038
H	-0.844854	5.081200	0.546675
H	0.775232	5.805359	0.267427
H	-0.537092	6.064547	-0.929067

aTS-I_syn

Zero-point correction= 0.492463
(Hartree/Particle)
Thermal correction to Energy= 0.532360
Thermal correction to Enthalpy= 0.533304
Thermal correction to Gibbs Free Energy= 0.413915
Sum of electronic and zero-point Energies= -
2578.469981
Sum of electronic and thermal Energies= -
2578.430083
Sum of electronic and thermal Enthalpies= -
2578.429139
Sum of electronic and thermal Free Energies= -
2578.548528
Quasi-Harmonic Approximation corrected Free energy= -2578.537526
E(wB97XD/Def2TZVPP)= -2581.73103410

O	-5.030685	-1.483905	0.963255
P	-4.470652	-0.659556	-0.298691
O	-4.462751	-1.709996	-1.458429
O	-5.617657	0.384687	-0.674248
O	-3.194451	0.055895	0.002307
O	-2.242479	-2.810040	-1.196205
C	-1.042549	-2.941329	-1.538063
O	-0.478944	-3.846713	-2.139259
C	-0.123899	-1.777813	-1.101254
N	-0.657115	-0.576734	-0.924314
C	0.025523	0.652146	-0.886768
C	1.337530	0.797013	-1.352565
C	1.958966	2.037052	-1.274478
C	1.296911	3.145113	-0.746650
C	-0.008390	2.990595	-0.291325
C	-0.651450	1.757818	-0.361852
H	-1.671699	-0.529283	-0.697481

H	-3.592153	-2.253665	-1.471747
H	-1.681241	1.633201	-0.015294
H	1.796716	4.114011	-0.687323
H	1.889393	-0.046952	-1.766935
H	0.902050	-1.818425	-1.467525
C	-0.725774	4.142095	0.367311
F	-0.680858	4.030553	1.704944
F	-0.180084	5.320120	0.056316
F	-2.013762	4.189767	0.026172
C	3.380008	2.208778	-1.752339
F	4.149532	2.750761	-0.800191
F	3.938761	1.056715	-2.116162
F	3.438602	3.032958	-2.806659
C	3.832214	-2.528515	1.429354
C	2.675897	-2.691459	0.650687
C	2.720238	-3.543663	-0.466476
C	3.906929	-4.184325	-0.814487
C	5.054020	-4.004021	-0.043167
C	5.008871	-3.184260	1.085804
C	1.390118	-2.095905	1.051763
C	0.161158	-2.694868	0.852979
C	-0.880748	-2.024955	1.709383
C	-0.154643	-0.790613	2.270045
N	1.222860	-0.908625	1.740259
C	2.092093	0.178116	1.723294
O	3.138203	0.227415	1.130999
O	1.578801	1.167988	2.446722
C	2.355133	2.355782	2.542906
H	-1.191234	-2.711156	2.514277
H	-0.122696	-0.768997	3.370271
H	-1.789076	-1.760526	1.145465
H	-0.598421	0.155862	1.930328
H	0.082546	-3.742551	0.562616
H	3.219098	2.190604	3.207819
H	2.716982	2.668832	1.552640
H	1.689486	3.116994	2.970066
H	1.826130	-3.720815	-1.073627
H	3.928119	-4.833840	-1.693671
H	5.984685	-4.509481	-0.316616
H	5.900585	-3.051222	1.704486
H	3.811496	-1.899167	2.320184
C	-6.911537	-0.041203	-1.079150
H	-6.870905	-0.518357	-2.072365
H	-7.344962	-0.747805	-0.350104
H	-7.541178	0.859337	-1.130445
C	-5.083411	-0.872894	2.242007
H	-4.077979	-0.564698	2.576615
H	-5.748949	0.008777	2.235146
H	-5.486517	-1.622444	2.939299

rac-4l

Zero-point correction= 0.495983
(Hartree/Particle)
Thermal correction to Energy= 0.535606
Thermal correction to Enthalpy= 0.536550
Thermal correction to Gibbs Free Energy= 0.416187
Sum of electronic and zero-point Energies= -
2578.530114
Sum of electronic and thermal Energies= -
2578.490491
Sum of electronic and thermal Enthalpies= -
2578.489547
Sum of electronic and thermal Free Energies= -
2578.609910
Quasi-Harmonic Approximation corrected Free energy= -2578.597888
E(wB97XD/Def2TZVPP)= -2581.79120752

C	-4.482201	-2.064017	0.160304
C	-3.883730	-1.072561	-0.624692
C	-3.893579	-1.200960	-2.013338
C	-4.488864	-2.313840	-2.610032
C	-5.079951	-3.301448	-1.825477
C	-5.077437	-3.172130	-0.435213
C	-3.187900	0.081239	0.077702
C	-1.878013	-0.313636	0.795650
C	-1.783842	0.675712	1.961328
C	-3.239948	0.923321	2.349075
N	-3.962054	0.723144	1.090695
C	-5.248256	1.142942	0.856093
O	-5.820123	1.065713	-0.202089
O	-5.783628	1.649439	1.973455
C	-7.107613	2.145133	1.860666
O	1.190006	2.898772	0.555071
P	1.997100	3.261040	-0.641938
O	3.545694	2.957056	-0.386469
O	1.921534	4.802728	-1.063710
O	1.653495	2.506764	-1.986959
O	-0.891163	1.773336	-1.876709
C	-1.446372	1.017342	-1.116984
O	-2.751160	1.061022	-0.913321
C	-0.822905	-0.110362	-0.294562
N	0.507787	0.182058	0.110772
C	1.516112	-0.737584	0.244446
C	1.334849	-2.118074	0.062363
C	2.418428	-2.991415	0.169316
C	3.697948	-2.535484	0.465079
C	3.871027	-1.163862	0.668052
C	2.808742	-0.278324	0.572504
C	2.158374	-4.460589	-0.049129
H	0.711469	1.145136	0.400305
H	0.718458	2.157838	-2.019072
H	-1.313873	1.619209	1.633806
H	-3.407192	1.938422	2.736010
H	-1.182352	0.282886	2.793668
H	-3.593652	0.206695	3.111687
H	-1.925306	-1.352582	1.150332
H	-7.375426	2.517444	2.859547
H	-7.160066	2.966125	1.126961
H	-7.803041	1.345506	1.557824
H	-0.827499	-0.981183	-0.976190
H	0.345230	-2.527108	-0.152948
H	4.537414	-3.226066	0.544020
C	5.241126	-0.597706	0.945135
H	2.971375	0.789683	0.737797
H	-3.455919	-0.421387	-2.640520
H	-4.495588	-2.401173	-3.700225
H	-5.547896	-4.171086	-2.295645
H	-5.544766	-3.938822	0.189366
H	-4.488731	-1.964375	1.250599
F	3.244832	-5.208644	0.151140
F	1.204097	-4.917826	0.773916
F	1.733972	-4.703784	-1.296569
F	5.221169	0.292401	1.942270
F	6.125684	-1.543302	1.272467
F	5.731026	0.040490	-0.129727
C	2.114485	5.817352	-0.086428
H	1.992307	6.781689	-0.600829
H	1.366255	5.732483	0.719422
H	3.128923	5.761412	0.346104
C	4.519717	2.987839	-1.421560
H	5.502909	2.927491	-0.933837
H	4.390746	2.127384	-2.097903
H	4.454132	3.926400	-1.998877

rac-diast-4l

Zero-point correction= 0.496802
(Hartree/Particle)
Thermal correction to Energy= 0.536061
Thermal correction to Enthalpy= 0.537005
Thermal correction to Gibbs Free Energy= 0.418404
Sum of electronic and zero-point Energies= -
2578.528452

Sum of electronic and thermal Energies= -2578.489193
Sum of electronic and thermal Enthalpies= -2578.488249
Sum of electronic and thermal Free Energies= -2578.606849
Quasi-Harmonic Approximation corrected Free energy= -2578.595436
E(wB97XD/Def2TZVPP)= -2581.78904995

O	-0.435083	3.934184	1.715840
P	-0.923761	2.792849	0.706311
O	-1.510247	1.697918	1.685755
O	-2.201711	3.392922	-0.041384
O	0.103004	2.350013	-0.276636
O	0.294073	-0.243040	1.738898
C	1.069325	-0.393891	0.826253
O	2.370373	-0.205577	0.971983
C	0.750699	-0.830341	-0.603849
N	-0.460554	-0.285256	-1.139205
C	-1.721896	-0.731282	-0.791051
C	-1.977798	-1.992151	-0.237341
C	-3.286702	-2.384846	0.047609
C	-4.366781	-1.545878	-0.193598
C	-4.108893	-0.283697	-0.737103
C	-2.818428	0.120774	-1.036441
H	-0.395297	0.731283	-1.226329
H	-0.941650	0.888414	1.778077
H	-2.640358	1.113399	-1.455931
H	-5.386312	-1.859249	0.036898
H	-1.161096	-2.679989	-0.013239
H	0.692713	-1.932658	-0.580428
C	-5.269300	0.650835	-0.964154
F	-4.917023	1.755262	-1.628660
F	-6.248513	0.062934	-1.660056
F	-5.809916	1.048492	0.197577
C	-3.501333	-3.736054	0.682136
F	-4.770866	-4.140181	0.598058
F	-2.744539	-4.678959	0.104654
F	-3.175705	-3.725433	1.980840
C	5.263956	0.701388	-1.049077
C	4.034798	0.841611	-0.402968
C	3.631872	2.104951	0.041338
C	4.460949	3.208710	-0.145009
C	5.694143	3.063973	-0.782164
C	6.091831	1.807974	-1.237167
C	3.089498	-0.338916	-0.273743
C	1.999373	-0.394598	-1.376017
C	2.528347	-1.408382	-2.381689
C	3.251240	-2.434761	-1.507560
N	3.713728	-1.634441	-0.373642
C	4.625725	-2.060042	0.558692
O	5.024568	-1.403629	1.487286
O	5.014284	-3.314685	0.292181
C	5.959984	-3.876222	1.187407
H	3.242134	-0.917328	-3.062777
H	4.101977	-2.904876	-2.021546
H	1.725310	-1.851691	-2.990420
H	2.579428	-3.244848	-1.169620
H	1.830719	0.603335	-1.804250
H	6.894441	-3.291608	1.192597
H	5.555909	-3.918562	2.212023
H	6.155746	-4.893309	0.819357
H	2.657994	2.229959	0.520935
H	4.139652	4.191729	0.211488
H	6.344754	3.931734	-0.924839
H	7.056356	1.683865	-1.737869
H	5.584967	-0.281545	-1.404591
C	-3.399483	3.723935	0.652230
H	-3.880226	2.817963	1.053985
H	-3.194674	4.428192	1.477302
H	-4.067564	4.201434	-0.078295
C	0.159877	5.123704	1.213716
H	1.047577	4.892951	0.600790
H	-0.561782	5.701365	0.609719
H	0.462428	5.720517	2.086451

Re-cDC

Zero-point correction= 0.790768 (Hartree/Particle)
Thermal correction to Energy= 0.846269
Thermal correction to Enthalpy= 0.847213
Thermal correction to Gibbs Free Energy= 0.695259
Sum of electronic and zero-point Energies= -3672.954075
Sum of electronic and thermal Energies= -3672.898574
Sum of electronic and thermal Enthalpies= -3672.897630
Sum of electronic and thermal Free Energies= -3673.049584
Quasi-Harmonic Approximation corrected Free energy= -3673.034413
E(wB97XD/Def2TZVPP)= -3677.63635072

O	0.672435	0.672894	2.045065
P	0.779867	0.091115	0.682997
O	-0.529431	0.148087	-0.176363
O	1.854349	0.726787	-0.325814
C	3.187405	0.366810	-0.226447
C	4.081202	1.361793	0.248650
C	5.410764	1.033614	0.353458
C	5.878765	-0.273526	0.056905
C	4.961263	-1.264244	-0.399247
C	3.585637	-0.903387	-0.599800
C	7.246738	-0.618081	0.240025
C	7.686994	-1.897921	0.010250
C	6.769993	-2.894130	-0.402753
C	5.445286	-2.587502	-0.602035
C	3.568137	2.710890	0.626629
C	3.329450	3.672885	-0.375257
C	2.839996	4.972199	-0.005932
C	2.611323	5.261704	1.342335
C	2.838485	4.312529	2.342028
C	3.321163	3.006830	1.982103
C	3.554059	3.405926	-1.766834
C	3.306147	4.356471	-2.717097
C	2.820015	5.645689	-2.347140
C	2.596775	5.942081	-1.032729
C	2.595478	4.608612	3.723443
C	2.806805	3.668508	4.690797
C	3.271200	2.367035	4.335443
C	3.518077	2.046994	3.030806
C	2.585126	-1.864588	-1.145478
C	1.437165	-2.118245	-0.423318
C	0.415610	-3.012397	-0.843963
C	0.615054	-3.686645	-2.024979
C	1.753730	-3.446969	-2.839380
C	2.736913	-2.502428	-2.422620
C	1.904352	-4.105254	-4.091396
C	2.966722	-3.820636	-4.913325
C	3.920345	-2.851028	-4.520962
C	3.809319	-2.209037	-3.310625
O	1.248121	-1.450249	0.770070
C	-0.837568	-3.161649	-0.047330
C	-0.799146	-3.772834	1.223837
C	-2.006029	-3.881793	1.995542
C	-3.210105	-3.419587	1.458550
C	-3.266298	-2.831230	0.192605
C	-2.056225	-2.669834	-0.568265
C	-1.950701	-4.462239	3.304358
C	-0.774030	-4.928049	3.814684
C	0.421023	-4.850861	3.039838
C	0.409762	-4.297005	1.791222
C	-2.145385	-1.974124	-1.821633
C	-3.343987	-1.524441	-2.301688
C	-4.548694	-1.741275	-1.570889
C	-4.506083	-2.366729	-0.358075
H	4.548918	-1.456282	-3.030867
H	4.750779	-2.605876	-5.189116
H	3.071020	-4.328757	-5.876080

H	1.147878	-4.835937	-4.393000
H	-0.142236	-4.395178	-2.372552
H	6.122306	1.785301	0.706876
H	4.751735	-3.372840	-0.908714
H	7.117208	-3.919449	-0.558721
H	8.739647	-2.154344	0.159131
H	7.940355	0.156584	0.580355
H	-4.128172	-3.516748	2.046348
H	2.241445	6.253743	1.620995
H	3.928724	2.423773	-2.066176
H	3.480781	4.130155	-3.772940
H	2.628096	6.393223	-3.122211
H	2.224416	6.928396	-0.738979
H	3.857793	1.041080	2.774461
H	3.419700	1.616635	5.117084
H	2.615600	3.904901	5.741584
H	2.233190	5.607526	3.985502
H	1.335401	-4.251598	1.212652
H	1.356371	-5.237985	3.454161
H	-0.740569	-5.362029	4.817904
H	-2.873024	-4.513348	3.889336
H	-5.422451	-2.516719	0.221226
H	-5.500704	-1.384709	-1.973164
H	-3.379860	-0.981696	-3.250528
H	-1.232772	-1.784736	-2.389304
O	-0.896583	-0.646048	3.621906
C	-2.198235	-0.618775	3.563285
O	-2.909879	-1.290312	4.268272
C	-2.941350	0.270318	2.589554
N	-2.692375	0.377935	1.346584
C	-3.650716	0.975821	0.491246
C	-5.017491	0.749472	0.661137
C	-5.929449	1.283047	-0.246587
C	-5.493884	2.037910	-1.330355
C	-4.125939	2.254543	-1.498256
C	-3.204086	1.725983	-0.601254
H	-1.358507	0.102342	0.420536
H	-0.378719	-0.033301	3.014753
H	-2.134896	1.886276	-0.751221
H	-6.210884	2.445520	-2.045403
H	-5.371411	0.112443	1.475113
H	-3.851459	0.705054	3.034578
C	-3.635078	2.995478	-2.718176
F	-4.507149	3.925966	-3.116393
F	-2.468323	3.601729	-2.499124
F	-3.458823	2.158616	-3.749910
C	-7.389068	0.944313	-0.078784
F	-8.177523	1.719716	-0.822483
F	-7.631546	-0.326692	-0.434671
F	-7.779922	1.068553	1.192973

Re-cDC_Ph

Zero-point correction= 0.780286 (Hartree/Particle)
Thermal correction to Energy= 0.828625
Thermal correction to Enthalpy= 0.829569
Thermal correction to Gibbs Free Energy= 0.695150
Sum of electronic and zero-point Energies= -2999.563127
Sum of electronic and thermal Energies= -2999.514789
Sum of electronic and thermal Enthalpies= -2999.513845
Sum of electronic and thermal Free Energies= -2999.648264
Quasi-Harmonic Approximation corrected Free energy= -2999.636161
E(wB97XD/Def2TZVPP)= -3003.43353012

O	0.150746	-1.272349	1.584021
P	0.063939	-0.418060	0.371926
O	1.061526	-0.777504	-0.773590
O	-1.333633	-0.381106	-0.427146
C	-2.351589	0.481410	-0.080855
C	-3.538052	-0.112628	0.424868
C	-4.580211	0.714081	0.764459
C	-4.466351	2.126548	0.670712
C	-3.261060	2.705159	0.176519
C	-2.200343	1.842761	-0.266407
C	-5.527695	2.974023	1.093344
C	-5.395354	4.340101	1.057720
C	-4.184483	4.917481	0.606336
C	-3.147064	4.124156	0.178137
C	-3.618009	-1.594540	0.578443
C	-3.950210	-2.395517	-0.532264
C	-4.025574	-3.822070	-0.378273
C	-3.769730	-4.392303	0.872088
C	-3.430452	-3.607168	1.976814
C	-3.345191	-2.179433	1.830688
C	-4.212896	-1.840501	-1.828936
C	-4.524616	-2.643091	-2.890485
C	-4.601242	-4.059180	-2.733870
C	-4.360372	-4.627689	-1.515468
C	-3.157866	-4.193172	3.256262
C	-2.810704	-3.416573	4.324265
C	-2.709427	-2.000930	4.178202
C	-2.966056	-1.403821	2.976975
C	-0.940660	2.382921	-0.853240
C	0.266160	2.040185	-0.280438
C	1.521642	2.553749	-0.706279
C	1.523773	3.426605	-1.766155
C	0.325986	3.764679	-2.453589
C	-0.919887	3.228070	-2.014138
C	0.355263	4.615509	-3.592839
C	-0.793873	4.903926	-4.287451
C	-2.025264	4.341093	-3.875716
C	-2.087452	3.526010	-2.770485
O	0.274754	1.136931	0.761560
C	2.763145	2.142452	0.013412
C	3.013514	2.663893	1.300803
C	4.173507	2.232319	2.028548
C	5.042121	1.304512	1.449414
C	4.809996	0.788485	0.173326
C	3.651745	1.212378	-0.566376
C	4.410319	2.754130	3.341504
C	3.554109	3.657336	3.903234
C	2.405760	4.096253	3.180338
C	2.144529	3.618244	1.926862
C	3.440494	0.638143	-1.866040
C	4.311804	-0.276194	-2.386884
C	5.463523	-0.687920	-1.651258
C	5.699463	-0.175026	-0.408198
H	-3.044535	3.091572	-2.474648
H	-2.935240	4.551960	-4.444704
H	-0.759081	5.555988	-5.164765
H	1.316291	5.029417	-3.912587
H	2.470449	3.852802	-2.110427
H	-5.508162	0.279334	1.146941
H	-2.217253	4.588800	-0.155562
H	-4.069959	6.005168	0.603798
H	-6.215356	4.982887	1.390064
H	-6.450144	2.513683	1.459914
H	5.912742	0.961604	2.015093
H	-3.831301	-5.479263	0.987014
H	-4.159337	-0.757159	-1.963589
H	-4.717867	-2.198906	-3.871274
H	-4.854458	-4.684690	-3.594817
H	-4.417258	-5.712878	-1.385405
H	-2.869970	-0.320123	2.878170
H	-2.412742	-1.391404	5.036413
H	-2.601483	-3.875040	5.295136
H	-3.231034	-5.280487	3.356850
H	1.260870	3.969017	1.388173
H	1.727271	4.822347	3.637612
H	3.742046	4.046738	4.907905
H	5.292781	2.406150	3.886610
H	6.572736	-0.488172	0.171546
H	6.151369	-1.416688	-2.089738
H	4.122200	-0.706899	-3.374241
H	2.555089	0.927141	-2.434753

O	2.580815	-1.156762	2.577202
C	3.624736	-1.828047	2.161276
O	4.711970	-1.735916	2.673537
C	3.505350	-2.779692	0.993664
N	2.820425	-2.559163	-0.053167
C	2.901962	-3.445775	-1.157848
C	4.121902	-3.983061	-1.584634
C	4.153558	-4.836925	-2.685347
C	2.975631	-5.158151	-3.360326
C	1.762183	-4.612995	-2.937632
C	1.722203	-3.748136	-1.847387
H	1.824111	-1.399932	-0.450612
H	1.690871	-1.354221	2.159833
H	0.775764	-3.311865	-1.519838
H	3.004493	-5.827593	-4.224631
H	5.048613	-3.707525	-1.074435
H	4.154571	-3.665610	1.084052
H	5.109700	-5.247614	-3.021986
H	0.835556	-4.856311	-3.464855

Re-cDC_TRIP

Zero-point correction= 1.110908
(Hartree/Particle)
Thermal correction to Energy= 1.181472
Thermal correction to Enthalpy= 1.182416
Thermal correction to Gibbs Free Energy= 1.001555
Sum of electronic and zero-point Energies= -
3765.640842
Sum of electronic and thermal Energies= -
3765.570278
Sum of electronic and thermal Enthalpies= -
3765.569334
Sum of electronic and thermal Free Energies= -
3765.750195
Quasi-Harmonic Approximation corrected Free energy= -3765.733242
E(wB97XD/Def2TZVPP)= -3770.81472046

O	0.868483	0.651942	2.043052
P	0.766547	-0.102529	0.768781
O	-0.581867	0.107571	-0.002708
O	1.828098	0.185876	-0.393838
C	3.128603	-0.262596	-0.245706
C	4.109759	0.740567	-0.026148
C	5.414311	0.327263	0.087440
C	5.760770	-1.051219	0.086791
C	4.748573	-2.038048	-0.097407
C	3.399711	-1.613509	-0.351476
C	7.103930	-1.464408	0.306648
C	7.429658	-2.796653	0.376513
C	6.418231	-3.777173	0.239528
C	5.114472	-3.408775	0.007658
C	3.676344	2.166565	0.101931
C	3.186067	2.862296	-1.024541
C	2.747988	4.177302	-0.864105
C	2.765575	4.823940	0.373168
C	3.250315	4.114739	1.470912
C	3.699376	2.793630	1.365765
C	2.307700	-2.558646	-0.728047
C	1.104862	-2.524572	-0.050188
C	-0.029096	-3.313422	-0.390513
C	0.145593	-4.261045	-1.369459
C	1.350459	-4.355645	-2.115505
C	2.422627	-3.455579	-1.846386
C	1.468484	-5.284872	-3.185804
C	2.583386	-5.299384	-3.987097
C	3.623392	-4.367261	-3.759493
C	3.545639	-3.470388	-2.720461
O	0.976768	-1.673703	1.030647
C	-1.370355	-3.024559	0.210565
C	-1.634476	-3.297311	1.568699
C	-2.896837	-2.998570	2.090238
C	-3.908989	-2.443380	1.305308
C	-3.617852	-2.163394	-0.029727
C	-2.370022	-2.434568	-0.599096
H	4.353557	-2.752011	-2.572309
H	4.495327	-4.355072	-4.419641
H	2.661678	-6.013742	-4.811521
H	0.642142	-5.978133	-3.369865
H	-0.686556	-4.918715	-1.634114
H	6.205148	1.069192	0.227759
H	4.344416	-4.177456	-0.084931
H	6.674582	-4.836966	0.324814
H	8.464671	-3.102547	0.553392
H	7.873030	-0.696708	0.434606
O	-0.902144	0.480666	3.948199
C	-2.177867	0.770403	3.958838
O	-2.884436	0.578047	4.915509
C	-2.836169	1.377267	2.737454
N	-2.430084	1.221435	1.546336
C	-3.190677	1.695409	0.452589
C	-4.505944	1.277826	0.258926
C	-5.172422	1.638016	-0.910441
C	-4.544707	2.419057	-1.877520
C	-3.231480	2.838714	-1.665502
C	-2.546844	2.473210	-0.511816
H	-1.289245	0.413300	0.651877
H	-0.360614	0.660941	3.126093
H	-1.506123	2.768863	-0.360014
H	-5.068853	2.686558	-2.797017
H	-4.986566	0.637693	1.002722
H	-3.764767	1.925015	2.968828
C	-2.492517	3.595281	-2.742690
F	-3.322757	4.271031	-3.538985
F	-1.623805	4.471192	-2.228538
F	-1.791582	2.759384	-3.518452
C	-6.548199	1.074937	-1.164507
F	-7.252982	1.826860	-2.008823
F	-6.469771	-0.153947	-1.700887
F	-7.253816	0.957616	-0.036206
C	-0.604695	-3.960370	2.473845
H	0.343538	-4.033125	1.920148
C	-1.028185	-5.394879	2.814977
C	-0.326031	-3.140589	3.737519
H	-0.023576	-2.110803	3.488835
H	-1.210406	-3.082818	4.396758
H	0.491050	-3.602151	4.320118
H	-1.968890	-5.410994	3.394548
H	-0.251250	-5.895508	3.420268
H	-1.185171	-5.992478	1.899706
C	-2.162326	-2.104367	-2.075097
H	-1.084008	-2.149244	-2.288083
C	-2.623525	-0.691371	-2.451461
C	-2.859526	-3.143234	-2.964245
H	-2.526450	-4.170100	-2.737729
H	-3.955145	-3.111033	-2.821558
H	-2.651082	-2.945171	-4.030995
H	-3.717194	-0.570192	-2.359533
H	-2.359119	-0.476647	-3.502040
H	-2.129795	0.064803	-1.822808
C	-5.308794	-2.210861	1.850533
H	-5.854445	-1.612914	1.097957
C	-5.321874	-1.428667	3.165694
C	-6.055842	-3.543075	1.996753
H	-6.085797	-4.090135	1.038610
H	-5.561940	-4.192926	2.741705
H	-7.096146	-3.375393	2.328874
H	-4.840106	-1.988657	3.985737
H	-6.358998	-1.211864	3.477145
H	-4.790018	-0.466742	3.075901
H	-4.403678	-1.726236	-0.653653
H	-3.099873	-3.230715	3.140238
C	4.200191	2.086706	2.620890
H	4.258093	1.009567	2.402324
C	3.249433	2.238883	3.813502
C	5.612702	2.562276	2.984884
H	6.321297	2.413631	2.152018
H	5.614565	3.639146	3.234525
H	5.996680	2.010405	3.861732
H	3.210219	3.277858	4.186852

H	3.594033	1.605829	4.650603
H	2.230109	1.920166	3.543557
C	3.124614	2.237498	-2.413431
H	3.436070	1.184584	-2.335115
C	4.113842	2.918206	-3.367548
C	1.699962	2.243676	-2.982502
H	0.997328	1.744165	-2.295562
H	1.332471	3.269230	-3.163650
H	1.668585	1.706422	-3.947257
H	3.858447	3.981829	-3.523967
H	4.106023	2.421726	-4.354581
H	5.143571	2.873401	-2.971428
C	2.191185	6.226634	0.494420
H	2.368047	6.729631	-0.473252
C	2.848571	7.077770	1.582391
C	0.670871	6.153247	0.700984
H	0.179863	5.603675	-0.120802
H	0.431577	5.633401	1.646742
H	0.229276	7.165343	0.745279
H	2.604082	6.711641	2.595392
H	2.490354	8.120358	1.519070
H	3.947708	7.089434	1.479621
H	3.271933	4.596838	2.451356
H	2.370230	4.724436	-1.734894

Si-cDC

Zero-point correction= 0.791297
(Hartree/Particle)
Thermal correction to Energy= 0.846404
Thermal correction to Enthalpy= 0.847348
Thermal correction to Gibbs Free Energy= 0.700070
Sum of electronic and zero-point Energies= -
3672.965127
Sum of electronic and thermal Energies= -
3672.910020
Sum of electronic and thermal Enthalpies= -
3672.909076
Sum of electronic and thermal Free Energies= -
3673.056354
Quasi-Harmonic Approximation corrected Free energy= -3673.043556
E(wB97XD/Def2TZVPP)= -3677.64311666

C	-2.464661	-2.064786	2.342721
N	-1.867857	-2.069891	1.218182
C	-2.576464	-1.777606	0.023272
C	-3.947841	-1.997777	-0.123760
C	-4.578705	-1.672578	-1.323439
C	-3.860668	-1.138719	-2.388542
C	-2.488736	-0.943408	-2.244733
C	-1.848198	-1.265331	-1.055023
C	-6.066493	-1.891662	-1.432035
C	-1.859116	-2.574025	3.630935
O	-2.534461	-3.364742	4.240458
O	-0.672852	-2.190254	4.018414
O	0.637333	-0.316132	2.804470
P	1.006821	-0.468416	1.373573
O	0.718129	-1.872546	0.744979
O	0.234486	0.557161	0.376177
C	0.414819	1.898800	0.654002
C	-0.693723	2.620048	1.167577
C	-0.478890	3.920994	1.554781
C	0.806770	4.521054	1.470322
C	1.885025	3.797441	0.883226
C	1.647333	2.468031	0.401252
C	1.033891	5.841195	1.948034
C	2.279772	6.413463	1.868092
C	3.357342	5.685438	1.308489
C	3.165822	4.412896	0.826033
C	-2.065720	2.025250	1.165793
C	-2.613449	1.448958	2.328432
C	-3.971454	0.974736	2.312952
C	-4.724926	1.080272	1.141457
C	-4.186509	1.634306	-0.022868
C	-2.828977	2.105648	-0.019853
C	-1.860321	1.302116	3.540419
C	-2.411330	0.717788	4.646749
C	-3.763674	0.261292	4.633692
C	-4.520014	0.393365	3.503647
C	-4.964614	1.748774	-1.220572
C	-4.431926	2.291220	-2.354979
C	-3.080323	2.743140	-2.362411
C	-2.307401	2.655132	-1.238390
C	2.637458	1.706410	-0.413043
C	3.012790	0.426236	-0.058466
C	3.883703	-0.380076	-0.836869
C	4.419009	0.165908	-1.976803
C	4.058744	1.466244	-2.417144
C	3.126632	2.233465	-1.659365
C	4.570843	1.987715	-3.637339
C	4.154705	3.206664	-4.111931
C	3.191629	3.949025	-3.388337
C	2.690848	3.477324	-2.198392
O	2.547079	-0.127806	1.119999
C	4.125608	-1.800981	-0.455961
C	5.120338	-2.130438	0.483852
C	5.325152	-3.506542	0.842051
C	4.528463	-4.494400	0.255731
C	3.533732	-4.175675	-0.672534
C	3.325642	-2.802665	-1.042251
C	6.340999	-3.830938	1.799587
C	7.104749	-2.853998	2.372165
C	6.900317	-1.486262	2.020729
C	5.943983	-1.136654	1.109141
C	2.284412	-2.507471	-1.986069
C	1.514557	-3.500803	-2.521782
C	1.727690	-4.863583	-2.156092
C	2.706390	-5.188035	-1.260907
H	-0.247084	-2.158300	0.962682
H	1.938796	4.063292	-1.667854
H	2.836150	4.905452	-3.782080
H	4.550095	3.597233	-5.053747
H	5.294722	1.389121	-4.198429
H	5.099623	-0.432685	-2.588951
H	-1.314998	4.510400	1.942152
H	4.007478	3.858156	0.404953
H	4.352095	6.137735	1.264567
H	2.445164	7.427013	2.243989
H	0.196803	6.390254	2.389407
H	4.683348	-5.541423	0.535311
H	-5.758743	0.722987	1.134901
H	-0.822092	1.636658	3.561460
H	-1.810910	0.594175	5.552090
H	-4.186478	-0.198403	5.531242
H	-5.557543	0.045545	3.482911
H	-1.273042	3.004911	-1.267827
H	-2.656671	3.157942	-3.280918
H	-5.033557	2.371700	-3.264743
H	-5.997482	1.388769	-1.205512
H	5.797838	-0.085389	0.847508
H	7.516385	-0.713027	2.488811
H	7.875275	-3.113637	3.103772
H	6.489661	-4.882148	2.065011
H	2.875148	-6.230034	-0.972106
H	1.100763	-5.644698	-2.595805
H	0.721519	-3.250057	-3.231516
H	2.099555	-1.468442	-2.269893
H	-0.240709	-1.439537	3.506226
H	-0.775779	-1.105096	-0.961084
C	-1.664786	-0.438841	-3.403084
H	-4.363005	-0.881289	-3.321702
H	-4.530523	-2.442143	0.686468
H	-3.531693	-1.815584	2.443188
F	-6.551415	-1.502840	-2.611496
F	-6.389868	-3.177698	-1.266336
F	-6.729412	-1.205683	-0.487084
F	-0.632492	0.304652	-2.999403
F	-1.153595	-1.458404	-4.112098
F	-2.388396	0.294197	-4.252949

Si-cDC_Ph

Zero-point correction= 0.780598 (Hartree/Particle)
Thermal correction to Energy= 0.828762
Thermal correction to Enthalpy= 0.829706
Thermal correction to Gibbs Free Energy= 0.696313
Sum of electronic and zero-point Energies= -2999.566524
Sum of electronic and thermal Energies= -2999.518360
Sum of electronic and thermal Enthalpies= -2999.517416
Sum of electronic and thermal Free Energies= -2999.650808
Quasi-Harmonic Approximation corrected Free energy= -2999.639206
E(wB97XD/Def2TZVPP)= -3003.43524751

C	3.017851	-3.008202	-1.095220
N	2.289712	-2.695808	-0.099066
C	2.896236	-2.498369	1.172114
C	3.955398	-3.301887	1.612036
C	4.523469	-3.077981	2.864728
C	4.036525	-2.061746	3.686233
C	2.969993	-1.274014	3.252999
C	2.393747	-1.492511	2.005656
C	2.508368	-3.482890	-2.437234
O	3.094639	-4.423423	-2.913531
O	1.476681	-2.920886	-3.007349
O	0.376742	-0.756802	-2.144956
P	-0.248532	-0.650650	-0.801875
O	-0.222330	-1.947399	0.064803
O	0.433137	0.468283	0.161504
C	0.469176	1.743760	-0.367281
C	1.725768	2.238159	-0.806078
C	1.743116	3.466427	-1.420452
C	0.547903	4.203985	-1.642495
C	-0.693657	3.705454	-1.150155
C	-0.707110	2.462365	-0.433304
C	0.570900	5.438115	-2.348766
C	-0.587418	6.138953	-2.579807
C	-1.824519	5.630010	-2.116992
C	-1.877103	4.446983	-1.419245
C	2.975813	1.471551	-0.515548
C	3.567443	0.649766	-1.495419
C	4.774527	-0.067667	-1.183045
C	5.346838	0.064586	0.084031
C	4.773286	0.882421	1.060462
C	3.559292	1.591514	0.764322
C	3.001764	0.480452	-2.802824
C	3.579553	-0.351458	-3.719745
C	4.777854	-1.061119	-3.407839
C	5.359350	-0.915653	-2.180403
C	5.369972	1.026496	2.354617
C	4.798174	1.818168	3.308854
C	3.583638	2.510081	3.026251
C	2.986587	2.402408	1.800828
C	-1.916041	1.938105	0.266675
C	-2.379822	0.659272	0.024007
C	-3.514278	0.100778	0.670623
C	-4.189748	0.879668	1.576427
C	-3.739034	2.182512	1.915307
C	-2.574086	2.711161	1.286469
C	-4.411493	2.949260	2.906704
C	-3.936632	4.179429	3.288446
C	-2.754329	4.687860	2.700211
C	-2.091575	3.975806	1.728985
O	-1.762378	-0.140406	-0.914632
C	-3.918417	-1.305791	0.382131
C	-4.749763	-1.589031	-0.718741
C	-5.126398	-2.949122	-0.988301
C	-4.657894	-3.969312	-0.155479
C	-3.827086	-3.698475	0.934876
C	-3.446661	-2.340760	1.213935
C	-5.971861	-3.226512	-2.111897
C	-6.414974	-2.221295	-2.923405
C	-6.039398	-0.870262	-2.660079
C	-5.237360	-0.564536	-1.596572
C	-2.579063	-2.098031	2.331163
C	-2.130536	-3.124689	3.112509
C	-2.515540	-4.470627	2.837012
C	-3.336926	-4.746231	1.781580
H	0.710581	-2.398764	-0.018939
H	-1.175121	4.384308	1.299792
H	-2.359535	5.654799	3.024883
H	-4.458123	4.759464	4.054972
H	-5.310903	2.531697	3.369123
H	-5.073272	0.475122	2.078464
H	2.695176	3.884643	-1.759752
H	-2.839614	4.061613	-1.075460
H	-2.747451	6.180088	-2.321342
H	-0.559279	7.083791	-3.129759
H	1.531015	5.814667	-2.714072
H	-4.943549	-5.004868	-0.366082
H	6.265619	-0.482663	0.317254
H	2.078069	1.005024	-3.050357
H	3.116458	-0.484369	-4.701112
H	5.222329	-1.724269	-4.155206
H	6.278941	-1.454473	-1.931545
H	2.060417	2.947185	1.602231
H	3.128912	3.137018	3.798856
H	5.262633	1.920848	4.293792
H	6.295251	0.480931	2.562800
H	-4.957628	0.474917	-1.406620
H	-6.397533	-0.074343	-3.319471
H	-7.057032	-2.445998	-3.779956
H	-6.252405	-4.265931	-2.308070
H	-3.633649	-5.775971	1.559784
H	-2.144772	-5.279293	3.473490
H	-1.461886	-2.917524	3.953026
H	-2.265902	-1.073973	2.548106
H	1.128826	-2.068188	-2.602118
H	1.569318	-0.862973	1.669267
H	4.484226	-1.887956	4.668776
H	4.314742	-4.126542	0.990510
H	4.111200	-3.090275	-1.004866
H	5.344960	-3.715207	3.204657
H	2.584136	-0.471088	3.886623

Si-cDC_TRIP

Zero-point correction= 1.111043 (Hartree/Particle)
Thermal correction to Energy= 1.181262
Thermal correction to Enthalpy= 1.182207
Thermal correction to Gibbs Free Energy= 1.001086
Sum of electronic and zero-point Energies= -3765.643080
Sum of electronic and thermal Energies= -3765.572861
Sum of electronic and thermal Enthalpies= -3765.571917
Sum of electronic and thermal Free Energies= -3765.753037
Quasi-Harmonic Approximation corrected Free energy= -3765.735544
E(wB97XD/Def2TZVPP)= -3770.81598266

C	2.411009	-1.905995	-2.086870
N	1.531132	-2.120689	-1.191904
C	1.905613	-2.402073	0.148163
C	3.159527	-2.908501	0.497603
C	3.474679	-3.130291	1.835775
C	2.543226	-2.884841	2.840848
C	1.283259	-2.409787	2.484780

C	0.961734	-2.170856	1.154058
C	4.884386	-3.540602	2.179783
C	2.117999	-1.864486	-3.570091
O	2.903521	-2.461442	-4.262414
O	1.040013	-1.294635	-4.046432
O	-0.296958	0.584587	-2.811522
P	-0.740386	0.296476	-1.423961
O	-0.919595	-1.225910	-1.090284
O	0.279209	0.819840	-0.277315
C	0.350128	2.180123	-0.022960
C	1.542206	2.867341	-0.384329
C	1.596538	4.214413	-0.117859
C	0.490161	4.919105	0.425767
C	-0.691128	4.207396	0.780582
C	-0.717550	2.783092	0.612528
C	0.538483	6.328146	0.613921
C	-0.547186	7.009513	1.106323
C	-1.734971	6.308211	1.425471
C	-1.805576	4.944675	1.269459
C	2.695502	2.098826	-0.950059
C	2.843537	1.949379	-2.348343
C	3.855928	1.112847	-2.820398
C	4.745793	0.453749	-1.962767
C	4.614616	0.670147	-0.592221
C	3.609504	1.493421	-0.066894
C	-1.841809	1.948378	1.120165
C	-2.512290	1.086627	0.276936
C	-3.613527	0.282352	0.674861
C	-4.045130	0.411504	1.972928
C	-3.358182	1.218279	2.917297
C	-2.219423	1.967650	2.506031
C	-3.760566	1.249527	4.280965
C	-3.044269	1.963384	5.209596
C	-1.885009	2.671260	4.811818
C	-1.483809	2.675650	3.497254
O	-2.120024	1.008605	-1.052407
C	-4.255530	-0.674168	-0.278525
C	-5.076906	-0.192887	-1.315082
C	-5.686409	-1.112085	-2.175237
C	-5.501409	-2.486358	-2.040986
C	-4.671861	-2.938032	-1.011656
C	-4.036516	-2.063479	-0.128916
H	-0.048544	-1.744835	-1.263250
H	-0.579952	3.217686	3.212720
H	-1.299027	3.214878	5.558314
H	-3.356193	1.973862	6.257731
H	-4.644158	0.677358	4.579315
H	-4.919895	-0.154247	2.303353
H	2.514616	4.767474	-0.333552
H	-2.732202	4.419676	1.511228
H	-2.605952	6.857033	1.794654
H	-0.502574	8.093852	1.241461
H	1.455576	6.861749	0.346811
H	0.563127	-0.616796	-3.479250
H	-0.021059	-1.773833	0.898516
C	0.246157	-2.136408	3.546476
H	2.800519	-3.050426	3.888396
H	3.899742	-3.146542	-0.268243
H	3.487841	-1.896601	-1.868422
F	4.981821	-4.037487	3.412313
F	5.355336	-4.458342	1.331905
F	5.712739	-2.486054	2.109616
F	-0.552866	-1.122974	3.203946
F	-0.538691	-3.203824	3.751831
F	0.805738	-1.830151	4.718614
C	3.526130	1.715735	1.440556
H	2.964632	2.648096	1.607448
C	4.899409	1.901431	2.094487
C	2.752485	0.587162	2.127117
H	1.736555	0.479745	1.715169
H	3.281519	-0.371478	1.989392
H	2.661318	0.772464	3.212798
H	5.479847	0.962103	2.117811
H	4.775392	2.230436	3.141333
H	5.501362	2.662621	1.568070
C	1.990376	2.755230	-3.321654
H	0.987165	2.858487	-2.879262
C	1.815659	2.109281	-4.697259
C	2.584003	4.161825	-3.496654
H	2.660943	4.705687	-2.542144
H	3.597862	4.102806	-3.932691
H	1.954478	4.763020	-4.176859
H	2.745175	2.150657	-5.292936
H	1.041465	2.652688	-5.265967
H	1.498049	1.059539	-4.626507
C	5.837167	-0.421859	-2.564110
H	5.360511	-1.010327	-3.369938
C	6.464848	-1.409656	-1.580480
C	6.923720	0.447967	-3.212777
H	6.500840	1.122357	-3.976852
H	7.427438	1.072103	-2.452598
H	7.688182	-0.181361	-3.702285
H	7.054710	-0.896697	-0.800511
H	7.148238	-2.092967	-2.113280
H	5.706714	-2.029096	-1.070619
H	3.971650	0.968223	-3.898784
H	5.310578	0.187573	0.097484
C	-5.340372	1.293544	-1.518649
H	-4.742211	1.853798	-0.783721
C	-6.809273	1.637711	-1.243636
C	-4.900277	1.767367	-2.908470
H	-3.841862	1.519639	-3.094544
H	-5.506137	1.305899	-3.708792
H	-5.014596	2.862831	-2.995016
H	-7.484496	1.133093	-1.958038
H	-6.977078	2.726025	-1.334280
H	-7.105658	1.330798	-0.225105
C	-3.142769	-2.633558	0.968550
H	-2.495063	-1.818374	1.327728
C	-2.224010	-3.758954	0.477554
C	-3.970107	-3.126161	2.163765
H	-4.610637	-2.331448	2.579344
H	-4.628381	-3.962506	1.865547
H	-3.307139	-3.482934	2.971575
H	-2.783871	-4.686997	0.264916
H	-1.482568	-4.004948	1.257909
H	-1.684377	-3.470439	-0.438303
C	-6.166735	-3.463095	-2.996010
H	-6.778616	-2.866964	-3.695708
C	-7.109878	-4.423910	-2.263055
C	-5.129915	-4.227547	-3.827771
H	-4.467693	-3.534274	-4.374712
H	-4.493841	-4.866249	-3.188570
H	-5.626739	-4.881539	-4.566863
H	-6.557021	-5.076137	-1.563048
H	-7.636926	-5.076952	-2.981661
H	-7.869606	-3.872721	-1.681826
H	-4.507986	-4.013661	-0.898321
H	-6.330505	-0.744046	-2.981045

4l

Zero-point correction= 1.030563 (Hartree/Particle)
Thermal correction to Energy= 1.099089
Thermal correction to Enthalpy= 1.100033
Thermal correction to Gibbs Free Energy= 0.920983
Sum of electronic and zero-point Energies= -4342.404400
Sum of electronic and thermal Energies= -4342.335874
Sum of electronic and thermal Enthalpies= -4342.334930
Sum of electronic and thermal Free Energies= -4342.513981
Quasi-Harmonic Approximation corrected Free energy= -4342.495673
E(wB97XD/Def2TZVPP)= -4348.06122045

C	-6.889078	-0.047620	0.419642
C	-5.972279	0.421878	-0.526721

C	-6.022122	-0.072217	-1.829374
C	-6.963265	-1.041399	-2.177423
C	-7.864390	-1.520176	-1.227924
C	-7.828736	-1.015482	0.072996
C	-4.941789	1.454362	-0.097973
C	-4.021864	1.005862	1.057599
C	-3.666571	2.307874	1.778939
C	-4.920207	3.164756	1.620684
N	-5.492923	2.693271	0.357836
C	-6.439565	3.373356	-0.365872
O	-6.848678	3.050154	-1.453202
O	-6.851785	4.455299	0.306707
C	-7.815094	5.265201	-0.346349
O	0.730937	1.750820	0.159959
P	1.306814	1.038719	-1.001265
O	1.228128	-0.562538	-0.747879
C	1.889730	-1.424669	-1.594622
C	3.270647	-1.477850	-1.565952
C	3.940418	-2.305160	-2.527842
C	3.162374	-3.131099	-3.389507
C	1.746084	-3.093770	-3.303641
C	1.096884	-2.241075	-2.443259
C	5.355461	-2.313614	-2.681621
C	5.959435	-3.121667	-3.614779
C	5.185749	-3.967413	-4.445942
C	3.817221	-3.966322	-4.336726
C	4.012070	-0.673913	-0.555265
C	3.786701	0.684736	-0.462567
C	4.444864	1.526837	0.472174
C	5.369701	0.951781	1.309771
C	5.611672	-0.446583	1.304644
C	4.908229	-1.280026	0.388304
C	5.083293	-2.688603	0.488416
C	5.938514	-3.230255	1.417617
C	6.668142	-2.396757	2.298720
C	6.502524	-1.035205	2.243990
O	2.867040	1.270602	-1.314981
C	4.080837	2.969204	0.572901
C	3.252443	3.393159	1.632337
C	2.882865	4.778796	1.727656
C	3.350045	5.682748	0.770427
C	4.169395	5.271188	-0.284714
C	4.543887	3.888268	-0.389382
C	2.032686	5.200738	2.802442
C	1.569529	4.306940	3.724821
C	1.928186	2.929140	3.629538
C	2.737494	2.489210	2.621333
C	5.385817	3.496970	-1.482620
C	5.817112	4.409944	-2.403661
C	5.441883	5.782387	-2.298252
C	4.644864	6.197378	-1.269667
C	-0.394512	-2.188443	-2.396682
C	-1.086324	-2.881801	-1.381355
C	-2.522727	-2.911287	-1.399654
C	-3.213648	-2.231597	-2.406809
C	-2.538617	-1.519685	-3.400191
C	-1.101150	-1.496630	-3.401413
C	-3.220142	-3.648670	-0.388123
C	-2.537560	-4.321164	0.584396
C	-1.112134	-4.280348	0.617917
C	-0.411765	-3.586257	-0.328521
C	-0.441108	-0.751876	-4.435805
C	-1.155385	-0.078090	-5.386004
C	-2.581535	-0.109644	-5.385454
C	-3.248944	-0.813045	-4.424792
O	0.693255	1.338941	-2.421689
O	-1.905481	1.294405	-1.736271
C	-2.829308	1.141184	-0.980745
O	-4.015100	1.693374	-1.196041
C	-2.854517	0.342901	0.319800
N	-1.585510	0.328657	0.962928
C	-1.217900	-0.573703	1.924833
C	-2.118719	-1.492212	2.493059
C	-1.683213	-2.393218	3.460271
C	-0.358807	-2.423148	3.890575
C	0.530926	-1.507805	3.328163
C	0.118678	-0.593486	2.369479
C	-2.648615	-3.374462	4.071212
H	2.997751	1.430903	2.559481
H	1.546390	2.217264	4.366296
H	0.920368	4.640845	4.539631
H	1.760764	6.259052	2.864835
H	3.064097	6.736892	0.845303
H	4.352405	7.248165	-1.179727
H	5.797053	6.498178	-3.045252
H	6.456591	4.089088	-3.231110
H	5.685198	2.449776	-1.574398
H	4.518330	-3.349521	-0.171578
H	6.049588	-4.316361	1.482401
H	7.348214	-2.840858	3.031033
H	7.040978	-0.380605	2.935788
H	5.895542	1.577571	2.036536
H	3.206339	-4.601730	-4.984990
H	1.158115	-3.741560	-3.960323
H	5.679973	-4.609367	-5.180587
H	7.047957	-3.106812	-3.720125
H	5.967493	-1.659730	-2.056953
H	-4.342490	-0.850217	-4.421011
H	-3.134652	0.432394	-6.157645
H	-0.630127	0.494123	-6.155962
H	0.649697	-0.710524	-4.447084
H	-4.307258	-2.262532	-2.416445
H	-4.314382	-3.664733	-0.417504
H	-3.078488	-4.887279	1.346494
H	-0.580371	-4.805052	1.416157
H	0.679946	-3.564955	-0.287060
H	-0.866701	0.985740	0.651617
H	-0.295658	1.366091	-2.375025
H	-2.805131	2.797367	1.292672
H	-4.695031	4.239310	1.567783
H	-3.395066	2.140364	2.831240
H	-5.635974	3.008718	2.447616
H	-4.548445	0.311275	1.725790
H	-8.026861	6.095366	0.342240
H	-7.420799	5.657703	-1.297932
H	-8.738203	4.696732	-0.545383
H	-3.149977	-0.684112	0.021422
H	-3.161076	-1.516486	2.175539
H	-0.028580	-3.141398	4.642880
C	1.970159	-1.472054	3.773628
H	0.826850	0.115061	1.935088
H	-5.330684	0.316319	-2.578858
H	-6.996799	-1.418789	-3.203815
H	-8.601445	-2.280283	-1.502395
H	-8.538126	-1.376758	0.822959
H	-6.876272	0.349420	1.439890
F	-2.239720	-4.641810	3.902375
F	-2.771727	-3.189854	5.392037
F	-3.876260	-3.286744	3.547940
F	2.197025	-0.467657	4.636412
F	2.341654	-2.599131	4.386221
F	2.804547	-1.284870	2.744432

diast-4l

Zero-point correction= 1.030196 (Hartree/Particle)
Thermal correction to Energy= 1.098778
Thermal correction to Enthalpy= 1.099722
Thermal correction to Gibbs Free Energy= 0.919163
Sum of electronic and zero-point Energies= -4342.399910
Sum of electronic and thermal Energies= -4342.331329
Sum of electronic and thermal Enthalpies= -4342.330384
Sum of electronic and thermal Free Energies= -4342.510943
Quasi-Harmonic Approximation corrected Free energy= -4342.491672
E(wB97XD/Def2TZVPP)= -4348.05625550

C	0.209377	4.410355	-3.195406
C	-0.814021	4.588882	-2.207247
C	-1.752056	3.524907	-1.975890
C	-1.603864	2.316306	-2.736550
C	-0.604938	2.179209	-3.657963
C	0.315672	3.242867	-3.895422
C	-2.762511	3.693276	-1.007228
C	-2.847349	4.879012	-0.253392
C	-1.901897	5.935045	-0.488026
C	-0.913257	5.767106	-1.462211
C	-1.998154	7.137223	0.286274
C	-2.967113	7.285061	1.237681
C	-3.905363	6.236353	1.474259
C	-3.848772	5.076081	0.754238
C	-3.740861	2.587692	-0.794528
C	-3.505294	1.601577	0.198822
C	-4.288624	0.476664	0.356179
C	-5.389297	0.282892	-0.548162
C	-5.687366	1.301199	-1.500196
C	-4.844683	2.439536	-1.597584
C	-6.790067	1.135079	-2.383192
C	-7.551977	-0.006379	-2.351686
C	-7.228795	-1.040875	-1.441723
C	-6.177849	-0.902508	-0.566755
O	-2.431268	1.798402	1.042465
P	-1.099656	0.908785	0.906008
O	-0.656193	0.829550	2.413074
C	-3.911689	-0.563937	1.353153
C	-2.645990	-1.115606	1.296194
C	-2.271340	-2.280646	2.016789
C	-3.178084	-2.800907	2.908024
C	-4.440704	-2.187809	3.129096
C	-4.820925	-1.060537	2.345118
C	-6.080093	-0.450102	2.600913
C	-6.921943	-0.948265	3.566404
C	-6.554773	-2.085764	4.325047
C	-5.339488	-2.688542	4.110868
O	-1.692268	-0.543131	0.480176
C	-0.985057	-2.978851	1.715948
C	0.151484	-2.796736	2.525982
C	1.356685	-3.522549	2.227096
C	1.372519	-4.423161	1.160681
C	0.245604	-4.623023	0.359171
C	-0.948771	-3.869595	0.620441
C	2.516312	-3.313279	3.044149
C	2.486144	-2.444592	4.098634
C	1.285708	-1.738273	4.408700
C	0.159824	-1.910878	3.654130
C	-2.072815	-4.072012	-0.247606
C	-2.019600	-4.972631	-1.274434
C	-0.843607	-5.742350	-1.508738
C	0.256101	-5.565884	-0.719276
O	-0.081168	1.350584	-0.071900
O	1.975178	0.885692	2.579548
C	2.919503	0.724183	1.847806
O	3.922267	1.580642	1.822972
C	3.148540	-0.418976	0.864702
N	1.982296	-0.559760	0.046200
C	1.841521	-1.522187	-0.920960
C	2.787563	-2.529503	-1.146977
C	2.581062	-3.475816	-2.153509
C	1.444485	-3.456544	-2.948537
C	0.493605	-2.458106	-2.709981
C	0.680146	-1.508092	-1.721928
H	1.360289	0.247663	-0.044827
H	-5.939307	-1.720209	0.115297
H	-7.816280	-1.963391	-1.438491
H	-8.394716	-0.126951	-3.038156
H	-7.010612	1.932074	-3.099664
H	-5.052925	3.189823	-2.365734
H	-2.922936	-3.706845	3.465509
H	-6.372057	0.433794	2.029267
H	-7.881743	-0.458436	3.753668
H	-7.236605	-2.475286	5.086253
H	-5.040112	-3.560942	4.699399
H	-0.196622	6.575508	-1.640972
H	2.286259	-4.984092	0.945598
H	-0.750337	-1.358761	3.897590
H	1.272772	-1.046807	5.255648
H	3.376179	-2.293247	4.716706
H	3.426884	-3.873039	2.806520
H	-2.984679	-3.492595	-0.086633
H	-2.885650	-5.100621	-1.929552
H	-0.822568	-6.458882	-2.334622
H	1.170768	-6.137436	-0.900361
H	-4.572797	4.279383	0.943963
H	-4.674525	6.365772	2.241202
H	-3.028450	8.206505	1.823944
H	-1.273916	7.936234	0.099519
H	0.909540	5.232362	-3.374156
H	1.108091	3.115279	-4.638491
H	-0.502158	1.241941	-4.211695
H	-2.292541	1.486306	-2.560995
H	0.329930	0.740730	2.536636
H	-0.072616	-0.738346	-1.545231
H	1.288929	-4.205297	-3.725179
H	3.693424	-2.585903	-0.542496
H	3.282047	-1.329535	1.482406
C	-0.738888	-2.374740	-3.572784
F	-1.787310	-1.870940	-2.916556
F	-0.536436	-1.581445	-4.639985
F	-1.101332	-3.569695	-4.052529
C	3.626079	-4.543142	-2.332785
F	4.839290	-4.019176	-2.554571
F	3.746213	-5.308501	-1.231244
F	3.355831	-5.365679	-3.346910
C	5.832418	2.654324	-1.158031
C	4.783467	2.460058	-0.254420
C	3.627140	3.232503	-0.368477
C	3.524560	4.195147	-1.373263
C	4.571538	4.389804	-2.271293
C	5.727846	3.614791	-2.160660
C	4.874492	1.332318	0.757206
C	4.469016	-0.052564	0.154339
C	5.669967	-0.972896	0.426406
C	6.435994	-0.297997	1.558613
N	6.168389	1.116582	1.331361
C	6.883654	2.056548	2.042005
O	7.870160	1.791055	2.685636
O	6.359659	3.273104	1.922771
C	7.043741	4.329721	2.573733
H	6.300983	-1.033092	-0.474246
H	7.517321	-0.488615	1.531539
H	5.369507	-1.998481	0.693018
H	6.062609	-0.610603	2.553468
H	4.278464	0.049883	-0.921257
H	7.081438	4.161481	3.662283
H	8.070248	4.432031	2.185552
H	6.466691	5.238476	2.353336
H	2.792722	3.098523	0.323225
H	2.611659	4.791779	-1.446958
H	4.488939	5.144477	-3.058994
H	6.556462	3.760696	-2.859773
H	6.741435	2.052088	-1.069777

ent-4l

Zero-point correction= 1.030006
(Hartree/Particle)
Thermal correction to Energy= 1.098726
Thermal correction to Enthalpy= 1.099670
Thermal correction to Gibbs Free Energy= 0.918819
Sum of electronic and zero-point Energies= -
4342.393973
Sum of electronic and thermal Energies= -
4342.325253
Sum of electronic and thermal Enthalpies= -
4342.324309
Sum of electronic and thermal Free Energies= -
4342.505160

Quasi-Harmonic Approximation corrected Free energy= -4342.485789
E(wB97XD/Def2TZVPP)= -4348.05271628

C	2.815121	-4.290269	-0.579363
C	2.047395	-3.357805	-1.350523
C	0.615768	-3.344055	-1.221677
C	0.030969	-4.219699	-0.244339
C	0.797448	-5.087856	0.480612
C	2.210240	-5.142683	0.297377
C	-0.149793	-2.480138	-2.040673
C	0.492167	-1.589636	-2.929248
C	1.927449	-1.557813	-2.987257
C	2.668511	-2.455066	-2.214949
C	2.574497	-0.614138	-3.851085
C	1.846128	0.234101	-4.635292
C	0.421576	0.171661	-4.619787
C	-0.228475	-0.710300	-3.804072
C	-1.638934	-2.552120	-1.973996
C	-2.422543	-1.440683	-1.558047
C	-3.799223	-1.473243	-1.442022
C	-4.468774	-2.726213	-1.646786
C	-3.710932	-3.842113	-2.105492
C	-2.308706	-3.714460	-2.280886
C	-4.366499	-5.081890	-2.345273
C	-5.711903	-5.223010	-2.111315
C	-6.458722	-4.127576	-1.614681
C	-5.855920	-2.913153	-1.389464
O	-1.769340	-0.260025	-1.300070
P	-1.632206	0.286443	0.216062
O	-1.462244	1.840009	-0.021165
C	-4.543176	-0.226072	-1.102199
C	-4.203081	0.510736	0.016565
C	-4.923871	1.656789	0.449271
C	-5.997174	2.062664	-0.305581
C	-6.346026	1.407952	-1.515888
C	-5.602715	0.268307	-1.939078
C	-5.922318	-0.311267	-3.199424
C	-6.945793	0.187645	-3.969353
C	-7.710143	1.292911	-3.525205
C	-7.410109	1.891519	-2.326616
O	-3.126212	0.124396	0.789627
C	-4.520068	2.383798	1.688079
C	-3.878151	3.635564	1.588518
C	-3.509675	4.337935	2.788142
C	-3.784332	3.766296	4.032502
C	-4.414445	2.523572	4.142105
C	-4.794849	1.817166	2.950490
C	-2.860849	5.612217	2.680212
C	-2.584197	6.158956	1.460357
C	-2.934896	5.458550	0.267402
C	-3.555941	4.243391	0.328291
C	-5.452843	0.551056	3.099953
C	-5.701222	0.024645	4.336413
C	-5.314955	0.725658	5.517028
C	-4.693520	1.937752	5.419958
O	-0.641545	-0.403093	1.066175
O	0.884332	1.478083	-1.175873
C	2.057420	1.598486	-0.926634
O	2.723724	2.716650	-1.159006
C	2.963208	0.562518	-0.278600
N	2.215775	-0.311081	0.562898
C	2.766904	-1.341206	1.273371
C	4.121743	-1.690210	1.180111
C	4.638079	-2.729857	1.957359
C	3.836327	-3.453291	2.826176
C	2.476957	-3.120549	2.894808
C	1.941892	-2.092167	2.141830
H	1.198740	-0.208460	0.607832
H	-6.446389	-2.083299	-0.995853
H	-7.524665	-4.250123	-1.402534
H	-6.205969	-6.181726	-2.292433
H	-3.772992	-5.926604	-2.707721
H	-1.741532	-4.583511	-2.626305
H	-6.578261	2.932681	0.013430
H	-5.337870	-1.156891	-3.566590
H	-7.167116	-0.271261	-4.937163
H	-8.526651	1.675248	-4.144209
H	-7.978566	2.760195	-1.981041
H	3.760935	-2.454844	-2.291650
H	-3.498374	4.302953	4.942884
H	-3.800968	3.713179	-0.593951
H	-2.695172	5.898760	-0.704913
H	-2.088564	7.131618	1.390002
H	-2.590743	6.137299	3.601725
H	-5.760471	0.001108	2.207233
H	-6.200832	-0.944322	4.425230
H	-5.520401	0.286817	6.497643
H	-4.395440	2.485303	6.319423
H	-1.319582	-0.746964	-3.818203
H	-0.153611	0.837223	-5.269540
H	2.348215	0.950651	-5.292086
H	3.668901	-0.593575	-3.872728
H	3.902106	-4.291917	-0.697948
H	2.805327	-5.845829	0.885990
H	0.326224	-5.735081	1.225067
H	-1.045021	-4.179606	-0.066857
H	-0.630243	2.032935	-0.518143
H	0.877824	-1.850708	2.192733
H	4.245599	-4.268138	3.425069
H	4.783686	-1.168983	0.486944
H	3.448909	0.013804	-1.113881
C	1.595276	-3.919266	3.819231
F	1.854568	-3.645313	5.105799
F	0.297349	-3.691456	3.620992
F	1.793803	-5.238670	3.666063
C	6.086789	-3.090653	1.768928
F	6.530430	-3.944909	2.692164
F	6.296284	-3.664025	0.570741
F	6.879234	-2.010137	1.811772
C	6.442763	2.495901	-1.385511
C	5.083297	2.505957	-1.714947
C	4.699212	2.317293	-3.041935
C	5.664717	2.107954	-4.028041
C	7.017772	2.090461	-3.695947
C	7.405535	2.289112	-2.369516
C	4.068337	2.670506	-0.596857
C	4.003246	1.476964	0.382979
C	3.557399	2.105345	1.704569
C	4.189739	3.495566	1.679515
N	4.281284	3.800630	0.249366
C	4.482765	5.057029	-0.266105
O	4.721696	5.931491	0.718607
C	4.923548	7.277684	0.321118
O	4.452344	5.338840	-1.437883
H	4.994224	1.013738	0.477295
H	2.457118	2.182535	1.742855
H	3.578482	4.247291	2.198783
H	5.195149	3.501408	2.136511
H	4.035093	7.673029	-0.197886
H	5.098486	7.841921	1.248003
H	5.798409	7.365049	-0.343527
H	3.872758	1.510845	2.574220
H	3.642444	2.347505	-3.313741
H	5.352813	1.965896	-5.066945
H	7.772721	1.928213	-4.470576
H	8.465427	2.284628	-2.099673
H	6.753955	2.657364	-0.348267

ent-diast-4l

Zero-point correction= 1.030554 (Hartree/Particle)
Thermal correction to Energy= 1.098812
Thermal correction to Enthalpy= 1.099756
Thermal correction to Gibbs Free Energy= 0.921221
Sum of electronic and zero-point Energies= -4342.397407
Sum of electronic and thermal Energies= -4342.329148

Sum of electronic and thermal Enthalpies= -4342.328204

Sum of electronic and thermal Free Energies= -4342.506739

Quasi-Harmonic Approximation corrected Free energy= -4342.488325

E(wB97XD/Def2TZVPP)= -4348.05416057

C	2.885494	-4.342492	-0.233211
C	1.761359	-4.006582	-1.058009
C	0.467353	-3.837020	-0.454199
C	0.389015	-3.926458	0.977435
C	1.484793	-4.241305	1.730906
C	2.750110	-4.478176	1.117709
C	-0.656729	-3.581441	-1.274865
C	-0.501995	-3.444134	-2.670677
C	0.807954	-3.544589	-3.252744
C	1.903247	-3.835847	-2.436300
C	0.960145	-3.368830	-4.667130
C	-0.119785	-3.126880	-5.466391
C	-1.426226	-3.059894	-4.897568
C	-1.610374	-3.216767	-3.552864
C	-2.002450	-3.460719	-0.639129
C	-2.702616	-2.223101	-0.630243
C	-3.880200	-2.006783	0.060129
C	-4.411736	-3.085232	0.845576
C	-3.773658	-4.358643	0.783913
C	-2.581726	-4.514267	0.028793
C	-4.308017	-5.445737	1.530258
C	-5.408638	-5.275427	2.332977
C	-6.014750	-4.000247	2.433822
C	-5.530929	-2.935298	1.712038
O	-2.166387	-1.179162	-1.351781
P	-1.391501	-0.014477	-0.545416
O	-1.471693	1.201173	-1.542825
C	-4.516789	-0.656972	0.024967
C	-3.772556	0.462063	0.348760
C	-4.310612	1.774306	0.434438
C	-5.647758	1.934025	0.163355
C	-6.455144	0.843672	-0.257401
C	-5.887344	-0.460218	-0.357917
C	-6.699761	-1.510859	-0.869195
C	-8.009814	-1.286229	-1.219008
C	-8.583487	-0.000081	-1.077095
C	-7.818614	1.040937	-0.611857
O	-2.428933	0.331616	0.631770
C	-3.421727	2.919027	0.791841
C	-3.064846	3.861566	-0.195781
C	-2.205173	4.960558	0.153014
C	-1.717298	5.065053	1.457077
C	-2.041635	4.122760	2.436406
C	-2.917813	3.033804	2.105504
C	-1.853183	5.919858	-0.852845
C	-2.302585	5.791492	-2.134998
C	-3.132778	4.687529	-2.492346
C	-3.500488	3.759260	-1.560127
C	-3.236096	2.091797	3.139828
C	-2.703671	2.209207	4.393170
C	-1.821259	3.283641	4.712210
C	-1.507483	4.213929	3.763100
O	-0.072100	-0.386848	0.004305
O	0.661830	0.784265	-3.028953
C	1.671489	1.158414	-2.478596
O	1.792014	2.375925	-1.980231
C	2.906752	0.310354	-2.171860
N	2.513863	-0.536244	-1.065387
C	3.375900	-0.987580	-0.093571
C	4.694692	-1.362925	-0.369414
C	5.530076	-1.803194	0.660206
C	5.077538	-1.887132	1.969481
C	3.752950	-1.520180	2.236531
C	2.910902	-1.078915	1.233673
H	-6.006131	-1.957813	1.813306
H	-6.872868	-3.858774	3.097034
H	-5.808231	-6.115939	2.907333
H	-3.813591	-6.419445	1.462659
H	-2.083320	-5.487848	0.027590
H	-6.096180	2.929105	0.234917
H	-6.271109	-2.506901	-0.996542
H	-8.612377	-2.108142	-1.615947
H	-9.629296	0.163569	-1.351953
H	-8.243621	2.044750	-0.517526
H	2.895460	-3.940093	-2.887347
H	-1.060063	5.900193	1.719441
H	-4.121411	2.911889	-1.856748
H	-3.470730	4.581652	-3.527248
H	-2.023206	6.526724	-2.895083
H	-1.210780	6.758924	-0.569367
H	-3.914697	1.264558	2.918535
H	-2.955414	1.473239	5.162091
H	-1.402300	3.358432	5.719776
H	-0.833432	5.043774	3.996270
H	-2.620583	-3.170917	-3.139598
H	-2.287962	-2.882733	-5.547554
H	0.008279	-2.993174	-6.544455
H	1.965707	-3.439055	-5.093677
H	3.859977	-4.480807	-0.711060
H	3.612854	-4.733239	1.738883
H	1.396779	-4.295913	2.819375
H	-0.562765	-3.727365	1.473054
H	-0.747262	1.212226	-2.231891
H	1.881559	-0.787021	1.451145
H	5.734414	-2.232351	2.769677
H	5.067512	-1.329892	-1.396355
H	3.201599	-0.265623	-3.067912
C	3.264364	-1.593039	3.658099
F	3.917898	-0.726608	4.447867
F	1.963810	-1.327582	3.774342
F	3.468448	-2.811862	4.184812
C	6.920330	-2.248301	0.295549
F	7.689723	-2.465677	1.363662
F	6.897467	-3.388832	-0.412697
F	7.546796	-1.340804	-0.467794
H	1.536590	-0.486027	-0.764850
C	1.457388	2.312814	0.740881
C	2.756679	2.428296	0.247162
C	3.832915	2.400081	1.139547
C	3.612740	2.230836	2.504814
C	2.311984	2.090658	2.991143
C	1.236406	2.136931	2.106236
C	3.044003	2.537282	-1.244009
C	3.919698	1.399135	-1.818898
C	4.614856	2.046823	-3.019388
C	4.832311	3.489099	-2.571749
N	3.713201	3.728821	-1.661859
C	3.385670	5.013646	-1.307541
O	2.266079	5.056228	-0.589410
C	1.883745	6.317851	-0.076015
O	4.039768	5.978370	-1.625573
H	4.658099	1.072907	-1.075540
H	5.554303	1.537050	-3.280265
H	5.791873	3.616953	-2.039409
H	4.812585	4.208260	-3.403691
H	1.029152	6.120797	0.585302
H	2.704870	6.775401	0.499437
H	1.587892	6.999945	-0.890038
H	3.959643	2.020657	-3.907844
H	4.857744	2.502332	0.768165
H	4.463474	2.190767	3.190680
H	2.138025	1.935501	4.059503
H	0.212306	2.026556	2.473829
H	0.607102	2.360500	0.060039

ReRe-cTC

Zero-point correction= 1.024401 (Hartree/Particle)

Thermal correction to Energy= 1.094733

Thermal correction to Enthalpy= 1.095677

Thermal correction to Gibbs Free Energy= 0.910305

Sum of electronic and zero-point Energies= -4342.345766

Sum of electronic and thermal Energies= -4342.275434

Sum of electronic and thermal Enthalpies= -4342.274490

Sum of electronic and thermal Free Energies= -4342.459862

Quasi-Harmonic Approximation corrected Free energy= -4342.439698

E(wB97XD/Def2TZVPP)= -4389.979314

C	2.542463	-4.416288	-0.068751
C	1.388972	-4.119917	-0.868617
C	0.140787	-3.819730	-0.220454
C	0.141821	-3.738446	1.214076
C	1.262816	-4.021859	1.941920
C	2.478937	-4.391052	1.294046
C	-1.018010	-3.611544	-1.003795
C	-0.941299	-3.649891	-2.412425
C	0.326126	-3.879987	-3.048965
C	1.456321	-4.121857	-2.263635
C	0.400037	-3.883360	-4.480127
C	-0.716057	-3.688144	-5.241095
C	-1.981554	-3.490279	-4.613971
C	-2.090717	-3.475984	-3.252531
C	-2.314888	-3.340272	-0.316906
C	-2.946338	-2.071980	-0.433845
C	-4.068862	-1.701476	0.278884
C	-4.612579	-2.636968	1.222525
C	-4.045532	-3.943079	1.297454
C	-2.907224	-4.264309	0.511527
C	-4.595396	-4.892711	2.202992
C	-5.642159	-4.557416	3.025781
C	-6.176203	-3.247097	2.986212
C	-5.676293	-2.312859	2.110644
O	-2.403620	-1.162189	-1.308718
P	-1.513779	0.043085	-0.699935
O	-1.499071	1.092477	-1.847846
C	-4.635349	-0.334885	0.086535
C	-3.824507	0.777553	0.228620
C	-4.313622	2.111190	0.183442
C	-5.651053	2.297970	-0.067814
C	-6.518795	1.204801	-0.327476
C	-6.011085	-0.125323	-0.269599
C	-6.887811	-1.195210	-0.605637
C	-8.199164	-0.956819	-0.940802
C	-8.710726	0.362935	-0.958156
C	-7.884487	1.418876	-0.662702
O	-2.472844	0.619877	0.462169
C	-3.399390	3.264275	0.436424
C	-2.930234	4.044096	-0.640396
C	-2.067833	5.164874	-0.378400
C	-1.706630	5.463283	0.937127
C	-2.155746	4.689299	2.010439
C	-3.018588	3.567783	1.760967
C	-1.575189	5.938753	-1.479879
C	-1.911751	5.623530	-2.764744
C	-2.764843	4.510493	-3.028616
C	-3.255891	3.749113	-2.006545
C	-3.448995	2.786773	2.884852
C	-3.040686	3.088845	4.153817
C	-2.177645	4.198456	4.396528
C	-1.752996	4.974526	3.355780
O	-0.219821	-0.405321	-0.118541
O	0.356133	0.194617	-3.202219
C	1.535407	0.613095	-3.287564
O	2.025565	1.569506	-3.869257
C	2.554272	-0.135452	-2.428264
N	2.208831	-0.554089	-1.266600
C	3.057494	-1.019558	-0.226756
C	4.314879	-1.554578	-0.483710
C	5.107276	-1.960422	0.586563
C	4.652442	-1.832124	1.894795
C	3.384594	-1.302602	2.128382
C	2.575172	-0.898633	1.075692
H	-6.095339	-1.304821	2.102114
H	-6.991390	-2.973240	3.661994
H	-6.054417	-5.293153	3.722024
H	-4.157734	-5.894852	2.240355
H	-2.462301	-5.258399	0.612322
H	-6.054694	3.314221	-0.094711
H	-6.508661	-2.219025	-0.610088
H	-8.851436	-1.794791	-1.202642
H	-9.757931	0.538170	-1.220265
H	-8.261364	2.445641	-0.691705
H	2.412343	-4.334325	-2.753905
H	-1.040414	6.309817	1.130298
H	-3.889565	2.887820	-2.226503
H	-3.015479	4.258887	-4.063001
H	-1.524459	6.216023	-3.598575
H	-0.915575	6.785232	-1.266033
H	-4.112564	1.934595	2.719034
H	-3.377362	2.475150	4.994365
H	-1.860540	4.423144	5.418972
H	-1.090392	5.827852	3.529592
H	-3.070440	-3.329584	-2.792397
H	-2.872198	-3.348253	-5.232576
H	-0.648008	-3.688910	-6.332653
H	1.373744	-4.050148	-4.951082
H	3.478066	-4.668035	-0.577182
H	3.362416	-4.628581	1.893325
H	1.233169	-3.953079	3.032675
H	-0.770159	-3.436041	1.731381
H	-0.764381	0.860330	-2.554888
H	1.574868	-0.494406	1.246249
H	5.280014	-2.151633	2.729665
H	4.676498	-1.679855	-1.506334
H	3.607163	-0.160871	-2.729583
C	2.905461	-1.155868	3.550819
F	3.551265	-0.160531	4.179615
F	1.604832	-0.893789	3.623679
F	3.137842	-2.268694	4.258008
C	6.432351	-2.621098	0.305811
F	7.272637	-2.516843	1.337315
F	6.276910	-3.927604	0.053118
F	7.035071	-2.087651	-0.761847
H	1.189625	-0.473537	-0.986651
C	2.548957	2.592710	-0.607765
C	3.261887	2.441863	0.590828
C	2.548371	2.460836	1.798995
C	1.161245	2.592583	1.805144
C	0.464460	2.724156	0.604452
C	1.164529	2.739369	-0.600785
C	4.710417	2.153094	0.589707
C	5.395739	1.457739	1.514478
C	6.814195	1.218110	1.069781
C	6.738424	1.561214	-0.427148
N	5.550947	2.418195	-0.532555
C	5.631511	3.580169	-1.261205
O	4.722489	4.478519	-0.877854
C	4.585254	5.629680	-1.690569
O	6.437421	3.763025	-2.144786
H	4.971856	1.075923	2.442401
H	7.525883	1.870124	1.612752
H	7.617288	2.090693	-0.817838
H	6.593244	0.647950	-1.031263
H	3.765673	6.212753	-1.247243
H	5.513260	6.224542	-1.694345
H	4.327880	5.351685	-2.726074
H	7.140310	0.178580	1.233823
H	3.083348	2.367323	2.747942
H	0.617056	2.588966	2.753983
H	-0.624087	2.811058	0.607562
H	0.627655	2.857094	-1.546093
H	3.071841	2.581076	-1.566982

ReRe-cTS

Zero-point correction= 1.025967 (Hartree/Particle)

Thermal correction to Energy= 1.095094
Thermal correction to Enthalpy= 1.096038
Thermal correction to Gibbs Free Energy= 0.915501
Sum of electronic and zero-point Energies= -
4342.323364
Sum of electronic and thermal Energies= -
4342.254237
Sum of electronic and thermal Enthalpies= -
4342.253293
Sum of electronic and thermal Free Energies= -
4342.433829
Quasi-Harmonic Approximation corrected Free energy= -4342.415368
E(wB97XD/Def2TZVPP)= -4347.98477270

C	3.498975	-4.075957	-1.005296
C	2.489387	-3.359581	-1.729300
C	1.111907	-3.496254	-1.340446
C	0.830668	-4.282853	-0.170919
C	1.820814	-4.948531	0.495005
C	3.176496	-4.864178	0.061049
C	0.105433	-2.862760	-2.108173
C	0.455418	-2.061027	-3.216625
C	1.841692	-1.857709	-3.536315
C	2.822264	-2.524284	-2.797396
C	2.191898	-0.993670	-4.623961
C	1.228075	-0.387795	-5.375614
C	-0.148944	-0.630250	-5.097839
C	-0.521478	-1.438629	-4.061908
C	-1.324840	-3.076641	-1.741440
C	-2.157435	-1.996499	-1.335041
C	-3.461011	-2.154278	-0.906447
C	-3.980257	-3.487043	-0.779610
C	-3.187932	-4.577626	-1.240992
C	-1.878931	-4.336001	-1.732833
C	-3.702982	-5.901326	-1.158546
C	-4.938291	-6.143103	-0.610323
C	-5.707634	-5.066085	-0.108173
C	-5.243554	-3.774893	-0.190778
O	-1.643854	-0.725582	-1.400713
P	-1.222796	0.022079	-0.027836
O	-1.148409	1.523169	-0.447743
C	-4.279445	-0.953885	-0.571157
C	-3.814377	-0.012337	0.330453
C	-4.604222	1.078257	0.791092
C	-5.874105	1.211918	0.281573
C	-6.369634	0.347208	-0.727413
C	-5.560219	-0.731558	-1.184428
C	-6.048268	-1.528379	-2.258486
C	-7.283392	-1.290116	-2.812290
C	-8.104163	-0.245123	-2.324115
C	-7.651517	0.558203	-1.307067
O	-2.554110	-0.138454	0.870268
C	-4.085050	2.041869	1.804646
C	-3.896704	3.395834	1.447954
C	-3.476320	4.340124	2.448365
C	-3.211250	3.896467	3.745832
C	-3.375269	2.556515	4.105847
C	-3.839783	1.612308	3.127207
C	-3.341000	5.723265	2.094451
C	-3.596995	6.153110	0.824043
C	-3.976624	5.214772	-0.180991
C	-4.107637	3.886841	0.114747
C	-4.054971	0.258928	3.553038
C	-3.796424	-0.129928	4.837055
C	-3.309141	0.805260	5.797574
C	-3.110858	2.108199	5.441532
O	-0.059458	-0.577260	0.670487
O	0.991665	1.353473	-1.730839
C	2.226238	1.441171	-1.925153
O	2.857149	1.993728	-2.820875
C	3.104198	0.748624	-0.872988
N	2.599226	-0.270533	-0.191937
C	3.309372	-1.135038	0.657080
C	4.675755	-1.364991	0.500257
C	5.345602	-2.199504	1.391528
C	4.664349	-2.837532	2.421404
C	3.290333	-2.628951	2.546296
C	2.608736	-1.783917	1.680807
H	-5.847558	-2.959351	0.211694
H	-6.678631	-5.263140	0.355139
H	-5.323092	-7.164767	-0.545456
H	-3.086986	-6.726436	-1.528706
H	-1.277982	-5.187246	-2.065348
H	-6.509632	2.028749	0.635651
H	-5.425887	-2.331111	-2.658371
H	-7.632187	-1.910788	-3.642556
H	-9.088293	-0.071983	-2.768636
H	-8.265321	1.383696	-0.934180
H	3.875382	-2.397890	-3.070162
H	-2.878490	4.616668	4.500127
H	-4.369911	3.182214	-0.676438
H	-4.151420	5.561668	-1.203594
H	-3.495107	7.211342	0.567063
H	-3.028542	6.429251	2.869978
H	-4.435864	-0.472804	2.837243
H	-3.964970	-1.168860	5.134531
H	-3.103545	0.473582	6.819409
H	-2.748698	2.837884	6.172336
H	-1.582470	-1.613627	-3.872733
H	-0.914414	-0.157834	-5.720017
H	1.505850	0.282172	-6.193813
H	3.251817	-0.821472	-4.832154
H	4.539955	-3.972124	-1.325386
H	3.954505	-5.409081	0.603081
H	1.574938	-5.538312	1.382122
H	-0.193532	-4.340917	0.200662
H	-0.286761	1.680090	-0.983302
H	1.532165	-1.623067	1.772115
H	5.191079	-3.501938	3.108800
H	5.226655	-0.907422	-0.324044
H	4.175285	0.744318	-1.079484
C	2.550773	-3.338459	3.653065
F	2.832641	-2.805550	4.848988
F	1.229911	-3.288948	3.496140
F	2.899616	-4.631385	3.717507
C	6.815089	-2.442774	1.165446
F	7.386309	-3.093312	2.178340
F	7.022894	-3.169012	0.057591
F	7.480781	-1.291683	0.999505
H	1.566528	-0.307014	-0.103647
C	1.771875	4.611423	-1.827124
C	1.964642	4.139306	-0.519425
C	0.885619	4.132961	0.375994
C	-0.357766	4.616370	-0.017564
C	-0.538656	5.090109	-1.315728
C	0.524391	5.080227	-2.219693
C	3.237477	3.537904	-0.106336
C	3.353556	2.348879	0.601657
C	4.800074	2.203972	1.031489
C	5.518674	3.039410	-0.031500
N	4.488682	4.009530	-0.435323
C	4.899370	5.216910	-0.993256
O	3.964106	6.150253	-0.926510
C	4.235472	7.378580	-1.584612
O	6.011459	5.374674	-1.427329
H	2.509935	1.963726	1.175658
H	4.942999	2.638069	2.038887
H	6.410041	3.565762	0.334229
H	5.811105	2.438905	-0.910419
H	3.328219	7.986389	-1.464478
H	5.097598	7.886781	-1.123579
H	4.438458	7.210393	-2.654853
H	5.173904	1.171081	1.067052
H	1.020742	3.757825	1.393831
H	-1.194197	4.605328	0.684782
H	-1.521595	5.452977	-1.628119
H	0.376506	5.425282	-3.246646
H	2.585001	4.563116	-2.551626

ReSi-cTC

Zero-point correction= 1.025404
(Hartree/Particle)
Thermal correction to Energy= 1.095313
Thermal correction to Enthalpy= 1.096257
Thermal correction to Gibbs Free Energy= 0.915594
Sum of electronic and zero-point Energies= -
4342.355703
Sum of electronic and thermal Energies= -
4342.285794
Sum of electronic and thermal Enthalpies= -
4342.284850
Sum of electronic and thermal Free Energies= -
4342.465514
Quasi-Harmonic Approximation corrected Free energy= -4342.448045
E(wB97XD/Def2TZVPP)= -4348.01295485

C	5.364769	1.673840	-1.683400
C	4.874644	1.729483	-0.369233
C	5.465792	0.900267	0.597758
C	6.504532	0.037109	0.251456
C	6.975291	-0.016438	-1.060653
C	6.399958	0.808061	-2.027673
C	3.700713	2.578004	-0.072519
C	2.700372	2.867058	-0.927295
C	1.636357	3.690874	-0.257803
C	1.991308	3.543946	1.228928
N	3.389414	3.073362	1.218463
C	4.292115	3.405962	2.195964
O	5.495260	3.322486	2.111396
O	3.649967	3.849274	3.283734
C	4.451104	4.091689	4.423263
O	-0.243973	1.260432	0.208944
P	-1.270021	0.575534	1.043542
O	-1.396644	-0.943334	0.476033
C	-2.342824	-1.770113	1.038994
C	-3.676745	-1.553246	0.761289
C	-4.646525	-2.343192	1.463476
C	-4.195217	-3.394314	2.314680
C	-2.801739	-3.626033	2.473011
C	-1.868979	-2.819209	1.868350
C	-6.044918	-2.101134	1.368185
C	-6.944145	-2.879353	2.057229
C	-6.496378	-3.943333	2.876585
C	-5.151483	-4.190466	3.003562
C	-4.037396	-0.533581	-0.263729
C	-3.552652	0.756490	-0.159595
C	-3.800884	1.766230	-1.126022
C	-4.598713	1.445068	-2.197730
C	-5.101717	0.132022	-2.385986
C	-4.791850	-0.883512	-1.435520
C	-5.211337	-2.214815	-1.716644
C	-5.935739	-2.504966	-2.847954
C	-6.282228	-1.484448	-3.765505
C	-5.866164	-0.196038	-3.539958
O	-2.781931	1.105610	0.934192
C	-3.163635	3.110807	-1.018187
C	-2.063701	3.413469	-1.848144
C	-1.488799	4.730175	-1.806619
C	-2.000326	5.675533	-0.914456
C	-3.067092	5.373823	-0.062994
C	-3.669661	4.070708	-0.119684
C	-0.390970	5.040048	-2.675082
C	0.132838	4.096488	-3.510884
C	-0.409797	2.776828	-3.527385
C	-1.470024	2.448542	-2.730281
C	-4.777758	3.799789	0.749661
C	-5.240530	4.747212	1.618965
C	-4.634750	6.037548	1.679130
C	-3.583596	6.339732	0.861295
C	-0.399217	-3.021506	2.026893
C	0.342289	-3.561142	0.952965
C	1.755023	-3.772280	1.104112
C	2.374082	-3.434413	2.312150
C	1.654314	-2.875596	3.372604
C	0.238907	-2.660299	3.231028
C	2.501069	-4.317074	0.008764
C	1.890280	-4.628997	-1.172192
C	0.488329	-4.420341	-1.327431
C	-0.258532	-3.910565	-0.302400
C	-0.462683	-2.053260	4.325597
C	0.194172	-1.690647	5.466493
C	1.595373	-1.915208	5.606055
C	2.301329	-2.495003	4.592845
O	-1.026749	0.536831	2.573363
O	1.392942	0.698721	3.085917
C	2.553883	0.327872	2.802483
O	3.605850	0.480774	3.410286
C	2.756049	-0.416665	1.475420
N	2.097071	-0.137330	0.408486
C	2.261246	-0.734040	-0.875544
C	3.470116	-1.295440	-1.276609
C	3.542125	-1.921911	-2.517334
C	2.430536	-1.980583	-3.354278
C	1.235362	-1.395588	-2.942411
C	1.143138	-0.767159	-1.707393
C	4.826478	-2.583301	-2.954327
H	-1.871134	1.433416	-2.756216
H	0.028273	2.021178	-4.185037
H	0.975411	4.343091	-4.163336
H	0.027273	6.050722	-2.642819
H	-1.554150	6.674520	-0.878634
H	-3.114678	7.327831	0.897997
H	-5.018806	6.781805	2.382645
H	-6.083348	4.517462	2.277198
H	-5.254050	2.816813	0.713361
H	-4.945155	-3.020821	-1.030898
H	-6.241766	-3.536634	-3.043407
H	-6.864848	-1.728620	-4.658194
H	-6.104177	0.599319	-4.252572
H	-4.813361	2.207448	-2.952026
H	-4.793526	-5.000088	3.646559
H	-2.469994	-4.451134	3.109801
H	-7.222826	-4.558299	3.415174
H	-8.014798	-2.671398	1.975871
H	-6.404092	-1.277898	0.746607
H	3.379338	-2.655053	4.684481
H	2.102672	-1.607954	6.524490
H	-0.355891	-1.209970	6.280187
H	-1.531640	-1.855984	4.227426
H	3.449017	-3.608180	2.429863
H	3.577682	-4.469644	0.134689
H	2.473199	-5.033636	-2.004189
H	0.009080	-4.668480	-2.278317
H	-1.331865	-3.762636	-0.441719
H	1.311331	0.557202	0.459088
H	-0.006870	0.566205	2.821841
H	0.622811	3.318973	-0.465529
H	1.353205	2.795541	1.727550
H	1.683057	4.743950	-0.595561
H	1.914866	4.482595	1.791941
H	2.655948	2.534536	-1.965050
H	3.765981	4.462930	5.198895
H	4.929606	3.159738	4.765171
H	5.227551	4.846195	4.214299
H	3.546049	-1.169722	1.424252
H	4.353971	-1.244854	-0.637726
H	2.490658	-2.495498	-4.315889
C	0.023114	-1.469979	-3.838070
H	0.207266	-0.318240	-1.375406
H	5.119825	0.924045	1.632602
H	6.953603	-0.596175	1.022526
H	7.789091	-0.695641	-1.329300
H	6.763303	0.781921	-3.058531
H	4.938954	2.331844	-2.445629
F	4.580339	-3.727205	-3.605633
F	5.533727	-1.810769	-3.783622
F	5.611435	-2.880937	-1.916013
F	0.102518	-0.591256	-4.846792
F	-0.098988	-2.681248	-4.391390
F	-1.106221	-1.214278	-3.176865

ReSi-cTS

Zero-point correction= 1.026058 (Hartree/Particle)
Thermal correction to Energy= 1.095083
Thermal correction to Enthalpy= 1.096028
Thermal correction to Gibbs Free Energy= 0.916753
Sum of electronic and zero-point Energies= -4342.341531
Sum of electronic and thermal Energies= -4342.272505
Sum of electronic and thermal Enthalpies= -4342.271561
Sum of electronic and thermal Free Energies= -4342.450836
Quasi-Harmonic Approximation corrected Free energy= -4342.432987
E(wB97XD/Def2TZVPP)= -4347.99992786

C	6.356727	-0.139924	-1.581082
C	5.959147	0.558988	-0.433858
C	6.208607	0.006516	0.831034
C	6.857080	-1.220709	0.936909
C	7.265231	-1.906610	-0.209314
C	7.016485	-1.363472	-1.468587
C	5.107139	1.758598	-0.590336
C	3.930300	1.728049	-1.298428
C	3.292342	3.090968	-1.317287
C	4.137920	3.878481	-0.298641
N	5.273312	2.981729	0.008243
C	6.280843	3.340880	0.890246
O	7.250171	2.679478	1.156937
O	6.035808	4.556588	1.374629
C	6.977734	5.062411	2.307511
O	-0.753542	1.396626	-0.669612
P	-1.458792	1.133119	0.609339
O	-1.484616	-0.482986	0.816099
C	-2.203118	-1.021765	1.855973
C	-3.583072	-1.017112	1.799955
C	-4.306756	-1.481309	2.948306
C	-3.582869	-2.046691	4.038525
C	-2.164307	-2.107703	3.986042
C	-1.461853	-1.582635	2.928492
C	-5.721188	-1.373587	3.058733
C	-6.379455	-1.832025	4.174708
C	-5.662185	-2.425383	5.241298
C	-4.294268	-2.524894	5.173747
C	-4.251640	-0.544253	0.555490
C	-3.933714	0.693088	0.029300
C	-4.491907	1.197391	-1.175631
C	-5.424694	0.425671	-1.824248
C	-5.770045	-0.870487	-1.360472
C	-5.154990	-1.384092	-0.181890
C	-5.432720	-2.730897	0.186852
C	-6.305421	-3.500131	-0.544977
C	-6.950448	-2.970537	-1.688060
C	-6.680925	-1.685380	-2.088203
O	-3.025474	1.496958	0.690439
C	-4.009365	2.488802	-1.743930
C	-3.091288	2.461611	-2.814026
C	-2.610877	3.700021	-3.362552
C	-3.058878	4.909673	-2.825925
C	-3.963901	4.946082	-1.761195
C	-4.450694	3.713668	-1.206433
C	-1.673836	3.664495	-4.447375
C	-1.231104	2.477181	-4.955942
C	-1.697092	1.245368	-4.406634
C	-2.590657	1.237953	-3.373679
C	-5.376981	3.782599	-0.113640
C	-5.784449	4.985435	0.391120
C	-5.298984	6.208008	-0.161418
C	-4.418408	6.186408	-1.205190
C	0.029163	-1.589337	2.877941
C	0.696620	-2.476753	2.005989
C	2.133126	-2.494977	1.978319
C	2.847174	-1.634723	2.818974
C	2.196709	-0.750062	3.681523
C	0.759616	-0.710692	3.703712
C	2.802376	-3.399568	1.091430
C	2.095616	-4.242468	0.280989
C	0.670851	-4.226603	0.303598
C	-0.003563	-3.376555	1.135944
C	0.125179	0.247576	4.564330
C	0.865045	1.083953	5.350315
C	2.290382	1.024208	5.342526
C	2.933599	0.136850	4.531286
O	-0.902310	1.803115	1.900348
O	1.537666	2.102354	1.413380
C	2.662382	1.548772	1.345879
O	3.679058	1.745777	2.005365
C	2.796540	0.488567	0.238892
N	1.730246	0.119463	-0.438396
C	1.667240	-0.947909	-1.348799
C	2.809537	-1.608624	-1.796311
C	2.682894	-2.670687	-2.692977
C	1.435978	-3.089597	-3.136500
C	0.298762	-2.416470	-2.680031
C	0.404530	-1.350733	-1.801993
C	3.943549	-3.363559	-3.142051
H	-2.927396	0.286605	-2.957367
H	-1.329046	0.298492	-4.811699
H	-0.515977	2.463443	-5.783753
H	-1.318867	4.614682	-4.858423
H	-2.688559	5.850778	-3.245057
H	-4.040478	7.119058	-1.635311
H	-5.636397	7.160848	0.256585
H	-6.488708	5.015261	1.227572
H	-5.759182	2.853983	0.317947
H	-4.937086	-3.164334	1.057271
H	-6.497531	-4.533972	-0.244135
H	-7.647568	-3.591521	-2.257684
H	-7.152522	-1.270725	-2.984203
H	-5.875873	0.795066	-2.749655
H	-3.727431	-2.965267	5.999547
H	-1.621622	-2.556300	4.823118
H	-6.198688	-2.791021	6.121395
H	-7.466546	-1.732113	4.241701
H	-6.287652	-0.908704	2.248887
H	4.025399	0.101844	4.496294
H	2.862972	1.707649	5.975644
H	0.361250	1.817053	5.986794
H	-0.963795	0.320162	4.568493
H	3.941435	-1.654594	2.801335
H	3.896890	-3.398703	1.072355
H	2.619879	-4.925203	-0.392963
H	0.113253	-4.895391	-0.358346
H	-1.096310	-3.379669	1.138818
H	0.900000	0.743061	-0.411802
H	0.116172	1.966292	1.820926
H	2.226674	3.066107	-1.033317
H	3.587672	4.088204	0.628976
H	3.349373	3.532920	-2.327412
H	4.518993	4.829083	-0.698906
H	3.724162	0.958976	-2.043872
H	6.610342	6.057550	2.593760
H	7.033667	4.412241	3.195409
H	7.977698	5.144681	1.851328
H	3.612870	-0.230369	0.333372
H	3.805847	-1.313671	-1.456338
H	1.344713	-3.932212	-3.824465
C	-1.060875	-2.873687	-3.145053
H	-0.482669	-0.827912	-1.445261
H	5.856401	0.530913	1.720484
H	7.043823	-1.647616	1.926588
H	7.773809	-2.870807	-0.119612
H	7.324040	-1.898473	-2.370673
H	6.151524	0.278222	-2.570722
F	3.698823	-4.383807	-3.964423
F	4.762738	-2.519917	-3.784432
F	4.632349	-3.847805	-2.099464

F	-1.282396	-2.538282	-4.424024
F	-1.175940	-4.205156	-3.070248
F	-2.048402	-2.345814	-2.421036

SiRe-cTC

Zero-point correction= 1.025069 (Hartree/Particle)
Thermal correction to Energy= 1.094912
Thermal correction to Enthalpy= 1.095857
Thermal correction to Gibbs Free Energy= 0.915413
Sum of electronic and zero-point Energies= -4342.354440
Sum of electronic and thermal Energies= -4342.284597
Sum of electronic and thermal Enthalpies= -4342.283652
Sum of electronic and thermal Free Energies= -4342.464096
Quasi-Harmonic Approximation corrected Free energy= -4342.446436
E(wB97XD/Def2TZVPP)= -4348.01249861

C	-3.539679	-3.825125	0.519858
C	-2.289536	-3.647550	1.201059
C	-1.067504	-3.643520	0.442115
C	-1.179032	-3.718823	-0.988632
C	-2.389021	-3.887930	-1.601206
C	-3.589896	-3.968276	-0.836356
C	0.171782	-3.558799	1.120594
C	0.206687	-3.447046	2.527741
C	-1.023667	-3.362661	3.264861
C	-2.239903	-3.473991	2.586420
C	-0.980669	-3.179395	4.685159
C	0.211077	-3.115562	5.345775
C	1.434516	-3.251968	4.626226
C	1.433508	-3.413834	3.270195
C	1.437479	-3.557914	0.330896
C	2.290512	-2.419345	0.333441
C	3.409504	-2.290645	-0.463307
C	3.708721	-3.346416	-1.388406
C	2.914619	-4.529958	-1.355639
C	1.800185	-4.609000	-0.478663
C	3.219717	-5.599686	-2.242526
C	4.246608	-5.492911	-3.147779
C	5.007301	-4.300895	-3.212334
C	4.746752	-3.256663	-2.357199
O	1.974416	-1.375315	1.173219
P	1.245233	-0.105161	0.491891
O	1.262067	0.990422	1.584017
C	4.237869	-1.055588	-0.349238
C	3.654029	0.194518	-0.468251
C	4.394296	1.405611	-0.433063
C	5.754470	1.324749	-0.253546
C	6.405713	0.082631	-0.042527
C	5.643967	-1.121009	-0.061014
C	6.310317	-2.341175	0.247096
C	7.658604	-2.364138	0.511963
C	8.420376	-1.171190	0.484940
C	7.802878	0.025600	0.219492
O	2.291696	0.310334	-0.662170
C	3.729568	2.733623	-0.583452
C	3.586241	3.568081	0.544089
C	3.062125	4.896784	0.379657
C	2.679289	5.331829	-0.890251
C	2.770235	4.495536	-2.006515
C	3.305816	3.171247	-1.856055
C	2.937071	5.744971	1.528531
C	3.300045	5.304895	2.768626
C	3.794130	3.976786	2.940330
C	3.930576	3.139306	1.870143
C	3.388431	2.342078	-3.023584
C	2.949305	2.786501	-4.239373
C	2.408104	4.098368	-4.382619
C	2.327315	4.926693	-3.299319
O	-0.073987	-0.441617	-0.113963
O	-0.433706	0.446551	3.256872
C	-1.657761	0.257935	3.468812
O	-2.227719	-0.061130	4.501554
C	-2.654810	0.325063	2.318495
N	-2.425201	-0.217132	1.172915
C	-3.376523	-0.475065	0.149264
C	-4.725015	-0.660073	0.439680
C	-5.608691	-0.940600	-0.597284
C	-5.155954	-1.048807	-1.909816
C	-3.800961	-0.869103	-2.176642
C	-2.902720	-0.583896	-1.156147
H	-1.439166	-0.438762	0.892840
H	5.338250	-2.341586	-2.427589
H	5.807002	-4.207545	-3.952628
H	4.469524	-6.318946	-3.828951
H	2.609226	-6.506704	-2.198519
H	1.186402	-5.514227	-0.490143
H	6.347453	2.243680	-0.236505
H	5.739646	-3.270732	0.287208
H	8.146031	-3.313204	0.752617
H	9.493882	-1.205015	0.690952
H	8.373971	0.958881	0.217596
H	-3.173477	-3.438771	3.157315
H	2.279101	6.343490	-1.011461
H	4.292225	2.120285	2.021668
H	4.055057	3.624697	3.942415
H	3.202031	5.961511	3.638058
H	2.542224	6.755782	1.388197
H	3.804237	1.335660	-2.934414
H	3.012849	2.132053	-5.113418
H	2.061880	4.436266	-5.363598
H	1.917085	5.936387	-3.398710
H	2.382457	-3.521465	2.740366
H	2.383738	-3.221908	5.168820
H	0.234085	-2.963415	6.428338
H	-1.925241	-3.078531	5.225725
H	-4.457589	-3.849665	1.115396
H	-4.548838	-4.113624	-1.341728
H	-2.440956	-3.945339	-2.692088
H	-0.278867	-3.631070	-1.598923
H	0.482650	0.835191	2.305595
H	-1.836986	-0.461249	-1.354536
H	-5.854447	-1.275898	-2.718419
H	-5.089984	-0.610800	1.466961
H	-3.665196	0.663750	2.557078
C	-3.315989	-0.934936	-3.601610
F	-3.654790	0.175104	-4.276255
F	-1.992939	-1.057282	-3.681553
F	-3.859017	-1.966362	-4.258078
C	-7.060911	-1.200479	-0.289095
F	-7.857768	-0.772726	-1.273452
F	-7.296021	-2.511007	-0.136272
F	-7.449806	-0.596977	0.834833
C	-4.200117	4.185470	0.453789
C	-3.813385	3.160861	-0.423051
C	-4.815706	2.410436	-1.052161
C	-6.163504	2.650914	-0.793702
C	-6.534761	3.658628	0.095275
C	-5.546692	4.430454	0.708880
C	-2.395228	2.914257	-0.761637
C	-1.906830	2.552316	-1.961682
C	-0.401102	2.525213	-1.944627
C	-0.082308	3.305512	-0.660103
N	-1.320240	3.190984	0.124896
C	-1.352802	3.194836	1.484643
O	-0.236841	3.722518	1.982819
C	-0.097578	3.739116	3.395591
O	-2.278207	2.795182	2.172459
H	-2.520020	2.346369	-2.839795
H	-0.018983	1.488398	-1.903189
H	0.771077	2.912819	-0.091967
H	0.110235	4.369342	-0.880364
H	-0.101338	2.716737	3.802907
H	0.872655	4.217182	3.586741
H	-0.907945	4.325464	3.859222

H	0.050041	3.003302	-2.828846
H	-4.534726	1.620129	-1.750766
H	-6.926172	2.039570	-1.285163
H	-7.591511	3.846291	0.306634
H	-5.826767	5.233227	1.397143
H	-3.444792	4.809071	0.936071

SiRe-cTS

```
 Zero-point correction=                           1.025649
(Hartree/Particle)
 Thermal correction to Energy=                    1.094737
 Thermal correction to Enthalpy=                  1.095682
 Thermal correction to Gibbs Free Energy=         0.914956
 Sum of electronic and zero-point Energies=          -
4342.330863
 Sum of electronic and thermal Energies=             -
4342.261775
 Sum of electronic and thermal Enthalpies=           -
4342.260831
 Sum of electronic and thermal Free Energies=        -
4342.441556
Quasi-Harmonic Approximation corrected Free energy= -4342.422900
E(wB97XD/Def2TZVPP)= -4347.98983511
```

C	2.327856	-4.656899	-0.772605
C	1.390208	-3.894155	-1.544635
C	0.034860	-3.762146	-1.080798
C	-0.284868	-4.334969	0.197408
C	0.637596	-5.051055	0.907000
C	1.960636	-5.236052	0.407101
C	-0.911854	-3.079132	-1.880316
C	-0.517494	-2.493359	-3.103220
C	0.857436	-2.563441	-3.514957
C	1.773550	-3.272370	-2.734510
C	1.260129	-1.917160	-4.728965
C	0.353213	-1.258530	-5.507395
C	-1.020418	-1.223904	-5.126263
C	-1.440079	-1.821525	-3.972044
C	-2.325676	-2.977594	-1.411014
C	-2.894757	-1.713365	-1.091993
C	-4.163434	-1.547867	-0.569615
C	-4.928503	-2.723666	-0.266165
C	-4.404347	-3.997829	-0.632918
C	-3.112499	-4.088631	-1.215572
C	-5.166479	-5.169362	-0.366694
C	-6.382939	-5.090142	0.265109
C	-6.885165	-3.829867	0.669362
C	-6.179113	-2.679119	0.411363
O	-2.137808	-0.595424	-1.336779
P	-1.455401	0.180086	-0.090476
O	-1.187357	1.601825	-0.670186
C	-4.671353	-0.167627	-0.313051
C	-3.923574	0.714246	0.445664
C	-4.353266	2.028026	0.777664
C	-5.576275	2.440413	0.308922
C	-6.365018	1.614190	-0.534968
C	-5.904421	0.309097	-0.876961
C	-6.674544	-0.453908	-1.799505
C	-7.851918	0.034328	-2.313958
C	-8.328531	1.312664	-1.937470
C	-7.595099	2.085618	-1.071652
O	-2.693550	0.323754	0.931921
C	-3.480536	2.911608	1.606564
C	-2.758699	3.958987	0.997090
C	-1.910283	4.795109	1.803201
C	-1.809820	4.555056	3.175488
C	-2.517869	3.517684	3.788754
C	-3.371965	2.679561	2.993770
C	-1.174602	5.854898	1.176508
C	-1.260619	6.069804	-0.168879
C	-2.090147	5.233632	-0.974453
C	-2.811001	4.217891	-0.413908
C	-4.087536	1.627111	3.656058
C	-3.956951	1.420034	5.000590
C	-3.104780	2.251957	5.785689
C	-2.410311	3.269375	5.196193
O	-0.330049	-0.547242	0.548628
O	0.839070	1.102994	-1.966819
C	2.081018	1.296121	-2.029811
O	2.724632	2.081579	-2.712817
C	2.903206	0.398913	-1.094837
N	2.298154	-0.578562	-0.449005
C	2.945000	-1.497565	0.397113
C	4.244704	-1.926330	0.139898
C	4.876249	-2.786332	1.037123
C	4.220700	-3.230907	2.178393
C	2.908226	-2.813063	2.409494
C	2.262904	-1.954070	1.530951
H	1.267332	-0.513965	-0.347436
H	-6.578098	-1.717014	0.739015
H	-7.841598	-3.771182	1.196675
H	-6.958056	-5.997398	0.470566
H	-4.756368	-6.139119	-0.664554
H	-2.718816	-5.076958	-1.469799
H	-5.938951	3.441193	0.560758
H	-6.318292	-1.436777	-2.113573
H	-8.422055	-0.568994	-3.026147
H	-9.270382	1.686508	-2.348838
H	-7.939940	3.084972	-0.789280
H	2.813437	-3.350996	-3.068722
H	-1.160849	5.192509	3.784811
H	-3.423706	3.574428	-1.047917
H	-2.137848	5.402593	-2.053984
H	-0.687495	6.875133	-0.637259
H	-0.537231	6.486362	1.803462
H	-4.748573	0.982486	3.071660
H	-4.510219	0.609068	5.482929
H	-3.013006	2.070186	6.860408
H	-1.755872	3.915148	5.789924
H	-2.498280	-1.787584	-3.704194
H	-1.743411	-0.709963	-5.766027
H	0.672266	-0.761200	-6.427613
H	2.314492	-1.963464	-5.018323
H	3.349954	-4.759961	-1.149664
H	2.681501	-5.818597	0.987413
H	0.364931	-5.474726	1.877216
H	-1.282766	-4.187333	0.613747
H	-0.348781	1.575927	-1.284132
H	1.232853	-1.630754	1.698842
H	4.718358	-3.908455	2.874946
H	4.762517	-1.618108	-0.771193
H	3.970495	0.316121	-1.300349
C	2.202398	-3.309519	3.647419
F	2.719830	-2.761022	4.753725
F	0.900737	-3.034435	3.636089
F	2.332924	-4.637177	3.780399
C	6.261789	-3.271445	0.696611
F	6.841136	-3.912279	1.710072
F	6.234204	-4.112861	-0.346456
F	7.064391	-2.255983	0.349505
C	6.204527	4.023782	-0.044673
C	5.474101	2.827896	-0.122688
C	6.175862	1.612380	-0.137241
C	7.567696	1.590933	-0.109248
C	8.281688	2.786469	-0.046502
C	7.594310	4.000687	-0.004089
C	4.003742	2.830490	-0.035492
C	3.262526	1.858185	0.601531
C	1.883419	2.387269	0.896503
C	1.803276	3.656586	0.032032
N	3.184160	3.848552	-0.471356
C	3.426572	4.629303	-1.605596
O	2.335858	5.337646	-1.891470
C	2.376936	6.082695	-3.097534
O	4.460986	4.690482	-2.211589
H	3.739874	1.106258	1.233651
H	1.076406	1.672957	0.673296
H	1.120290	3.542688	-0.819098
H	1.486209	4.544735	0.597930

H	2.512414	5.409415	-3.959199
H	1.405898	6.592395	-3.168163
H	3.193287	6.822747	-3.076678
H	1.798780	2.626405	1.970675
H	5.637620	0.662413	-0.166157
H	8.092444	0.632061	-0.131164
H	9.375154	2.772994	-0.023223
H	8.146951	4.942120	0.059869
H	5.681142	4.979870	0.001225

SiSi-cTC

Zero-point correction= 1.025156
(Hartree/Particle)
Thermal correction to Energy= 1.094909
Thermal correction to Enthalpy= 1.095853
Thermal correction to Gibbs Free Energy= 0.916209
Sum of electronic and zero-point Energies= -
4342.352397
Sum of electronic and thermal Energies= -
4342.282644
Sum of electronic and thermal Enthalpies= -
4342.281700
Sum of electronic and thermal Free Energies= -
4342.461344
Quasi-Harmonic Approximation corrected Free energy= -4342.444255
E(wB97XD/Def2TZVPP)= -4348.00974744

C	3.770853	0.906878	-3.651845
C	3.580571	1.954530	-2.691956
C	2.245607	2.393449	-2.390394
C	1.156970	1.757067	-3.076686
C	1.379619	0.768405	-3.993024
C	2.706546	0.333430	-4.286632
C	2.050503	3.404771	-1.427020
C	3.149998	3.990445	-0.770609
C	4.481088	3.562818	-1.098748
C	4.664664	2.549293	-2.041990
C	5.592220	4.170715	-0.429232
C	5.400159	5.134282	0.519313
C	4.077964	5.552352	0.856944
C	2.992746	5.003742	0.232691
C	0.659666	3.843436	-1.110320
C	-0.036325	3.281036	-0.006224
C	-1.363891	3.546188	0.268530
C	-2.084084	4.415086	-0.621636
C	-1.381458	5.044131	-1.690807
C	-0.011672	4.738831	-1.906087
C	-2.076536	5.925175	-2.564765
C	-3.421567	6.154031	-2.412062
C	-4.132610	5.494081	-1.381709
C	-3.484674	4.648464	-0.513041
O	0.663366	2.436306	0.821447
P	0.398620	0.840070	0.791037
O	0.732559	0.301713	2.158180
C	-2.057130	2.829939	1.377541
C	-2.047826	1.447371	1.378070
C	-2.895772	0.665611	2.206979
C	-3.652610	1.318791	3.149519
C	-3.583706	2.729593	3.309015
C	-2.787690	3.500572	2.412569
C	-2.726296	4.909299	2.599782
C	-3.432341	5.517795	3.609846
C	-4.242005	4.753546	4.484472
C	-4.312510	3.389932	4.336265
O	-1.222307	0.780907	0.512283
C	-3.063698	-0.801024	1.961758
C	-2.349496	-1.759621	2.707720
C	-2.631771	-3.159111	2.525426
C	-3.602496	-3.551406	1.600294
C	-4.291867	-2.612450	0.828898
C	-4.009507	-1.213103	0.996458
C	-1.903463	-4.124388	3.297534
C	-0.931421	-3.732198	4.175045
C	-0.635263	-2.345979	4.342215
C	-1.328105	-1.393720	3.646065
C	-4.717822	-0.282031	0.165126
C	-5.642045	-0.709211	-0.747280
C	-5.935883	-2.096473	-0.891796
C	-5.277987	-3.018032	-0.128108
O	1.011184	0.151708	-0.401704
O	2.117418	-1.736478	2.441823
C	1.771507	-2.820617	1.850577
O	2.370830	-3.870959	1.850002
C	0.480344	-2.936517	1.067875
N	0.225868	-2.293350	-0.010281
C	-0.872177	-2.537008	-0.885354
C	-1.445757	-3.799717	-1.006439
C	-2.532505	-3.963897	-1.862113
C	-3.045722	-2.887822	-2.581409
C	-2.447506	-1.636291	-2.456673
C	-1.352013	-1.454859	-1.621918
H	0.779175	-1.398933	-0.243118
H	-4.056629	4.141092	0.265699
H	-5.209759	5.653463	-1.278072
H	-3.947688	6.829850	-3.092176
H	-1.518075	6.408241	-3.372262
H	0.505209	5.193104	-2.756401
H	-4.325939	0.744610	3.792791
H	-2.097357	5.508482	1.937162
H	-3.363895	6.601243	3.742901
H	-4.801823	5.250881	5.281553
H	-4.926636	2.787969	5.012913
H	5.678890	2.205111	-2.266797
H	-3.826516	-4.614815	1.476649
H	-1.084041	-0.338257	3.775081
H	0.154788	-2.044180	5.034986
H	-0.374274	-4.476362	4.751066
H	-2.139722	-5.184746	3.163413
H	-4.506285	0.785317	0.259438
H	-6.159020	0.019376	-1.377457
H	-6.683042	-2.417804	-1.623036
H	-5.487996	-4.086010	-0.238164
H	1.985652	5.335647	0.497971
H	3.935815	6.320610	1.622625
H	6.256022	5.587785	1.027705
H	6.600630	3.834683	-0.688272
H	4.791917	0.573510	-3.860160
H	2.864651	-0.465693	-5.016931
H	0.532404	0.297016	-4.498914
H	0.133175	2.065333	-2.852012
H	1.501915	-0.887673	2.326851
H	-0.875220	-0.478825	-1.524461
H	-3.907127	-3.025147	-3.236123
H	-1.045718	-4.657211	-0.460147
H	-0.187167	-3.745932	1.387853
C	-2.949463	-0.456216	-3.254137
F	-2.898564	0.674515	-2.549715
F	-2.203982	-0.265712	-4.352401
F	-4.208086	-0.629349	-3.662349
C	-3.141589	-5.337279	-2.003331
F	-2.351811	-6.151697	-2.708833
F	-3.324303	-5.915345	-0.808340
F	-4.326713	-5.300056	-2.612396
C	6.391261	-0.904185	0.363016
C	4.996280	-0.915246	0.228621
C	4.262423	0.178355	0.699193
C	4.901945	1.252552	1.313437
C	6.289009	1.252978	1.452091
C	7.032351	0.174500	0.966402
C	4.304552	-2.028590	-0.453872
C	3.293926	-1.923585	-1.334989
C	2.957844	-3.269244	-1.925908
C	4.137466	-4.142521	-1.457777
N	4.740721	-3.378191	-0.360688
C	5.149869	-4.049620	0.767520
O	5.333047	-5.245486	0.801727
O	5.338072	-3.230974	1.800494
C	5.650082	-3.834006	3.038008
H	2.886956	-3.236628	-3.025520

H	4.877697	-4.277363	-2.266263
H	1.986770	-3.655556	-1.557217
H	3.843679	-5.136799	-1.096182
H	2.829297	-0.980158	-1.624894
H	4.833847	-4.499554	3.362287
H	6.588750	-4.409871	2.977274
H	5.762949	-3.006527	3.753037
H	3.178410	0.179714	0.582374
H	4.308152	2.095028	1.679501
H	6.792134	2.096039	1.934931
H	8.122259	0.170365	1.065289
H	6.976493	-1.750825	-0.006786

SiSi-cTS

Zero-point correction= 1.025858 (Hartree/Particle)
Thermal correction to Energy= 1.094691
Thermal correction to Enthalpy= 1.095635
Thermal correction to Gibbs Free Energy= 0.917874
Sum of electronic and zero-point Energies= -4342.340105
Sum of electronic and thermal Energies= -4342.271272
Sum of electronic and thermal Enthalpies= -4342.270328
Sum of electronic and thermal Free Energies= -4342.448089
Quasi-Harmonic Approximation corrected Free energy= -4342.431043
E(wB97XD/Def2TZVPP)= -4347.99575133

C	-0.835018	4.162467	-3.816182
C	-1.746358	4.225575	-2.711189
C	-2.405250	3.022650	-2.281504
C	-2.098852	1.800613	-2.969144
C	-1.214530	1.779587	-4.009702
C	-0.575126	2.978566	-4.444764
C	-3.309187	3.076120	-1.201321
C	-3.535936	4.284044	-0.514481
C	-2.860184	5.478637	-0.939843
C	-1.992020	5.423330	-2.034063
C	-3.099563	6.701139	-0.231003
C	-3.951481	6.739714	0.835897
C	-4.621770	5.554112	1.261690
C	-4.423454	4.369998	0.608966
C	-4.047785	1.838646	-0.814640
C	-3.537310	0.973872	0.191178
C	-4.136665	-0.228522	0.521408
C	-5.298955	-0.639177	-0.219208
C	-5.854624	0.247209	-1.187068
C	-5.206795	1.480474	-1.457379
C	-7.019546	-0.137321	-1.907078
C	-7.599674	-1.365112	-1.705502
C	-7.023126	-2.268572	-0.781231
C	-5.906040	-1.918083	-0.061037
O	-2.416252	1.392878	0.857720
P	-0.976253	0.651377	0.725701
O	-0.324137	0.705822	2.085797
C	-3.512122	-1.133225	1.528141
C	-2.198125	-1.516753	1.340732
C	-1.575040	-2.553608	2.085353
C	-2.284943	-3.118170	3.117002
C	-3.590308	-2.667036	3.454217
C	-4.218133	-1.666228	2.656660
C	-5.512728	-1.213054	3.033797
C	-6.155231	-1.742182	4.127663
C	-5.542033	-2.756100	4.902289
C	-4.286456	-3.204326	4.571888
O	-1.451445	-0.888711	0.381190
C	-0.230169	-3.072038	1.687353
C	0.927142	-2.714423	2.407023
C	2.189952	-3.313222	2.066123
C	2.253920	-4.239723	1.023355
C	1.119038	-4.581193	0.283269
C	-0.144003	-3.977068	0.606083
C	3.360490	-2.942024	2.807438
C	3.290560	-2.024429	3.816749
C	2.041159	-1.418411	4.147159
C	0.901133	-1.753520	3.472419
C	-1.280515	-4.332078	-0.194271
C	-1.170143	-5.223975	-1.224131
C	0.080313	-5.838516	-1.525079
C	1.189557	-5.523864	-0.793772
O	-0.146649	1.084684	-0.448286
O	1.978089	1.509583	2.394899
C	3.074080	0.890359	2.110354
O	4.106970	1.019707	2.732687
C	3.146989	-0.044290	0.908547
N	2.149066	-0.327326	0.102581
C	2.182049	-1.304298	-0.916875
C	3.318015	-2.068475	-1.179801
C	3.290274	-3.011762	-2.205067
C	2.146560	-3.202570	-2.972443
C	1.020456	-2.423924	-2.706772
C	1.028307	-1.480209	-1.689203
H	1.320610	0.308737	0.030057
H	-5.473001	-2.637784	0.635509
H	-7.466369	-3.258845	-0.642158
H	-8.492762	-1.653614	-2.267035
H	-7.437423	0.563117	-2.636574
H	-5.618999	2.139450	-2.227089
H	-1.835947	-3.926637	3.701452
H	-5.993090	-0.424219	2.450295
H	-7.146596	-1.372780	4.405397
H	-6.065798	-3.172164	5.767592
H	-3.797157	-3.978408	5.170929
H	-1.489696	6.338373	-2.364766
H	3.213323	-4.703211	0.774941
H	-0.042067	-1.262552	3.716080
H	2.003555	-0.665000	4.938358
H	4.189369	-1.737890	4.369079
H	4.313279	-3.414248	2.546536
H	-2.248653	-3.874165	0.020533
H	-2.048285	-5.466655	-1.828758
H	0.145685	-6.553183	-2.350319
H	2.156889	-5.981175	-1.020372
H	-4.940392	3.467421	0.944767
H	-5.297689	5.597174	2.120616
H	-4.123537	7.678349	1.370682
H	-2.580706	7.605848	-0.562923
H	-0.341214	5.084555	-4.138224
H	0.128753	2.941762	-5.281349
H	-0.983944	0.834015	-4.507724
H	-2.568839	0.871272	-2.639362
H	1.011988	1.122100	2.185295
H	0.150842	-0.862086	-1.495210
H	2.132012	-3.944290	-3.772909
H	4.236001	-1.939365	-0.605663
H	3.942753	-0.780969	1.010227
C	-0.202623	-2.557165	-3.581224
F	-1.317586	-2.193379	-2.951423
F	-0.095950	-1.781660	-4.672858
F	-0.368032	-3.809179	-4.018460
C	4.512059	-3.864112	-2.433071
F	5.639705	-3.168213	-2.249093
F	4.557111	-4.897954	-1.576337
F	4.547462	-4.374940	-3.662720
C	4.268315	4.690069	-0.590306
C	3.772394	3.393155	-0.383131
C	2.429979	3.124895	-0.664686
C	1.581181	4.136175	-1.105744
C	2.078651	5.421911	-1.305601
C	3.427208	5.693485	-1.057642
C	4.678453	2.318005	0.056375
C	4.720666	1.033982	-0.448150
C	6.035622	0.397043	-0.062569
C	6.607900	1.358468	0.994292
N	5.691093	2.511194	0.965471
C	5.686173	3.336077	2.097965
O	6.610494	3.356190	2.867510

O	4.576820	4.042642	2.203509
C	4.417381	4.817076	3.381534
H	6.689299	0.334035	-0.949823
H	7.633269	1.689144	0.770656
H	5.945110	-0.630210	0.331744
H	6.594633	0.926627	2.004001
H	4.187948	0.767281	-1.363353
H	4.386008	4.160656	4.265691
H	5.236680	5.546316	3.487479
H	3.456725	5.336764	3.265655
H	2.008629	2.133216	-0.517921
H	0.529817	3.901880	-1.287954
H	1.415408	6.218332	-1.655749
H	3.824900	6.698920	-1.222758
H	5.320904	4.909760	-0.391875
